# Supplementary material for: Value-based person-centred integrated care for frail elderly living at home: a quasi-experimental evaluation using multicriteria decision analysis
Source: BMJ Open. 2022 Apr 15;12(4):e054672. doi: 10.1136/bmjopen-2021-054672 (PMC9016393; doi:10.1136/bmjopen-2021-054672)
Supplement: Supplementary data [file bmjopen-2021-054672supp001.pdf]

Supplementary files

Appendix 1: Equation relative standardization 2

Appendix 2. Relative DCE weights (0-1) (SE) of the outcomes used in the MCDA by type of stakeholder 3

Appendix 3: Graph common support 4

Appendix 4: Predicted mean performance scores of all outcome measures on their natural scale...... 5

Appendix 5: Comparison of baseline characteristics and outcome scores of dropouts versus non-dropouts........ 6

Appendix 6a: Value scores in the Multi-Criteria Decision Analysis at 6 months without costs 8

Appendix 6b: Value scores in the Multi-Criteria Decision Analysis at 12 months without costs 9

Hoedemakers M, *et al. BMJ Open* 2022; 12:e054672. doi: 10.1136/bmjopen-2021-054672

Appendix 1: Equation relative standardization

$$S_{aj} = \frac{x_{aj}}{\left(x_{aj}^2 + x_{bj}^2\right)^{1/2}}$$

Where

x = predicted mean score on the natural scale

a = the intervention group

b = the control group

j = outcome measure *j*

Note: For all outcomes in the MCDA, a higher score indicates better performance. To achieve this, in the above-mentioned equation, x is replaced by $1/x$ for outcomes where a higher score on the natural scale indicates a worse performance (e.g., costs).

Appendix 2. Relative DCE weights (0-1) (SE) of the outcomes used in the MCDA by type of stakeholder

	**Patients (n=156)**	**Partners (n=158)**	**Professionals (n=155)**	**Payers (n=104)**	**Policymakers (n=151)**
Physical functioning	0.16 (0.15)	0.11 (0.12)	0.12 (0.12)	0.14 (0.13)	0.14 (0.14)
Psychological well-being	0.17 (0.15)	0.15 (0.14)	0.18 (0.16)	0.18 (0.17)	0.15 (0.15)
Enjoyment of life	0.23 (0.18)	0.25 (0.19)	0.22 (0.19)	0.24 (0.23)	0.22 (0.19)
Social participation & relationships	0.08 (0.10)	0.09 (0.11)	0.11 (0.11)	0.10 (0.11)	0.10 (0.10)
Resilience	0.15 (0.13)	0.14 (0.13)	0.13 (0.12)	0.11 (0.10)	0.14 (0.13)
Person-centeredness	0.08 (0.10)	0.08 (0.10)	0.08 (0.09)	0.06 (0.08)	0.08 (0.09)
Continuity of care	0.10 (0.11)	0.12 (0.12)	0.11 (0.11)	0.08 (0.09)	0.10 (0.10)
Total health and social care costs	0.03 (0.08)	0.06 (0.09)	0.06 (0.09)	0.08 (0.08)	0.07 (0.09)

Note: Numbers in parentheses (n) by stakeholders indicate the number of participants included in the weight elicitation study

Hoedemakers M, *et al. BMJ Open* 2022; 12:e054672. doi: 10.1136/bmjopen-2021-054672

Appendix 3: Graph common support

Graph 1: Common Support of the propensity scores when matching includes the baseline values of the following variables: age, gender, marital status, living situation, educational level, smoking status, and total health and social care costs (minus medication) of three months prior to baseline as a proxy for severity

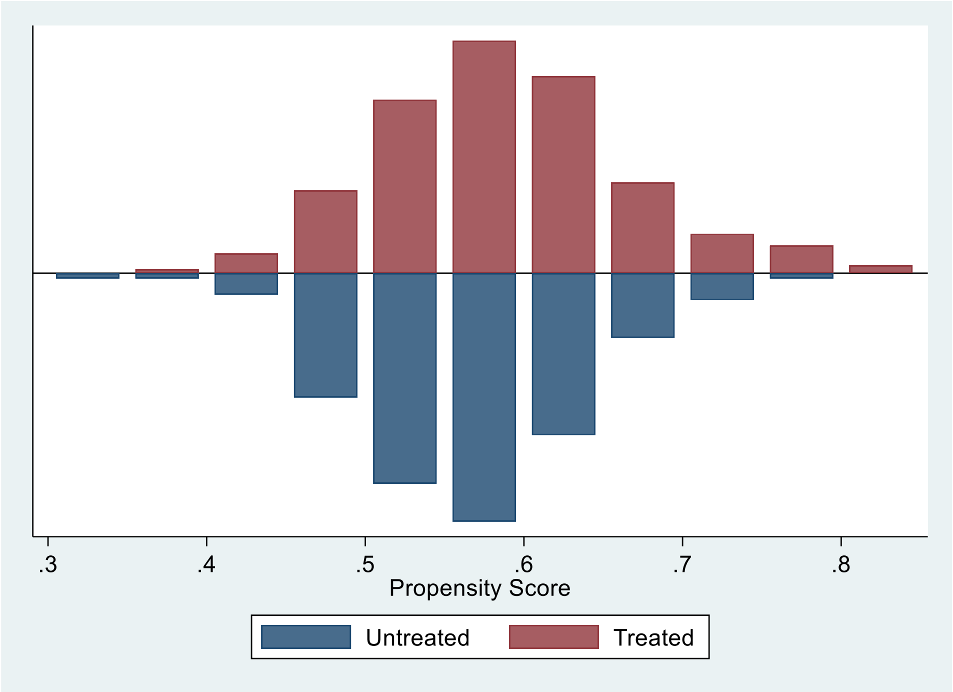

Appendix 4: Predicted mean performance scores of all outcome measures on their natural scale

	T0#		T1		T2	
**Outcomes measures**	**CCFE (n=222)**	**UC (n=162)**	**CCFE (n=172)**	**UC (n=129)**	**CCFE (n=156)**	**UC (n=113)**
**Health & Well-being**						
Physical functioning (0-15) ^	4.383	4.383	5.126	4.653	5.715	5.328
Psychological well-being (0-100)	71.351	71.351	70.707	71.010	69.998	71.256
Enjoyment of life (1-4)	2.820	2.820	2.917	2.741	2.874	2.839
Social relationships & participation (7-35) ^	9.167	9.167	9.273	9.579	9.424	9.779
Resilience (6-30)	19.317	19.317	19.299	19.108	19.347	19.426
**Experience of care**						
Person-centeredness (0-18)	11.729	11.729	12.828	11.353	13.060	11.727
Continuity of care (1-5)	3.672	3.672	3.771	3.653	3.833	3.704
**Costs**						
Total health and social care costs ^	5453	5453	10858	10199	22676	20659
**Additional frail elderly-specific outcomes**						
Autonomy (7-35)	22.284	22.284	21.785	22.306	21.790	22.834
Burden of medication (0-10) ^	2.088	2.088	2.221	1.878	2.263	1.480

# Note that the intervention coefficient was included in the calculation of the mean performance scores in the control group, assuming the control group had the same baseline scores as the intervention group to make it possible to directly compare the intervention and control group. CCFE=Care Chain Frail Elderly. UC=Usual care.

5

Appendix 5: Comparison of baseline characteristics and outcome scores of dropouts versus non-dropouts

	Across CCFE and UC			CCFE separately			UC separately		
**Baseline characteristics**	**Dropouts (n=115)**	**Non-dropouts (n=269)**	**P-value**	**Dropouts (n=66)**	**Non-dropouts (n=156)**	**P-value**	**Dropouts (n=49)**	**Non-dropouts (n=113)**	**P-value**
**Intervention group**	66 (57%)	156 (58%)	0.913	-	-	-	-	-	-
**Age**	85.00	83.56	0.036*	83.91	83.21	0.449	86.47	84.05	0.016*
**Gender** (female)	61%	65%	0.434	62%	64%	0.779	59%	66%	0.381
**Living situation**			0.926			0.842			0.711
Independent, alone	57%	57%		53%	54%		63%	60%	
With other(s)	43%	43%		47%	46%		37%	40%	
**Marital status**			0.162			0.149			0.734
Single, never married	1%	5%		0%	5%		2%	4%	
Married or living together	41%	44%		44%	45%		37%	42%	
Widow(er)	53%	45%		52%	42%		55%	49%	
Divorced	5%	7%		5%	8%		6%	4%	
**Educational level**			0.923			0.839			0.981
Low	70%	71%		68%	71%		71%	70%	
Middle	19%	17%		23%	19%		14%	15%	
High	11%	12%		9%	10%		14%	15%	
**Smoking** (yes)	15%	11%	0.269	18%	13%	0.299	10%	8%	0.641
**Baseline outcome scores**									
Physical functioning (0-15) ^	5.18	4.10	0.001*	4.89	4.17	0.081	5.56	4.02	0.003*
Psychological well-being (0-100)	69.37	72.39	0.145	68.06	72.74	0.092	71.17	71.89	0.815
Enjoyment of life (1-4)	2.81	2.89	0.351	2.74	2.85	0.349	2.90	2.95	0.725

Hoedemakers M, *et al. BMJ Open* 2022; 12:e054672. doi: 10.1136/bmjopen-2021-054672

	Across CCFE and UC			CCFE separately			UC separately		
**Baseline characteristics**	**Dropouts (n=115)**	**Non-dropouts (n=269)**	**P-value**	**Dropouts (n=66)**	**Non-dropouts (n=156)**	**P-value**	**Dropouts (n=49)**	**Non-dropouts (n=113)**	**P-value**
Social relationships and participation (7-35) ^	8.86	8.55	0.461	9.48	9.03	0.451	8.00	7.88	0.831
Resilience (6-30)	18.78	19.38	0.212	18.97	19.46	0.448	18.52	19.27	0.298
Autonomy (7-35)	21.81	22.35	0.245	21.89	22.45	0.386	21.69	22.21	0.452
Person-centeredness (0-18)	12.29	11.97	0.473	11.86	11.66	0.730	12.88	12.4	0.481
Continuity of care (1-5)	3.70	3.72	0.838	3.65	3.69	0.743	3.78	3.77	0.939
Burden of medication (0-10) ^	2.17	2.24	0.809	2.12	2.07	0.908	2.22	2.47	0.604
Total costs 3 months prior to the study (€) ^	5,989	5,112	0.247	5,797	5,308	0.635	6,248	4,842	0.208

*^ Higher score indicating worse performance. * p<0.05. CCFE=Care Chain Frail Elderly. UC=Usual care.*

Appendix 6a: Value scores in the Multi-Criteria Decision Analysis at 6 months without costs

			Patients		Partners		Professionals		Payers		Policymakers	
**Outcome measures**	**Standardised performance score#**		**Weighted score**		**Weighted score**		**Weighted score**		**Weighted score**		**Weighted score**	
	**CCFE**	**UC**	**CCFE**	**UC**	**CCFE**	**UC**	**CCFE**	**UC**	**CCFE**	**UC**	**CCFE**	**UC**
Physical functioning	0.672	0.740	0.111	0.122	0.079	0.087	0.085	0.094	0.103	0.114	0.101	0.111
Psychological well-being	0.706	0.709	0.124	0.124	0.113	0.113	0.134	0.134	0.140	0.140	0.114	0.114
Enjoyment of life	0.729	0.685	0.173	0.162	0.194	0.182	0.169	0.159	0.192	0.181	0.172	0.162
Social relationships and participation	0.718	0.696	0.059	0.057	0.069	0.067	0.083	0.081	0.079	0.076	0.077	0.075
Resilience	0.711	0.704	0.110	0.109	0.106	0.105	0.097	0.096	0.086	0.085	0.107	0.106
Person-centeredness	0.749	0.663	0.062	0.055	0.064	0.056	0.063	0.056	0.049	0.044	0.064	0.057
Continuity of care	0.718	0.696	0.074	0.072	0.092	0.089	0.083	0.081	0.063	0.061	0.077	0.075
**Overall value scores**			**0.712**	**0.701**	**0.715**	**0.698**	**0.714**	**0.700**	**0.713**	**0.701**	**0.713**	**0.700**

# colour scheme ranges from red (lowest score) to green (highest score). CCFE=Care Chain Frail Elderly. UC=Usual care.

Hoedemakers M, *et al. BMJ Open* 2022; 12:e054672. doi: 10.1136/bmjopen-2021-054672

Appendix 6b: Value scores in the Multi-Criteria Decision Analysis at 12 months without costs

			Patients		Partners		Professionals		Payers		Policymakers	
**Outcome measures**	**Standardised performance score[#]**		**Weighted score**		**Weighted score**		**Weighted score**		**Weighted score**		**Weighted score**	
	**CCFE**	**UC**	**CCFE**	**UC**	**CCFE**	**UC**	**CCFE**	**UC**	**CCFE**	**UC**	**CCFE**	**UC**
Physical functioning	0.682	0.731	0.112	0.121	0.080	0.086	0.086	0.092	0.105	0.113	0.103	0.110
Psychological well-being	0.701	0.713	0.123	0.125	0.112	0.114	0.133	0.135	0.139	0.141	0.113	0.115
Enjoyment of life	0.711	0.703	0.169	0.167	0.189	0.187	0.165	0.163	0.188	0.185	0.168	0.166
Social relationships and participation	0.720	0.694	0.059	0.057	0.069	0.066	0.083	0.080	0.079	0.076	0.077	0.075
Resilience	0.706	0.709	0.109	0.110	0.105	0.106	0.097	0.097	0.085	0.086	0.106	0.107
Person-centeredness	0.744	0.668	0.061	0.055	0.063	0.057	0.063	0.056	0.049	0.044	0.064	0.057
Continuity of care	0.719	0.695	0.074	0.072	0.092	0.089	0.083	0.080	0.063	0.061	0.077	0.075
**Overall value scores**			**0.708**	**0.706**	**0.710**	**0.704**	**0.710**	**0.704**	**0.708**	**0.706**	**0.709**	**0.705**

[#] *colour scheme ranges from red (lowest score) to green (highest score). CCFE=Care Chain Frail Elderly. UC=Usual care.*

Hoedemakers M, *et al. BMJ Open* 2022; 12:e054672. doi: 10.1136/bmjopen-2021-054672
