# Supplementary material for: Value-based person-centred integrated care for frail elderly living at home: a quasi-experimental evaluation using multicriteria decision analysis
Source: BMJ Open. 2022 Apr 15;12(4):e054672. doi: 10.1136/bmjopen-2021-054672 (PMC9016393; doi:10.1136/bmjopen-2021-054672)
Supplement: Author's manuscript [file bmjopen-2021-054672.draft_revisions.pdf]

BMJ Open is committed to open peer review. As part of this commitment we make the peer review history of every article we publish publicly available.

When an article is published we post the peer reviewers' comments and the authors' responses online. We also post the versions of the paper that were used during peer review. These are the versions that the peer review comments apply to.

The versions of the paper that follow are the versions that were submitted during the peer review process. They are not the versions of record or the final published versions. They should not be cited or distributed as the published version of this manuscript.

BMJ Open is an open access journal and the full, final, typeset and author-corrected version of record of the manuscript is available on our site with no access controls, subscription charges or pay-per-view fees (http://bmjopen.bmj.com).

If you have any questions on BMJ Open's open peer review process please email info.bmjopen@bmj.com

Value-based person-centred integrated care for frail elderly living at home: a quasi-experimental evaluation using Multi-Criteria Decision Analysis

Journal:	*BMJ Open*
Manuscript ID	bmjopen-2021-054672
Article Type:	Original research
Date Submitted by the Author:	22-Jun-2021

Complete List of Authors:	Hoedemakers, Maaike; Erasmus Universiteit Rotterdam, Erasmus School of Health Policy & Management<br>Karimi, Milad; Erasmus University Rotterdam<br>Leijten, Fenna; Erasmus Universiteit Rotterdam, Health Policy & Management ; Ministry of defence, Staff Defence Healthcare Organisation<br>Goossens, Lucas; Erasmus University<br>Islam, Kamrul; University of Bergen, Department of Economics; NORCE Norwegian Research Centre AS, Social sciences<br>Tsiachristas, Apostolos; University of Oxford, Nuffield Department of Population Health<br>Rutten-van Molken, Maureen; Erasmus University Rotterdam; Erasmus University Rotterdam

Keywords:	Health economics < HEALTH SERVICES ADMINISTRATION & MANAGEMENT, HEALTH ECONOMICS, Health policy < HEALTH SERVICES ADMINISTRATION & MANAGEMENT, Organisation of health services < HEALTH SERVICES ADMINISTRATION & MANAGEMENT

SCHOLARONE™
Manuscripts

I, the Submitting Author has the right to grant and does grant on behalf of all authors of the Work (as defined in the below author licence), an exclusive licence and/or a non-exclusive licence for contributions from authors who are: i) UK Crown employees; ii) where BMJ has agreed a CC-BY licence shall apply, and/or iii) in accordance with the terms applicable for US Federal Government officers or employees acting as part of their official duties; on a worldwide, perpetual, irrevocable, royalty-free basis to BMJ Publishing Group Ltd ("BMJ") its licensees and where the relevant Journal is co-owned by BMJ to the co-owners of the Journal, to publish the Work in this journal and any other BMJ products and to exploit all rights, as set out in our licence.

The Submitting Author accepts and understands that any supply made under these terms is made by BMJ to the Submitting Author unless you are acting as an employee on behalf of your employer or a postgraduate student of an affiliated institution which is paying any applicable article publishing charge ("APC") for Open Access articles. Where the Submitting Author wishes to make the Work available on an Open Access basis (and intends to pay the relevant APC), the terms of reuse of such Open Access shall be governed by a Creative Commons licence – details of these licences and which Creative Commons licence will apply to this Work are set out in our licence referred to above.

Other than as permitted in any relevant BMJ Author's Self Archiving Policies, I confirm this Work has not been accepted for publication elsewhere, is not being considered for publication elsewhere and does not duplicate material already published. I confirm all authors consent to publication of this Work and authorise the granting of this licence.

For peer review only

Title

Value-based person-centred integrated care for frail elderly living at home: a quasi-experimental evaluation using Multi-Criteria Decision Analysis

Authors

Maaike Hoedemakers, MSc[1]; Milad Karimi, PhD[1,2]; Fenna Leijten, PhD[1,3]; Lucas Goossens, PhD [1]; Kamrul Islam, PhD [4,5]; Apostolos Tsiachristas, PhD [6]; Maureen Rutten-van Mölken, PhD [1,7]

Affiliations

1: Erasmus School of Health Policy & Management, Erasmus University Rotterdam, the Netherlands

2: Pharmerit, Rotterdam, the Netherlands

3: Staff Defence Healthcare Organisation, Ministry of Defence, Utrecht, the Netherlands

4: Department of Economics, University of Bergen, Bergen, Norway

5: Health Services and Health Economics, NORCE Norwegian Research Centre, Bergen, Norway

6: Health Economics Research Centre, Nuffield Department of Population Health, University of Oxford, Oxford, United Kingdom

7: Institute for Medical Technology Assessment, Erasmus University Rotterdam, Rotterdam, the Netherlands

Contact information corresponding author

Maureen Rutten-van Mölken

Burgemeester Oudlaan 50, 3062 PA Rotterdam

T: +31 10 408 9056

E-mail: m.rutten@eshpm.eur.nl

For peer review only

Word count: 3680

Abstract

Objective: To evaluate the value of the person-centred, integrated care programme Care Chain Frail Elderly (CCFE) compared with usual care, using Multi-Criteria Decision Analysis (MCDA).

Design: In a 12-month quasi-experimental study, Triple-Aim outcomes were measured at 0, 6 and 12 months by trained interviewers during home-visits.

Setting: Primary care, community-based elderly care.

Participants: 384 community-dwelling frail elderly were enrolled. The 12-month completion rate was 70% in both groups. Propensity score matching was used to balance age, gender, marital status, living situation, education, smoking status, and three-month costs prior to baseline between the two groups.

Intervention: The CCFE is an integrated care programme with unique features like the presence of the elderly and informal caregiver at the multi-disciplinary team meetings, and a bundled payment.

Primary and secondary outcomes measures: The MCDA results in weighted overall value scores that combines the performance on physical functioning, psychological well-being, social relationships and participation, enjoyment of life, resilience, person-centeredness, continuity of care, and costs, with importance weights of patients, informal caregivers, professionals, payers, and policymakers.

Results: At 6 months, the overall value scores of CCFE were higher in all stakeholder groups, driven by enjoyment of life (standardised performance scores 0.729 versus 0.685) and person-centeredness (0.749 versus 0.663). At 12 months, the overall value scores in both groups were similar from a patient's perspective, slightly higher for CCFE from an informal caregiver's and professional's perspective, and

For peer review only - http://bmjopen.bmj.com/site/about/guidelines.xhtml

lower for CCFE from a payer's and policymaker's perspective. The latter was driven by a worse performance on physical functioning (0.682 versus 0.731) and higher costs (€22,816 versus €20,680).

Conclusions: The MCDA indicated that the CCFE is the preferred way of delivering care to frail elderly at 6 months. However, at 12 months, MCDA-results showed little difference from the perspective of patients, informal caregivers, and professionals, while payers and policy makers seemed to prefer usual care.

Key words: Multi-Criteria Decision Analysis, integrated care, frail elderly, triple aim, patient-reported outcome measures

For peer review only

For peer review only - http://bmjopen.bmj.com/site/about/guidelines.xhtml

Strengths and limitations of this study

- We implemented a novel multi-criteria decision analysis (MCDA) framework to evaluate a person-centred integrated care programme for frail elderly.
- We measured a broad range of Triple-Aim outcomes at three time points, among a large and difficult to reach group of the frailest elderly living at home.
- The choice of outcome measures was largely driven by focus groups involving frail elderly.
- The different outcome measures were weighted by their importance, from multiple perspectives, to calculate overall value scores.
- Due to the choice of measuring patient-reported outcomes, participation was too burdensome or impossible for frail elderly with cognitive impairments.

For peer review only

INTRODUCTION

The Care Chain Frail Elderly (CCFE) is an integrated care programme for community-dwelling frail elderly in the Netherlands that offers person-centred care, coordination, and case management to support elderly in living at home for as long as possible.[1] It was developed as part of a movement towards decentralization of long-term care, increased self-sufficiency, and societal participation that was stimulated by the Dutch government to maintain affordability of elderly care. This movement led to a reform of the long-term care sector in 2015.[2] The number of nursing- and residential homes was reduced considerably and access to nursing homes was restricted to those in need of 24-hour care. Municipalities became responsible for the provision of homecare and social support, and health insurers for financing nursing care at home. While the importance of homecare was growing, this sector was confronted with significant budget cuts.[3] The reform accelerated the development of integrated-care initiatives, which were spearheaded by the National Care for the Elderly-Programme commissioned by the Ministry of Health.[4,5]

Integrated care for older people often comprises similar components, namely, comprehensive assessment, individualised care planning, case management, and multidisciplinary team meetings.[6,7] All of these elements are also present in the CCFE. In addition, the intervention is unique in several respects. First, the elderly and their informal caregiver are present at the multidisciplinary team meetings where the results of the comprehensive assessment and the individual care plan are discussed. Second, the CCFE is financed by a bundled payment aiming to stimulate collaboration between professionals. The bundled payment is a fixed amount of money per patient that covers all services provided by the GP, nurse-practitioner and physician assistant, regardless of diagnoses, medication review by the pharmacist, telephone consultation by the geriatrician, non-individual-patient-related activities such as building a community network, and overhead. Third, it targets the top-1% frailest elderly registered in a GP-practice who are living at home with complex care needs, using a case-finding approach. The CCFE aims

For peer review only

to better integrate care across sectors and build a network of support around the patient, and thereby to improve their physical, mental, social health and well-being and experience with care. Ideally, this also reduces secondary care and residential long-term care utilization and thereby costs. The CCFE has previously been described in detail elsewhere.[1]

Although such integrated-care programmes are designed to meet the elderly's needs, previous studies show mixed (cost-)effectiveness results.[8–10] A potential explanation is that common methods to assess effectiveness tend to focus on measuring traditional outcomes, such as physical functioning, whereas that is not the primary focus of these programmes.[8,11] Cost-effectiveness analyses measure Quality-Adjusted Life-Year (QALY) gains, which is also less appropriate because integrated care for frail elderly focuses more on well-being than on survival and health-related quality of life.[12] For example, elderly are stimulated to visit outpatient day-care activities to enhance their social participation, or their experience with care is improved by individual care planning and helping them navigate through the health- and social-care system. Accordingly, empirical evaluations should include these domains to accurately value the potential benefits of an intervention.[13,14]

Therefore, we adopted a broader evaluation method, namely Multi-Criteria Decision Analysis (MCDA) to evaluate the CCFE. MCDA is innovative in elderly care because it synthesises a wide variety of outcome measures, in this case patient-reported health and well-being measures (PROMS), experience with care measures (PREMS), and costs. Together, they cover the Triple Aim.[15] In the MCDA outcomes are weighted by their importance according to five stakeholder groups: patients, informal caregivers, professionals, policymakers, and payers. The weighted performance scores are aggregated into overall value scores.[16]

For peer review only

METHODS

Study population

The study population consisted of community-dwelling frail elderly, where frailty is defined as loss of functional abilities and control over one's life and in need of complex care. To participate in the intervention, they had to be registered at a GP-practice from one of three care groups (i.e., an association of primary care providers that cooperate in the provision of chronic care) that offer the CCFE. They had to be able to comprehend study information and answer questions, either independently or with the help of an informal caregiver or trained interviewer.

Intervention

A primary care team consisting of the GP, nurse-practitioner, and district nurse, identifies potential candidates for the CCFE. The nurse-practitioner visits the elderly at home and performs a comprehensive geriatric assessment of the needs, capabilities, and preferences in the physical, psychological, cognitive, and social domains. Depending on this assessment, the nurse-practitioner consults professionals in the community (e.g., physical therapists, occupational therapists, social workers, elderly care physicians, geriatricians, dementia case workers) and arranges informal care support. Together with the frail elderly and informal caregiver, the nurse-practitioner drafts an individual care plan, largely driven by the elderly's personal goals.

The nurse-practitioner organises multidisciplinary team meetings to discuss the individual care plan with all professionals involved. The frail elderly and their informal caregiver participate in these meetings. During the first meeting a case manager is assigned, a role mostly taken up by the nurse-practitioner. The case manager is the main contact point, monitors the execution of the individual care plan, and further adapts the care to the patient's wishes and additional needs. Once enrolled, an elderly person usually stays in the CCFE until nursing home admission or if they pass away.

For peer review only

131 To support collaboration between professionals, the CCFE uses 'Care2U', a secured ICT-platform to share information. Professionals have different degrees of access and the elderly must approve access. Care2U includes the individual care plan and is used by the nurse-practitioner to monitor appointments and services of providers in the care chain.

135 The CCFE is financed by a bundled payment contract between each care group and the dominant health insurer in the region and is re-negotiated every year.

137 *Study design*

138 The MCDA was conducted as part of a 12-month prospective quasi-experimental study comparing two parallel groups:

140 *Intervention group* included frail elderly enrolled into the CCFE between April 2017-August 2018 and were recruited to participate in the study by either their GP or nurse-practitioner.

142 *Control group* consisted of frail elderly receiving usual care, recruited at GP-practices from one of three participating care groups. These practices had not (yet) implemented the CCFE. To ensure a similar level of frailty in both groups, the GP-practices in the control group applied the same case-finding approach. They were assisted by a GP specialised in elderly care that had experience with the CCFE.

146 Self-reported outcome and experience measures and care utilization data were gathered at baseline and after 6 months and 12 months, during face-to-face interviews at the elderly's home, performed by trained interviewers. The interviewers were not involved in delivering any aspects of the intervention but were aware of which group the respondent belonged to. Data collection took place between April 2017-August 2019.

151 *Outcome measures*

For peer review only

Table 1 gives an overview of all outcome measures. These outcomes were selected based on a literature review, workshops with representatives from the five stakeholder groups, and focus groups with individuals with multi-morbidity, and measured with validated questionnaires.[17]

Table 1. Outcome measures and instruments

Core set^	Instrument to measure outcome	Scale
**Health & Well-being**		
Physical functioning	Activities of Daily Living (ADL) (Katz-15)[18]	0-15 (worst)
Psychological well-being	Mental Health Inventory (MHI-5)[19]	0-100 (best)
Enjoyment of life	Investigating Choice Experiments for the Preferences of Older People (ICECAP-O)[20]	1-4 (best)
Social relationships & participation	Impact on Participation & Autonomy (IPA), social life and relationships domain[21]	0-28 (worst)
Resilience	Brief Resilience Scale (BRS)[22]	6-30 (best)
**Experience of care**		
Person-centeredness	Person-centered Coordinated Care Experience Questionnaire (P3CEQ), experience of person-centered care domain[23]	0-18 (best)
Continuity of care	Nijmegen Continuity Questionnaire (NCQ), team and cross boundary continuity domain[24] + Client Perceptions of Coordination Questionnaire (CPCQ)[25]	1-5 (best)
**Costs**		
Health, social, and informal care costs	iMTA Medical Consumption Questionnaire[26]	
Medication costs	Prescriptions in patient records extracted from GP information systems	
Bundled payments and chronic care programmes	Care chain information system 'Care2U'	
**Additional outcomes**		
Autonomy	Pearlin Mastery Scale[27]	7-35 (best)
Burden of medication	Living with Medicines Questionnaire - LMQ[28]	0-10 (worst)

^The core set of outcomes was measured across all studies included in the SELFIE project. For these outcomes, weights were elicited, and these outcomes were included in the MCDA. The additional outcomes were not included in the MCDA.

For peer review only

For peer review only - http://bmjopen.bmj.com/site/about/guidelines.xhtml

Costs

Health- and social-care utilization data were obtained with the iMTA-Medical Consumption Questionnaire, which includes questions about contacts with GP, nurse-practitioner, GP-assistants, physiotherapists and other paramedical therapists, dieticians, psychologists, social workers, welfare workers, and medical specialists, hospital admissions, rehabilitation, homecare, residential care and nursing homes, and informal care during the past three months.[26] Unit costs were largely based on reference prices from the Dutch Costing Manual.[29] Medication costs were based on prescription data from GP-information systems, which were combined with unit costs from Dutch drug database '*G-Standaard*', using ATC-codes.[30] Programme costs of the CCFE were based on the bundled payment contracts between each care group and the dominant health insurer.[1] Elderly from the control group could participate in single-disease care programmes for diabetes, cardiovascular risk management or Chronic Obstructive Pulmonary Disease. The proportion of elderly participating in these programmes was obtained from Care2U and average prices of the bundled payments were obtained from the Dutch health claims database (Vektis).[31]

Statistical analysis

Comparability of groups

We applied inverse probability weighting to increase the comparability of the intervention and control groups at baseline.[32] The logistic regression model to estimate the propensity score included age, gender, marital status, living situation, educational level, smoking status, and costs three months prior to baseline as a proxy for complexity. The propensity score p reflects the estimated probability of an individual to be in the intervention group. By setting the weight for individuals in the intervention group to 1, and for individuals in the control group to $p/(1-p)$, we estimated the average treatment effect in the treated (ATT) in the weighted mixed effect models described below.[33] To assess the comparability of the two groups, we calculated the mean percentage standardised bias, the Rubin's B (absolute standardised

For peer review only

183 difference of the means of the linear index of the propensity score in the intervention group and matched controls), and the Rubin's R (ratio of intervention group and matched control group variances of the propensity score index). For sufficient balance, B should be less than 25% and R be between 0.5 and 2.[34]

187 Treatment effects

188 Treatment effects were estimated using weighted mixed effect models with a random intercept at individual level (as we had longitudinal data) and the following covariates: time, intervention, an interaction term for time and intervention, age, gender, marital status, living situation, educational level, and smoking status. This combination of matching and regression adjustment has been shown to best reduce covariate imbalance between groups.[35] The mixed effects models were used to predict the mean scores of the outcome measures in both groups at each time point, assuming the control group had the same baseline score as the intervention group to directly compare both groups. All statistical analyses were performed using STATA16.1.

196 MCDA

197 In the MCDA, the predicted mean scores of the outcomes at 6- and 12-months follow-up were standardised on a 0-1 scale to remove differences in measurement scales, using relative standardization, see **Appendix 1**. For all outcomes in the MCDA, a higher score indicates better performance. The standardised outcomes were weighted by their importance and subsequently summed to obtain an overall value score for the intervention and control group separately. The relative importance-weights were elicited in an online weight elicitation study among patients, informal caregivers, professionals, payers, and policymakers, using a discrete choice experiment (DCE).[36] The relative weights of the outcome measures included in the MCDA by stakeholder group can be found in **Appendix 2**.

205

For peer review only

Sensitivity analysis

To assess the joint uncertainty in outcome scores and importance-weights, we performed a probabilistic sensitivity analysis using Monte Carlo simulation. We used Cholesky decomposition to obtain 10,000 replications of both the standardised outcomes and the importance-weights. From this, we obtained the 95%-confidence intervals around the overall value scores for each stakeholder group. Additionally, we calculated the proportion of MCDA-iterations in which the CCFE has a higher overall value score than usual care.

Patient and Public Involvement

Patients were involved in the selection of outcome measures, which was largely based on focus groups as described above.[17] In multiple National Stakeholder Workshops held during the entire process of the study, stakeholders from the five stakeholder groups were asked to reflect upon the outcome measures, the study design and data collection, and the results of the study. The study design was set-up in close collaboration with care providers to ensure feasibility of the data collection. Before the start of data collection, the questionnaire was piloted in a frail older person. The authors disseminated results via conference presentations. Results of this study were also disseminated to participating care providers, patients and informal caregivers using communication methods other than scientific papers, i.e., by e-mail and newsletters disseminated at the GP-practices.

Ethics approval and consent to participate

The study was conducted as one of the case studies in Horizon2020-project SELFIE (Sustainable intEgrated care modeLs for multi-morbidity: delivery, FInancing and performancE).[37] The study protocol was reviewed by the medical ethics committee of the Erasmus Medical Centre, the Netherlands. The committee concluded that the rules laid down in the Dutch Medical Research Involving Human Subjects

For peer review only

228 Act do not apply to this research (*MEC-2017-121*). All study participants have provided informed written

229 consent.

For peer review only

RESULTS

Respondents

Figure 1 presents the flow chart of patients included in the study. The retention rate at 12-month follow-up was 70% in both groups. Main reasons of loss-to-follow-up were also similar in both groups and included death, burden of study participation, and cognitive incapacity.

*****Figure 1 here*****

Table 2 presents the baseline characteristics of respondents before and after inverse probability weighting. After IPW, the matching statistics were within the desired range. No substantial differences between the groups remained. The common support graph shows a good overlap in propensity scores, see **Appendix 3**.

For peer review only

Table 2. Baseline characteristics before and after inversed probability weighting

	CCFE N=222	UC N=162	St. diff.	UC N=162	St. diff.
**Baseline characteristics**	**Before IPW**			**After IPW**	
Age, mean	83.4	84.8	-0.224	83.6	-0.028
Female (%)	63.5	64.2	-0.014	64.8	-0.027
Marital status (%)					
Single, never married	3.6	3.7	-0.005	2.6	0.054
Married or living together	44.6	40.7	0.078	45.0	-0.008
Widow(er)	44.6	50.6	-0.120	47.1	-0.049
Divorced	7.2	4.9	0.095	5.4	0.078
Living situation (%)					
Independent, alone	54.1	61.1	-0.143	55.2	-0.024
With others	46.0	38.9	0.143	44.8	0.024
Educational level (%)					
Low	70.3	70.4	-0.002	73.3	-0.067
Medium	20.3	14.8	0.143	14.3	0.158
High	9.5	14.8	-0.164	12.4	-0.090
Current smoker (%)	14.4	8.6	0.181	13.8	0.019
**Outcome measures at baseline, mean**	**Before IPW**			**After IPW**	
Physical functioning (0-15) ^	4.4	4.5	-0.032	4.5	-0.052
Psychological well-being (0-100)	71.4	71.7	-0.018	70.6	0.040
Enjoyment of life (1-4)	2.8	2.9	-0.137	2.9	-0.143
Social relationships and participation (0-28) ^	9.2	8.0	0.345	8.2	0.280
Resilience (6-30)	19.3	19.0	0.062	19.0	0.082
Autonomy (7-35)	22.3	22.1	0.054	21.9	0.098
Burden of medication (0-10) ^	2.1	2.4	-0.111	2.5	-0.166
Person-centeredness (0-18)	11.7	12.5	-0.209	12.7	-0.246
Continuity of care (1-5)	3.7	3.8	-0.140	3.8	-0.182
Total costs 3 months prior to the study (€) ^	5,453	5,267	0.028	5,631	-0.026
**Statistics to assess matching[#]**	**Before IPW**			**After IPW**	
Mean bias			11.9		5.1
Rubin's B			39.7		21.4
Rubin's R			1.06		1.10

^ Higher score indicating worse performance; St. diff = Absolute Standardised Mean Difference, also called Absolute Standardised Bias; # On variables used in PSM, i.e., age, gender, marital status, living situation, educational level, smoking, total costs 3 months prior to the study. CCFE=Care Chain Frail Elderly. UC=Usual care. IPW=Inverse Probability Weighting

For peer review only

247 *Treatment effects*

248 **Table 3** reports the estimated treatment effects of the CCFE at 6- and 12-months of follow-up. Results
249 show that the CCFE improved person-centeredness at both time points. At 6 months follow-up, physical
250 functioning declined in both groups but even further in the intervention group. At 12 months follow-up,
251 the CCFE performed worse on autonomy and burden of medication. The predicted mean performance
252 scores of all outcomes on their natural scale can be found in **Appendix 4**.

For peer review only

For peer review only - http://bmjopen.bmj.com/site/about/guidelines.xhtml

253 **Table 3.** Treatment effects at 6- and 12-months follow-up

	**6 months**			**12 months**		
**Core set of outcomes included in the MCDA**	**Estimated change CCFE**	**Estimated Change UC**	**Diff in change Mean (95% CI)#**	**Estimated change CCFE**	**Estimated change UC**	**Diff in change Mean (95% CI)#**
**Health & Well-being**						
Physical functioning (0-15) ^	0.74	0.27	0.47 (0.06 ; 0.88)*	1.33	0.95	0.39 (-0.10 ; 0.87)
Psychological well-being (0-100)	-0.64	-0.34	-0.30 (-3.86 ; 3.25)	-1.35	-0.10	-1.26 (-5.20 ; 2.68)
Enjoyment of life (1-4)	0.10	-0.08	0.18 (-0.02 ; 0.38)	0.05	0.02	0.04 (-0.18 ; 0.25)
Social relationships & participation (0-28) ^	0.11	0.41	-0.31 (-1.04 ; 0.43)	0.26	0.61	-0.36 (-1.25 ; 0.54)
Resilience (6-30)	-0.02	-0.21	0.19 (-0.63 ; 1.01)	0.03	0.11	-0.08 (-1.01 ; 0.85)
**Experience of care**						
Person-centeredness (0-18)	1.10	-0.38	1.48 (0.57 ; 2.38)*	1.33	0.00	1.33 (0.18 ; 2.49)*
Continuity of care (1-5)	0.10	-0.02	0.12 (-0.05 ; 0.28)	0.16	0.03	0.13 (-0.05 ; 0.31)
**Costs**						
Total health and social care costs^	5,405	4,745	660 (-1,650 ; 2,970)	17,223	15,206	2,017 (-2,361 ; 6,395)
**Additional frail elderly-specific outcomes**	**Estimated change CCFE**	**Estimated change UC**	**Diff in change Mean (95% CI)#**	**Estimated change CCFE**	**Estimated change UC**	**Diff in change Mean (95% CI)#**
Autonomy (7-35)	-0.50	0.02	-0.52 (-1.38 ; 0.34)	-0.49	0.55	-1.04 (-1.95 ; -0.14)*
Burden of medication (0-10) ^	0.13	-0.21	0.34 (-0.36 ; 1.04)	0.18	-0.61	0.78 (0.04 ; 1.53)*

254 *^ Higher score indicating worse performance; # based on robust SE; * p<0.05. MCDA=Multi-Criteria Decision Analysis. CCFE=Care Chain Frail Elderly. UC=Usual care.*
255 *CI=Confidence interval.*

For peer review only - http://bmjopen.bmj.com/site/about/guidelines.xhtml

Costs

Table 4 presents details on the mean costs after inversed probability weighting at 6- and 12-months, from a healthcare and a societal perspective. From a healthcare perspective, after 6 months, costs were €751 higher in the intervention group than in the control group. After 12 months, the costs were €1,796 higher. These differences were largely due to programme costs and costs of homecare.

When adopting a societal perspective, 6-month costs were €662 higher in the intervention group than in the control group, whereas 12-month costs were €2,136 higher. Costs of informal care were slightly lower in the intervention group than in the control group after 6 months, but higher after 12 months.

For peer review only

For peer review only - http://bmjopen.bmj.com/site/about/guidelines.xhtml

Table 4. Mean costs (€) after 6- and 12-months of follow-up (after IPW)

	6 months			12 months		
**Cost category**	**Mean costs (SD) CCFE (n=172)**	**Mean costs (SD) UC (n=129)**	**Difference between means (SE)**	**Mean costs (SD) CCFE (n=156)**	**Mean costs (SD) UC (n=113)**	**Difference between means (SE)**
Chronic care programme(s) #	534 (-)	72 (-)	462 (-)	1,068 (-)	143 (-)	925 (-)
Homecare	3,289 (4,371)	3,158 (4,016)	131 (481)	7,597 (8474)	6,330 (7680)	1,267 (980)
Long-term care admissions	969 (5,411)	766 (4,980)	203 (589)	1,624 (7,959)	2,051 (10,025)	-427 (11,33)
Hospital admissions	670 (2,670)	485 (1,833)	185 (259)	1,199 (3,603)	1,119 (2,992)	80 (403)
Emergency room visits	112 (308)	116 (341)	-4 (38)	179 (372)	196 (418)	-17 (49)
Outpatient day-care activities	235 (1,180)	310 (1,481)	-74 (170)	627 (2,642)	408 (1,846)	218 (285)
Medical specialist care	338 (619)	317 (408)	21 (60)	631 (789)	609 (615)	23 (86)
Paramedical care (e.g., physiotherapist)	529 (636)	481 (548)	48 (72)	1,059 (1,113)	1,005 (1,133)	55 (145)
General practitioner ~	21 (27)	288 (272)	-267 (28)	25 (34)	492 (394)	-467 (44)
Medication	363 (662)	318 (551)	45 (65)	744 (1,232)	605 (927)	139 (124)
**Subtotal costs from healthcare perspective**	**7 060 (8,441)**	**6,310 (7,671)**	**751 (922)**	**14,753 (14,498)**	**12,957 (14,185)**	**1,796 (1,777)**
Informal care	3,690 (5,780)	3,779 (7,318)	-89 (891)	8,063 (12,436)	7,723 (13,643)	340 (1,783)
**Total costs from societal perspective**	**10,750 (10,381)**	**10,089 (11,003)**	**662 (1,309)**	**22,816 (19,050)**	**20,680 (20,251)**	**2,136 (2,551)**

# Costs for chronic care program(s) is an average estimation which is the same for each respondent in their respective group; ~ Costs for GP-care in the intervention group are largely included in the costs of the chronic care program. SD=Standard deviation. SE=Standard Error. CCFE=Care Chain Frail Elderly. UC=Usual care.

For peer review only - http://bmjopen.bmj.com/site/about/guidelines.xhtml

Multi-Criteria Decision Analysis

Table 5a and b present the standardised outcome scores for the CCFE and usual care as well as these scores weighted according to each stakeholder's importance-weights, at 6- and 12-months, respectively. The overall value scores show that all stakeholder groups preferred the CCFE over usual care at 6 months follow-up. This was driven by the performance scores of person-centeredness and enjoyment of life, and the high importance-weight of the latter. In more than 75% of iterations the CCFE had a higher overall value score than usual care. At 12 months follow-up the difference disappeared, and the probability that the CCFE had a higher overall value score dropped below 50% for payers and policymakers. This was driven by worse scores in physical functioning and costs.

For peer review only

For peer review only - http://bmjopen.bmj.com/site/about/guidelines.xhtml

276 **Table 5a.** Value scores in the Multi-Criteria Decision Analysis at 6 months, using DCE weights

			Patients		Partners		Professionals		Payers		Policymakers	
**Outcome measures**	**Standardised performance score#**		**Weighted score**		**Weighted score**		**Weighted score**		**Weighted score**		**Weighted score**	
	**CCFE**	**UC**	**CCFE**	**UC**	**CCFE**	**UC**	**CCFE**	**UC**	**CCFE**	**UC**	**CCFE**	**UC**
Physical functioning	0.672	0.740	0.108	0.118	0.074	0.081	0.081	0.089	0.094	0.104	0.094	0.104
Psychological well-being	0.706	0.709	0.120	0.120	0.106	0.106	0.127	0.128	0.127	0.128	0.106	0.106
Enjoyment of life	0.729	0.685	0.168	0.157	0.182	0.171	0.160	0.151	0.175	0.164	0.160	0.151
Social relationships and participation	0.718	0.696	0.057	0.056	0.065	0.063	0.079	0.077	0.072	0.070	0.072	0.070
Resilience	0.711	0.704	0.107	0.106	0.099	0.099	0.092	0.091	0.078	0.077	0.099	0.099
Person-centeredness	0.749	0.663	0.060	0.053	0.060	0.053	0.060	0.053	0.045	0.040	0.060	0.053
Continuity of care	0.718	0.696	0.072	0.070	0.086	0.083	0.079	0.077	0.057	0.056	0.072	0.070
Costs (societal care perspective)	0.685	0.729	0.021	0.022	0.041	0.044	0.041	0.044	0.055	0.058	0.048	0.051
**Overall value scores**			**0.711**	**0.702**	**0.713**	**0.700**	**0.719**	**0.708**	**0.703**	**0.696**	**0.711**	**0.702**
**95% Confidence interval^**			0.702 - 0.721	0.692 - 0.712	0.703 - 0.723	0.690 - 0.710	0.702 - 0.722	0.691 - 0.711	0.699 - 0.721	0.692 - 0.714	0.700 - 0.721	0.692 - 0.712
**% CCFE > UC^**			82%		90%		87%		75%		81%	

277

For peer review only - http://bmjopen.bmj.com/site/about/guidelines.xhtml

Table 5b. Value scores in the Multi-Criteria Decision Analysis at 12 months, using DCE weights

			Patients		Partners		Professionals		Payers		Policymakers	
**Outcome measures**	**Standardised performance score[#]**		**Weighted score**		**Weighted score**		**Weighted score**		**Weighted score**		**Weighted score**	
	**CCFE**	**UC**	**CCFE**	**UC**	**CCFE**	**UC**	**CCFE**	**UC**	**CCFE**	**UC**	**CCFE**	**UC**
Physical functioning	0.682	0.731	0.109	0.117	0.075	0.080	0.082	0.088	0.095	0.102	0.095	0.102
Psychological well-being	0.701	0.713	0.119	0.121	0.105	0.107	0.126	0.128	0.126	0.128	0.105	0.107
Enjoyment of life	0.711	0.703	0.164	0.162	0.178	0.176	0.157	0.155	0.171	0.169	0.157	0.155
Social relationships and participation	0.720	0.694	0.058	0.056	0.065	0.062	0.079	0.076	0.072	0.069	0.072	0.069
Resilience	0.706	0.709	0.106	0.106	0.099	0.099	0.092	0.092	0.078	0.078	0.099	0.099
Person-centeredness	0.744	0.668	0.060	0.053	0.060	0.053	0.060	0.053	0.045	0.040	0.060	0.053
Continuity of care	0.719	0.695	0.072	0.069	0.086	0.083	0.079	0.076	0.058	0.056	0.072	0.069
Costs (societal care perspective)	0.673	0.739	0.020	0.022	0.040	0.044	0.040	0.044	0.054	0.059	0.047	0.052
**Overall value scores**			**0.707**	**0.707**	**0.708**	**0.706**	**0.714**	**0.713**	**0.698**	**0.702**	**0.706**	**0.707**
**95% Confidence interval^**			0.696 - 0.717	0.697 - 0.717	0.697 - 0.718	0.695 - 0.716	0.697 - 0.717	0.695 - 0.716	0.695 - 0.717	0.697 - 0.719	0.695 - 0.717	0.696 - 0.718
**% CCFE > UC^**			50%		57%		54%		39%		48%	

^ based on Monte Carlo simulation; [#] colour scheme ranges from red (lowest score) to green (highest score). CCFE=Care Chain Frail Elderly. UC=Usual care.

For peer review only - http://bmjopen.bmj.com/site/about/guidelines.xhtml

DISCUSSION

Main findings

The MCDA has shown that all stakeholders preferred the CCFE over usual care at 6 months with a likelihood of having a higher overall value score of over 75%. This was mainly driven by higher performance on enjoyment of life and person-centeredness, and the great importance of the former outcome. Results became more diffuse at 12 months. Patients were indifferent, informal caregivers, and professionals slightly favoured the CCFE, whereas payers and policymakers demonstrated a slight preference for usual care. This was mainly due to a worse performance of the CCFE on physical functioning and costs. When looking at the disaggregated scores, person-centeredness was consistently higher in the CCFE. Physical functioning deteriorated in the intervention group at 6 months, but this effect disappeared at 12 months. When some outcomes improve whereas others deteriorate, the current MCDA approach is a suitable method to aggregate them into overall value scores that vary depending on the importance that stakeholders assign to the different outcome measures. These results show that the CCFE is the preferred way of delivering care to frail elderly if improvements in enjoyment of life and person-centeredness are considered more important than physical functioning and costs.

Comparison with previous evaluations

As this is the first MCDA of a frail elderly program, it is impossible to directly compare the value scores to other studies. MCDA also provides insight in the disaggregated effects of the CCFE, but these are hard to compare to other studies as well, due to the very frail target group of the CCFE, the different contexts in which the interventions are implemented, the different intervention components, and outcome measures[38,39] Nevertheless, most previous studies did not find effects on physical functioning.[8,40] This may be expected as these programmes rarely aim to achieve

For peer review only - http://bmjopen.bmj.com/site/about/guidelines.xhtml

improvements in this domain. As the CCFE aimed to improve experience with care, the sustained improvement in person-centeredness found in this study suggests that the programme has fulfilled that aim.[1] The worsening in autonomy and burden of medication seems counterintuitive. A possible explanation for the deterioration in autonomy is that as elderly in the CCFE were confronted with their frailty, e.g., by discussing their needs for support, they became more aware of their loss of control. This could have led to lower autonomy scores in the intervention group, especially due to the self-report method; measurement by a third party might have led to different scores.[41] The higher medication burden in the intervention group could be explained by alterations in medication after the medication review, which may have led to (temporary) side-effects.

Strengths & limitations

One of the strengths of our study was its controlled study design. Defining an appropriate control group to evaluate ongoing programmes for frail elderly is a challenge.[8,42] However, it is especially important in evaluating frail elderly care, as there is commonly little room for actual improvements in health, and prevention or delay of deterioration can only be shown in comparison with a control group.

Another strength was the data collection on a broad range of PROMs and PREMs by interviewers who made a total of 954 home-visits to the elderly, which was a major endeavour. Collecting patient-reported data did limit the generalisability of the results, as some frail elderly could not participate in the evaluation because that was too burdensome or impossible. Hence, the frailest among the elderly were not represented in this study, especially not those with dementia. The attrition rate in our study was relatively low, i.e., 30% across both groups at 12 months. We did observe that elderly in the control group that were lost to follow-up were slightly older and had worse physical functioning at baseline compared to non-dropouts in the control group (and overall), see **Appendix 5**. This may have led to an underestimation of the treatment effect.

For peer review only - http://bmjopen.bmj.com/site/about/guidelines.xhtml

A further strength of our study was the detailed cost-analysis, even including costs of medication, social care and informal care, which are often excluded from other studies.[8] This analysis showed a cost increase reflecting the greater investment of resources to support frail elderly in ageing in place which is of great importance to many elderlies.

In the design and reporting on the MCDA we followed the good practice guidelines as laid out by the ISPOR MCDA-taskforce.[43] Strengths of MCDA are that it enables explicit, transparent and accountable decision-making, i.e., for every decision what was valued most and by whom can be tracked down as well as whether this was due to improvements in certain domains or a higher relative importance of a particular outcome. Furthermore, MCDA makes it possible to include additional elements of value that go beyond health or QALYs, which is especially important for complex interventions with multiple aims such as improving well-being and experience with care. However, the consequence is that we may favour interventions that achieve improvements in these outcomes above interventions that have greater health outcomes. This may be justifiable for elderly care. Such an argument would raise another point that is debated in MCDA, namely whether or not to include costs in the overall value score.[44,45] To elicit a weight for costs, stakeholders had to trade-off costs against other outcomes, which makes the relative contribution of costs to the overall value score explicit. However, it can be argued that this does not adequately address the opportunity costs of the CCFE.[46] We also performed the MCDA without costs as a sensitivity analysis, see **Appendix 6**, which led to higher overall value scores for the CCFE at both time points. However, now the overall value forms a composite benefit score for which a new cost-effectiveness threshold must be determined. Although we believe the current set of outcomes captures the full potential value of an integrated care programme for frail elderly, this set should be tailored to each intervention's aims and target group. Therefore, when the set of outcome measures changes, new thresholds need to be determined. Another option is to calculate the cost-per-value and prioritise interventions with the lowest cost-per-value ratio, but this only leads to a ranking of interventions.[45] A last point of discussion on MCDA is that it requires a deliberative component to avoid making decisions based

For peer review only - http://bmjopen.bmj.com/site/about/guidelines.xhtml

solely on the model.[45] In our study we presented results from five stakeholder perspectives which inevitably calls for further deliberation to determine which perspective should prevail.

Implications

Although the CCFE does not improve the (physical) health of patients, it is still positively evaluated by all stakeholder groups at 6 months. At 12 months stakeholders were mainly indifferent. This warrants further research into interventions maintaining the effects of such programmes in the long-term. Furthermore, we advocate a wider use of MCDA to evaluate multi-faceted, person-centred, integrated care programmes for frail elderly that aim to improve multiple outcomes, including those that go beyond health. MCDA enables a transparent and explicit decision-making process and serves as a tool to help decision-makers reach a decision. Therefore, MCDA-results are a good starting point for deliberation before deciding upon reimbursement or broader implementation of new interventions.

CONCLUSION

After 6 months, the overall value score for the CCFE was higher than for usual care across all stakeholders, but at 12 months, the preference for the CCFE had disappeared. The CCFE led to sustained improvements in enjoyment of life and person-centeredness, which is aligned with the program's aim, but also to a deterioration in physical functioning at 6 months and higher costs. Therefore, the CCFE is only the preferred way of delivering care to frail elderly in case improvements in enjoyment of life and person-centeredness are considered more important than costs and physical functioning.

For peer review only - http://bmjopen.bmj.com/site/about/guidelines.xhtml

ACKNOWLEDGEMENTS

We would like to thank all respondents and their informal caregivers for participating in this research. We would also like to thank the care groups DOH, SGE and PoZoB for their continuous support in facilitating this research.

AUTHOR CONTRIBUTIONS

MH, MK, FL, AT, MRvM developed the overall methodology for the value-based MCDA approach in the SELFIE project. MH, MK, FL, MRvM contributed to the study design and data collection. MH, MK, LG, KI, AT, MRvM had access to the data and contributed to the data analysis. MH and MRvM drafted this manuscript. All co-authors critically reviewed this manuscript. All authors read and approved the final manuscript. MH and MRvM had full access to all of the data in the study and the final responsibility to submit for publication.

DECLARATION OF INTERESTS

The authors have no competing interests.

FUNDING

The SELFIE project has received funding from the European Union’s Horizon 2020 research and innovation programme under grant agreement No 634288. The content of this manuscript reflects only the SELFIE group’s views and the European Commission is not liable for any use that may be made of the information contained herein.

DATA SHARING AGREEMENT

The data that support the findings of this study are available on request from the corresponding author, MH. The data are not publicly available due to privacy restrictions.

For peer review only

REFERENCES

1. Hoedemakers M, Leijten FRM, Looman W, Czypionka T, Kraus M, Donkers H, et al. Integrated Care for Frail Elderly: A Qualitative Study of a Promising Approach in The Netherlands. International Journal of Integrated Care. 2019 Sep 3;19(3):16.
2. Maarse JAM, Jeurissen PP. The policy and politics of the 2015 long-term care reform in the Netherlands. Health Policy. 2016 Mar 1;120(3):241–5.
3. Alders P, Schut FT. The 2015 long-term care reform in the Netherlands: Getting the financial incentives right? Health Policy. 2019 Mar 1;123(3):312–6.
4. Wehrens R, Oldenhof L, Verweij L, Francke A, Bal R. Experimenteel sturen in netwerken: een evaluatie van proces en structuur van het Nationaal Programma Ouderenzorg. :99.
5. de Jong B, Wynia K, Geluk-Bleumink A. Ageing Better in the Netherlands. In D'Onofrio G, Sancarlo D, Greco A, editors, Gerontology. London: IntechOpen. 2018. p. 101-111
6. Briggs AM, Valentijn PP, Thiyagarajan JA, Carvalho IA de. Elements of integrated care approaches for older people: a review of reviews. BMJ Open. 2018 Apr 1;8(4):e021194.
7. Hendry A, Vanhecke E, Carriazo A, López-Samaniego L, Espinosa J, Sezgin D, et al. Integrated Care Models for Managing and Preventing Frailty: A Systematic Review for the European Joint Action on Frailty Prevention (ADVANTAGE JA). Transl Med UniSa. 2019 Jan 6;19:5–10.
8. Looman WM, Huijsman R, Fabbricotti IN. The (cost-)effectiveness of preventive, integrated care for community-dwelling frail older people: A systematic review. Health & Social Care in the Community. 2019;27(1):1–30.
9. Van der Elst M, Schoenmakers B, Duppen D, Lambotte D, Fret B, Vaes B, et al. Interventions for frail community-dwelling older adults have no significant effect on adverse outcomes: a systematic review and meta-analysis. BMC Geriatrics. 2018 Oct 20;18(1):249.

For peer review only

For peer review only - http://bmjopen.bmj.com/site/about/guidelines.xhtml

10. Hopman P, de Bruin SR, Forjaz MJ, Rodriguez-Blazquez C, Tonnara G, Lemmens LC, et al. Effectiveness of comprehensive care programmes for patients with multiple chronic conditions or frailty: A systematic literature review. Health Policy. 2016 Jul 1;120(7):818–32.
11. Vorst A van der, Zijlstra GAR, Witte ND, Duppen D, Stuck AE, Kempen GIJM, et al. Limitations in Activities of Daily Living in Community-Dwelling People Aged 75 and Over: A Systematic Literature Review of Risk and Protective Factors. PLOS ONE. 2016 Oct 19;11(10):e0165127.
12. Makai P. Moving beyond the QALY: economic evaluation in health and social care for elderly populations. [Rotterdam]: Erasmus Universiteit Rotterdam; 2014.
13. Cramm J, Nieboer A. Is ”disease management” the answer to our problems? No! Population health management and (disease) prevention require ”management of overall well-being” BMC Health Services Research. 2016 Sep 21;16.
14. Makai P, Brouwer WBF, Koopmanschap MA, Stolk EA, Nieboer AP. Quality of life instruments for economic evaluations in health and social care for older people: A systematic review. Social Science & Medicine. 2014 Feb 1;102:83–93.
15. Berwick DM, Nolan TW, Whittington J. The Triple Aim: Care, Health, And Cost. Health Affairs. 2008 May 1;27(3):759–69.
16. Rutten-van Mölken M, Leijten F, Hoedemakers M, Tsiachristas A, Verbeek N, Karimi M, et al. Strengthening the evidence-base of integrated care for people with multi-morbidity in Europe using Multi-Criteria Decision Analysis (MCDA). BMC Health Services Research. 2018 Jul 24;18(1):576.
17. Leijten FRM, Hoedemakers M, Struckmann V, Kraus M, Cheraghi-Sohi S, Zemplényi A, et al. Defining good health and care from the perspective of persons with multimorbidity: results from a qualitative study of focus groups in eight European countries. BMJ Open. 2018 Aug 1;8(8):e021072.

For peer review only - http://bmjopen.bmj.com/site/about/guidelines.xhtml

18. Laan W, Zuithoff NPA, Drubbel I, Bleijenberg N, Numans ME, De Wit NJ, et al. Validity and reliability of the Katz-15 scale to measure unfavorable health outcomes in community-dwelling older people. J Nutr Health Aging. 2014 Nov 1;18(9):848–54.

19. Rumpf H-J, Meyer C, Hapke U, John U. Screening for mental health: validity of the MHI-5 using DSM-IV Axis I psychiatric disorders as gold standard. Psychiatry Research. 2001 Dec 31;105(3):243–53.

20. Coast J, Peters TJ, Natarajan L, Sproston K, Flynn T. An assessment of the construct validity of the descriptive system for the ICECAP capability measure for older people. Qual Life Res. 2008; 17(7): 967-76.

21. Cardol M, de Haan RJ, van den Bos GA, de Jong BA, de Groot IJ. The development of a handicap assessment questionnaire: the Impact on Participation and Autonomy (IPA). Clin Rehabil. 1999; 13(5): 411-9.

22. Smith BW, Dalen J, Wiggins K, Tooley E, Christopher P, Bernard J. The brief resilience scale: assessing the ability to bounce back. Int J Behav Med. 2008; 15(3): 194-200.

23. Lloyd H, Fosh B, Whalley B, Byng R, Close J. Validation of the person-centred coordinated care experience questionnaire (P3CEQ). International Journal for Quality in Health Care. 2019 Aug 1;31(7):506–12.

24. Uijen AA, Schellevis FG, van den Bosch WJ, Mokkink HG, van Weel C, Schers HJ. Nijmegen Continuity Questionnaire: development and testing of a questionnaire that measures continuity of care. J Clin Epidemiol. 2011; 64(12): 1391-9.

25. McGuiness C, Sibthorpe B. Development and initial validation of a measure of coordination of health care. Int J Qual Health Care. 2003; 15(4): 309-18.

26. Bouwmans C, Krol M, Severens H, Koopmanschap M, Brouwer W, Roijen LH. The iMTA Productivity Cost Questionnaire: A Standardised Instrument for Measuring and Valuing Health-Related Productivity Losses. Value in Health. 2015 Sep 1;18(6):753–8.

For peer review only - http://bmjopen.bmj.com/site/about/guidelines.xhtml

27. Pearlin LI, Schooler C. The Structure of Coping. Journal of Health and Social Behavior. 1978;19(1):2–21.

28. Krska J, Morecroft CW, Rowe PH, Poole H. Measuring the impact of long-term medicines use from the patient perspective. Int J Clin Pharm. 2014 Aug 1;36(4):675–8.

29. Kanters TA, Bouwmans CAM, Linden N van der, Tan SS, Roijen LH. Update of the Dutch manual for costing studies in health care. PLoS ONE. 2017 Nov 9;12(11):e0187477.

30. G-Standaard Flex — Z-Index [Internet]. [cited 2021 Feb 2]. Available from: https://www.z-index.nl/producten-diensten/g-standaard-flex.

31. Vektis - business intelligence centrum voor de zorg | Vektis.nl [Internet]. Vektis. [cited 2021 Feb 2]. Available from: https://www.vektis.nl.

32. Rosenbaum PR, Rubin DB. The Central Role of the Propensity Score in Observational Studies for Causal Effects. Biometrika. 1983;70(1):41–55.

33. Austin PC, Stuart EA. Moving towards best practice when using inverse probability of treatment weighting (IPTW) using the propensity score to estimate causal treatment effects in observational studies. Stat Med. 2015 Dec 10;34(28):3661–79.

34. Rubin DB. Using Propensity Scores to Help Design Observational Studies: Application to the Tobacco Litigation. Health Services & Outcomes Research Methodology. 2001 Dec 1;2(3):169–88.

35. Stuart EA. Matching methods for causal inference: A review and a look forward. Stat Sci. 2010 Feb 1;25(1):1–21.

36. Mölken MR, Karimi M, Leijten F, Hoedemakers M, Looman W, Islam K, et al. Comparing patients' and other stakeholders' preferences for outcomes of integrated care for multimorbidity: a discrete choice experiment in eight European countries. BMJ Open. 2020 Oct 1;10(10):e037547.

37. Publications [Internet]. Selfie 2020 -. [cited 2021 Feb 1]. Available from: https://www.selfie2020.eu/publications/.

For peer review only - http://bmjopen.bmj.com/site/about/guidelines.xhtml

38. Baxter S, Johnson M, Chambers D, Sutton A, Goyder E, Booth A. The effects of integrated care: a systematic review of UK and international evidence. BMC Health Serv Res. 2018 May 10;18(1):350.

39. Wodchis WP, Dixon A, Anderson GM, Goodwin N. Integrating care for older people with complex needs: key insights and lessons from a seven-country cross-case analysis. International Journal of Integrated Care. 2015;15(6).

40. Blom JW, Van den Hout WB, Den Elzen WPJ, Drewes YM, Bleijenberg N, Fabbricotti IN, et al. Effectiveness and cost-effectiveness of proactive and multidisciplinary integrated care for older people with complex problems in general practice: an individual participant data meta-analysis. Age and Ageing. 2018 Sep 1;47(5):705–14.

41. Bauhoff S. Systematic self-report bias in health data: impact on estimating cross-sectional and treatment effects. Health Serv Outcomes Res Method. 2011 Jul 1;11(1):44–53.

42. Liljas AEM, Brattström F, Burström B, Schön P, Agerholm J. Impact of Integrated Care on Patient-Related Outcomes Among Older People – A Systematic Review. International Journal of Integrated Care. 2019 Jul 24;19(3):6.

43. Marsh K, IJzerman M, Thokala P, Baltussen R, Boysen M, Kaló Z, et al. Multiple Criteria Decision Analysis for Health Care Decision Making—Emerging Good Practices: Report 2 of the ISPOR MCDA Emerging Good Practices Task Force. Value in Health. 2016 Mar 1;19(2):125–37.

44. Marsh KD, Sculpher M, Caro JJ, Tervonen T. The Use of MCDA in HTA: Great Potential, but More Effort Needed. Value in Health. 2018 Apr 1;21(4):394–7.

45. Baltussen R, Marsh K, Thokala P, Diaby V, Castro H, Cleemput I, et al. Multicriteria Decision Analysis to Support Health Technology Assessment Agencies: Benefits, Limitations, and the Way Forward. Value in Health. 2019 Nov 1;22(11):1283–8.

46. Sculpher M, Claxton K, Pearson SD. Developing a Value Framework: The Need to Reflect the Opportunity Costs of Funding Decisions. Value in Health. 2017 Feb;20(2):234–9.

For peer review only - http://bmjopen.bmj.com/site/about/guidelines.xhtml

519 **Figure caption**

520 **Figure 1.** Flow chart of patient participation

521

For peer review only

For peer review only - http://bmjopen.bmj.com/site/about/guidelines.xhtml

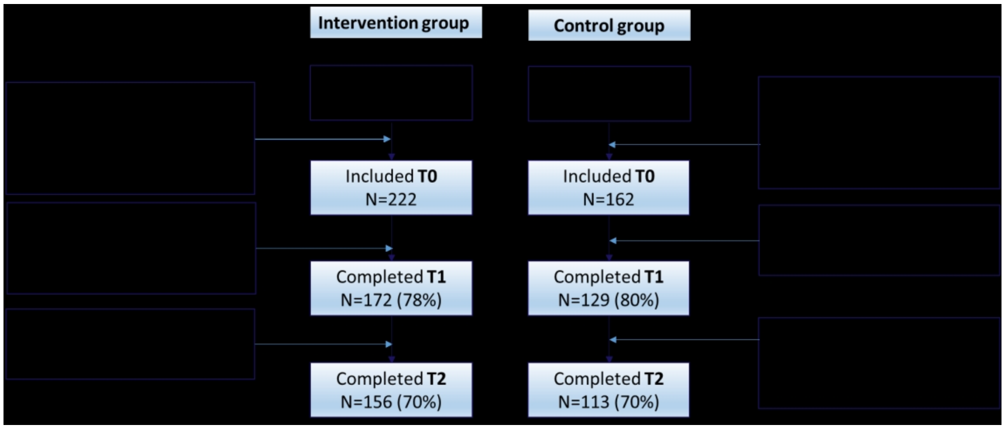

Figure 1. Flow chart of patient participation

833x351mm (59 x 59 DPI)

Supplementary files

Appendix 1: Equation relative standardization 2
Appendix 2. Relative DCE weights (0-1) (SE) of the outcomes used in the MCDA by type of stakeholder 3
Appendix 3: Graph common support 4
Appendix 4: Predicted mean performance scores of all outcome measures on their natural scale...... 5
Appendix 5: Comparison of baseline characteristics and outcome scores of dropouts versus non-dropouts........ 6
Appendix 6a: Value scores in the Multi-Criteria Decision Analysis at 6 months without costs 8
Appendix 6b: Value scores in the Multi-Criteria Decision Analysis at 12 months without costs 9

For peer review only

For peer review only - http://bmjopen.bmj.com/site/about/guidelines.xhtml

Appendix 1: Equation relative standardization

$$S_{aj} = \frac{x_{aj}}{\left(x_{aj}^2 + x_{bj}^2\right)^{1/2}}$$

Where

x = predicted mean score on the natural scale

a = the intervention group

b = the control group

j = outcome measure *j*

Note: For all outcomes in the MCDA, a higher score indicates better performance. To achieve this, in the above-mentioned equation, x is replaced by $1/x$ for outcomes where a higher score on the natural scale indicates a worse performance (e.g., costs).

For peer review only

For peer review only - http://bmjopen.bmj.com/site/about/guidelines.xhtml

Appendix 2. Relative DCE weights (0-1) (SE) of the outcomes used in the MCDA by type of stakeholder

	**Patients (n=156)**	**Partners (n=158)**	**Professionals (n=155)**	**Payers (n=104)**	**Policymakers (n=151)**
Physical functioning	0.16 (0.15)	0.11 (0.12)	0.12 (0.12)	0.14 (0.13)	0.14 (0.14)
Psychological well-being	0.17 (0.15)	0.15 (0.14)	0.18 (0.16)	0.18 (0.17)	0.15 (0.15)
Enjoyment of life	0.23 (0.18)	0.25 (0.19)	0.22 (0.19)	0.24 (0.23)	0.22 (0.19)
Social participation & relationships	0.08 (0.10)	0.09 (0.11)	0.11 (0.11)	0.10 (0.11)	0.10 (0.10)
Resilience	0.15 (0.13)	0.14 (0.13)	0.13 (0.12)	0.11 (0.10)	0.14 (0.13)
Person-centeredness	0.08 (0.10)	0.08 (0.10)	0.08 (0.09)	0.06 (0.08)	0.08 (0.09)
Continuity of care	0.10 (0.11)	0.12 (0.12)	0.11 (0.11)	0.08 (0.09)	0.10 (0.10)
Total health and social care costs	0.03 (0.08)	0.06 (0.09)	0.06 (0.09)	0.08 (0.08)	0.07 (0.09)

Note: Numbers in parentheses (n) by stakeholders indicate the number of participants included in the weight elicitation study

For peer review only

Appendix 3: Graph common support

Graph 1: Common Support of the propensity scores when matching includes the baseline values of the following variables: age, gender, marital status, living situation, educational level, smoking status, and total health and social care costs (minus medication) of three months prior to baseline as a proxy for severity

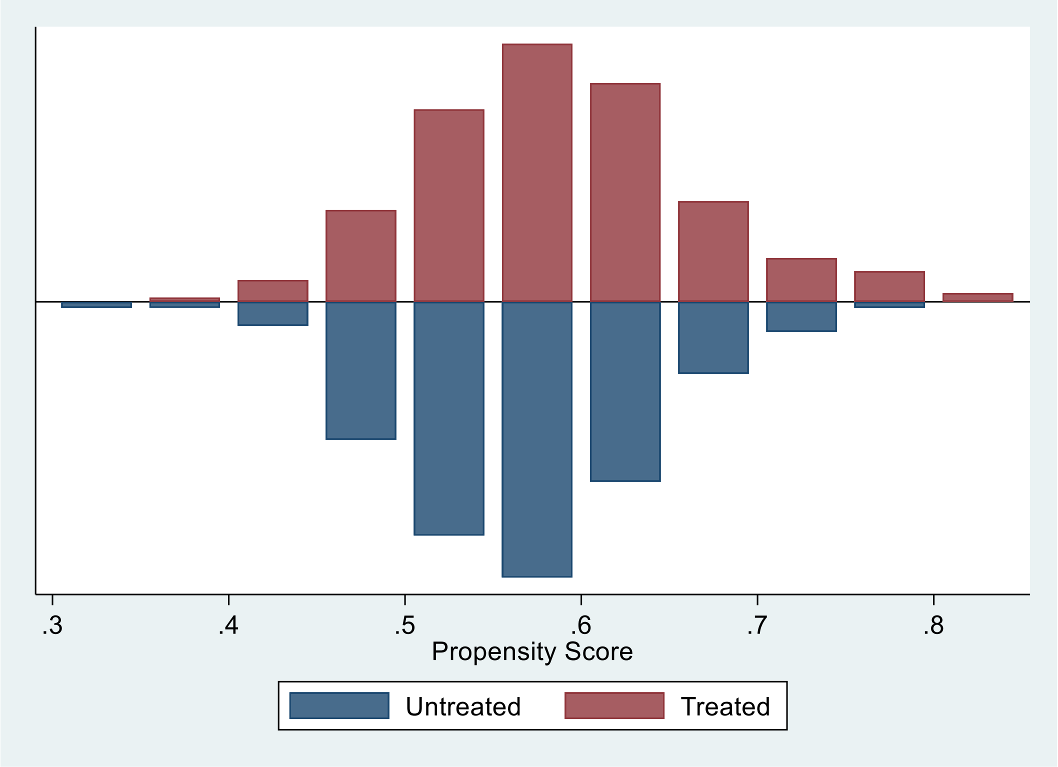

For peer review only - http://bmjopen.bmj.com/site/about/guidelines.xhtml

Appendix 4: Predicted mean performance scores of all outcome measures on their natural scale

	T0#		T1		T2	
**Outcomes measures**	**CCFE (n=222)**	**UC (n=162)**	**CCFE (n=172)**	**UC (n=129)**	**CCFE (n=156)**	**UC (n=113)**
**Health & Well-being**						
Physical functioning (0-15) ^	4.383	4.383	5.126	4.653	5.715	5.328
Psychological well-being (0-100)	71.351	71.351	70.707	71.010	69.998	71.256
Enjoyment of life (1-4)	2.820	2.820	2.917	2.741	2.874	2.839
Social relationships & participation (7-35) ^	9.167	9.167	9.273	9.579	9.424	9.779
Resilience (6-30)	19.317	19.317	19.299	19.108	19.347	19.426
**Experience of care**						
Person-centeredness (0-18)	11.729	11.729	12.828	11.353	13.060	11.727
Continuity of care (1-5)	3.672	3.672	3.771	3.653	3.833	3.704
**Costs**						
Total health and social care costs ^	5453	5453	10858	10199	22676	20659
**Additional frail elderly-specific outcomes**						
Autonomy (7-35)	22.284	22.284	21.785	22.306	21.790	22.834
Burden of medication (0-10) ^	2.088	2.088	2.221	1.878	2.263	1.480

# Note that the intervention coefficient was included in the calculation of the mean performance scores in the control group, assuming the control group had the same baseline scores as the intervention group to make it possible to directly compare the intervention and control group. CCFE=Care Chain Frail Elderly. UC=Usual care.

For peer review only - http://bmjopen.bmj.com/site/about/guidelines.xhtml

Appendix 5: Comparison of baseline characteristics and outcome scores of dropouts versus non-dropouts

	Across CCFE and UC			CCFE separately			UC separately		
**Baseline characteristics**	**Dropouts (n=115)**	**Non-dropouts (n=269)**	**P-value**	**Dropouts (n=66)**	**Non-dropouts (n=156)**	**P-value**	**Dropouts (n=49)**	**Non-dropouts (n=113)**	**P-value**
**Intervention group**	66 (57%)	156 (58%)	0.913	-	-	-	-	-	-
**Age**	85.00	83.56	0.036*	83.91	83.21	0.449	86.47	84.05	0.016*
**Gender** (female)	61%	65%	0.434	62%	64%	0.779	59%	66%	0.381
**Living situation**			0.926			0.842			0.711
Independent, alone	57%	57%		53%	54%		63%	60%	
With other(s)	43%	43%		47%	46%		37%	40%	
**Marital status**			0.162			0.149			0.734
Single, never married	1%	5%		0%	5%		2%	4%	
Married or living together	41%	44%		44%	45%		37%	42%	
Widow(er)	53%	45%		52%	42%		55%	49%	
Divorced	5%	7%		5%	8%		6%	4%	
**Educational level**			0.923			0.839			0.981
Low	70%	71%		68%	71%		71%	70%	
Middle	19%	17%		23%	19%		14%	15%	
High	11%	12%		9%	10%		14%	15%	
**Smoking** (yes)	15%	11%	0.269	18%	13%	0.299	10%	8%	0.641
**Baseline outcome scores**									
Physical functioning (0-15) ^	5.18	4.10	0.001*	4.89	4.17	0.081	5.56	4.02	0.003*
Psychological well-being (0-100)	69.37	72.39	0.145	68.06	72.74	0.092	71.17	71.89	0.815
Enjoyment of life (1-4)	2.81	2.89	0.351	2.74	2.85	0.349	2.90	2.95	0.725

For peer review only - http://bmjopen.bmj.com/site/about/guidelines.xhtml

	Across CCFE and UC			CCFE separately			UC separately		
**Baseline characteristics**	**Dropouts (n=115)**	**Non-dropouts (n=269)**	**P-value**	**Dropouts (n=66)**	**Non-dropouts (n=156)**	**P-value**	**Dropouts (n=49)**	**Non-dropouts (n=113)**	**P-value**
Social relationships and participation (7-35) ^	8.86	8.55	0.461	9.48	9.03	0.451	8.00	7.88	0.831
Resilience (6-30)	18.78	19.38	0.212	18.97	19.46	0.448	18.52	19.27	0.298
Autonomy (7-35)	21.81	22.35	0.245	21.89	22.45	0.386	21.69	22.21	0.452
Person-centeredness (0-18)	12.29	11.97	0.473	11.86	11.66	0.730	12.88	12.4	0.481
Continuity of care (1-5)	3.70	3.72	0.838	3.65	3.69	0.743	3.78	3.77	0.939
Burden of medication (0-10) ^	2.17	2.24	0.809	2.12	2.07	0.908	2.22	2.47	0.604
Total costs 3 months prior to the study (€) ^	5,989	5,112	0.247	5,797	5,308	0.635	6,248	4,842	0.208

*^ Higher score indicating worse performance. * p<0.05. CCFE=Care Chain Frail Elderly. UC=Usual care.*

For peer review only

Appendix 6a: Value scores in the Multi-Criteria Decision Analysis at 6 months without costs

			Patients		Partners		Professionals		Payers		Policymakers	
Outcome measures	Standardised performance score#		Weighted score		Weighted score		Weighted score		Weighted score		Weighted score	
	CCFE	UC	CCFE	UC	CCFE	UC	CCFE	UC	CCFE	UC	CCFE	UC
Physical functioning	0.672	0.740	0.111	0.122	0.079	0.087	0.085	0.094	0.103	0.114	0.101	0.111
Psychological well-being	0.706	0.709	0.124	0.124	0.113	0.113	0.134	0.134	0.140	0.140	0.114	0.114
Enjoyment of life	0.729	0.685	0.173	0.162	0.194	0.182	0.169	0.159	0.192	0.181	0.172	0.162
Social relationships and participation	0.718	0.696	0.059	0.057	0.069	0.067	0.083	0.081	0.079	0.076	0.077	0.075
Resilience	0.711	0.704	0.110	0.109	0.106	0.105	0.097	0.096	0.086	0.085	0.107	0.106
Person-centeredness	0.749	0.663	0.062	0.055	0.064	0.056	0.063	0.056	0.049	0.044	0.064	0.057
Continuity of care	0.718	0.696	0.074	0.072	0.092	0.089	0.083	0.081	0.063	0.061	0.077	0.075
**Overall value scores**			**0.712**	**0.701**	**0.715**	**0.698**	**0.714**	**0.700**	**0.713**	**0.701**	**0.713**	**0.700**

# colour scheme ranges from red (lowest score) to green (highest score). CCFE=Care Chain Frail Elderly. UC=Usual care.

For peer review only - http://bmjopen.bmj.com/site/about/guidelines.xhtml

Appendix 6b: Value scores in the Multi-Criteria Decision Analysis at 12 months without costs

			Patients		Partners		Professionals		Payers		Policymakers	
**Outcome measures**	**Standardised performance score#**		**Weighted score**		**Weighted score**		**Weighted score**		**Weighted score**		**Weighted score**	
	**CCFE**	**UC**	**CCFE**	**UC**	**CCFE**	**UC**	**CCFE**	**UC**	**CCFE**	**UC**	**CCFE**	**UC**
Physical functioning	0.682	0.731	0.112	0.121	0.080	0.086	0.086	0.092	0.105	0.113	0.103	0.110
Psychological well-being	0.701	0.713	0.123	0.125	0.112	0.114	0.133	0.135	0.139	0.141	0.113	0.115
Enjoyment of life	0.711	0.703	0.169	0.167	0.189	0.187	0.165	0.163	0.188	0.185	0.168	0.166
Social relationships and participation	0.720	0.694	0.059	0.057	0.069	0.066	0.083	0.080	0.079	0.076	0.077	0.075
Resilience	0.706	0.709	0.109	0.110	0.105	0.106	0.097	0.097	0.085	0.086	0.106	0.107
Person-centeredness	0.744	0.668	0.061	0.055	0.063	0.057	0.063	0.056	0.049	0.044	0.064	0.057
Continuity of care	0.719	0.695	0.074	0.072	0.092	0.089	0.083	0.080	0.063	0.061	0.077	0.075
**Overall value scores**			**0.708**	**0.706**	**0.710**	**0.704**	**0.710**	**0.704**	**0.708**	**0.706**	**0.709**	**0.705**

colour scheme ranges from red (lowest score) to green (highest score). CCFE=Care Chain Frail Elderly. UC=Usual care.

For peer review only - http://bmjopen.bmj.com/site/about/guidelines.xhtml

The RECORD statement – checklist of items, extended from the STROBE statement, that should be reported in observational studies using routinely collected health data.

	Item No.	STROBE items	Location in manuscript where items are reported	RECORD items	Location in manuscript where items are reported
**Title and abstract**					
	1	(a) Indicate the study's design with a commonly used term in the title or the abstract (b) Provide in the abstract an informative and balanced summary of what was done and what was found	1,3	RECORD 1.1: The type of data used should be specified in the title or abstract. When possible, the name of the databases used should be included.<br><br>RECORD 1.2: If applicable, the geographic region and timeframe within which the study took place should be reported in the title or abstract.<br><br>RECORD 1.3: If linkage between databases was conducted for the study, this should be clearly stated in the title or abstract.	1,3
**Introduction**					
Background rationale	2	Explain the scientific background and rationale for the investigation being reported	5,6		
Objectives	3	State specific objectives, including any prespecified hypotheses	6		
**Methods**					
Study Design	4	Present key elements of study design early in the paper	7,8		
Setting	5	Describe the setting, locations, and relevant dates, including periods of recruitment, exposure, follow-up, and data collection	7-9		

For peer review only

Participants	6	*(a) Cohort study* - Give the eligibility criteria, and the sources and methods of selection of participants. Describe methods of follow-up<br>*Case-control study* - Give the eligibility criteria, and the sources and methods of case ascertainment and control selection. Give the rationale for the choice of cases and controls<br>*Cross-sectional study* - Give the eligibility criteria, and the sources and methods of selection of participants<br><br>*(b) Cohort study* - For matched studies, give matching criteria and number of exposed and unexposed<br>*Case-control study* - For matched studies, give matching criteria and the number of controls per case	8-9<br><br>10	RECORD 6.1: The methods of study population selection (such as codes or algorithms used to identify subjects) should be listed in detail. If this is not possible, an explanation should be provided.<br><br>RECORD 6.2: Any validation studies of the codes or algorithms used to select the population should be referenced. If validation was conducted for this study and not published elsewhere, detailed methods and results should be provided.<br><br>RECORD 6.3: If the study involved linkage of databases, consider use of a flow diagram or other graphical display to demonstrate the data linkage process, including the number of individuals with linked data at each stage.	
Variables	7	Clearly define all outcomes, exposures, predictors, potential confounders, and effect modifiers. Give diagnostic criteria, if applicable.	9	RECORD 7.1: A complete list of codes and algorithms used to classify exposures, outcomes, confounders, and effect modifiers should be provided. If these cannot be reported, an explanation should be provided.	
Data sources/ measurement	8	For each variable of interest, give sources of data and details of methods of assessment (measurement).<br>Describe comparability of assessment methods if there is more than one group	9-10		

For peer review only

Bias	9	Describe any efforts to address potential sources of bias	10		
Study size	10	Explain how the study size was arrived at	see study protocol		
Quantitative variables	11	Explain how quantitative variables were handled in the analyses. If applicable, describe which groupings were chosen, and why	10		
Statistical methods	12	(a) Describe all statistical methods, including those used to control for confounding<br>(b) Describe any methods used to examine subgroups and interactions<br>(c) Explain how missing data were addressed<br>(d) *Cohort study* - If applicable, explain how loss to follow-up was addressed<br>*Case-control study* - If applicable, explain how matching of cases and controls was addressed<br>*Cross-sectional study* - If applicable, describe analytical methods taking account of sampling strategy<br>(e) Describe any sensitivity analyses	10-11<br>11		
Data access and cleaning methods		..		RECORD 12.1: Authors should describe the extent to which the investigators had access to the database population used to create the study population.	NA

For peer review only

				RECORD 12.2: Authors should provide information on the data cleaning methods used in the study.	NA
Linkage		..		RECORD 12.3: State whether the study included person-level, institutional-level, or other data linkage across two or more databases. The methods of linkage and methods of linkage quality evaluation should be provided.	NA
**Results**					
Participants	13	(a) Report the numbers of individuals at each stage of the study (*e.g.*, numbers potentially eligible, examined for eligibility, confirmed eligible, included in the study, completing follow-up, and analysed)<br>(b) Give reasons for non-participation at each stage.<br>(c) Consider use of a flow diagram	12	RECORD 13.1: Describe in detail the selection of the persons included in the study (*i.e.*, study population selection) including filtering based on data quality, data availability and linkage. The selection of included persons can be described in the text and/or by means of the study flow diagram.	13
Descriptive data	14	(a) Give characteristics of study participants (*e.g.*, demographic, clinical, social) and information on exposures and potential confounders<br>(b) Indicate the number of participants with missing data for each variable of interest<br>(c) *Cohort study* - summarise follow-up time (*e.g.*, average and total amount)	12<br><br>12<br><br>8		
Outcome data	15	*Cohort study* - Report numbers of outcome events or summary measures over time<br>*Case-control study* - Report numbers in each exposure	13		

For peer review only

		category, or summary measures of exposure<br>*Cross-sectional study* - Report numbers of outcome events or summary measures			
Main results	16	(a) Give unadjusted estimates and, if applicable, confounder-adjusted estimates and their precision (e.g., 95% confidence interval). Make clear which confounders were adjusted for and why they were included<br>(b) Report category boundaries when continuous variables were categorized<br>(c) If relevant, consider translating estimates of relative risk into absolute risk for a meaningful time period	13		
Other analyses	17	Report other analyses done—e.g., analyses of subgroups and interactions, and sensitivity analyses	13		
**Discussion**					
Key results	18	Summarise key results with reference to study objectives	14		
Limitations	19	Discuss limitations of the study, taking into account sources of potential bias or imprecision. Discuss both direction and magnitude of any potential bias	15	RECORD 19.1: Discuss the implications of using data that were not created or collected to answer the specific research question(s). Include discussion of misclassification bias, unmeasured confounding, missing data, and changing eligibility over time, as they pertain to the study being reported.	NA
Interpretation	20	Give a cautious overall interpretation of results considering objectives,	14,15		

For peer review only - http://bmjopen.bmj.com/site/about/guidelines.xhtml

		limitations, multiplicity of analyses, results from similar studies, and other relevant evidence			
Generalisability	21	Discuss the generalisability (external validity) of the study results	15		
**Other Information**					
Funding	22	Give the source of funding and the role of the funders for the present study and, if applicable, for the original study on which the present article is based	4		
Accessibility of protocol, raw data, and programming code		..		RECORD 22.1: Authors should provide information on how to access any supplemental information such as the study protocol, raw data, or programming code.	

*Reference: Benchimol EI, Smeeth L, Guttmann A, Harron K, Moher D, Petersen I, Sørensen HT, von Elm E, Langan SM, the RECORD Working Committee. The REporting of studies Conducted using Observational Routinely-collected health Data (RECORD) Statement. *PLoS Medicine* 2015; in press.

*Checklist is protected under Creative Commons Attribution (CC BY) license.

For peer review only

BMJ Open

Value-based person-centred integrated care for frail elderly living at home: a quasi-experimental evaluation using Multi-Criteria Decision Analysis

Journal:	*BMJ Open*
Manuscript ID	bmjopen-2021-054672.R1
Article Type:	Original research
Date Submitted by the Author:	12-Nov-2021
Complete List of Authors:	Hoedemakers, Maaike; Erasmus Universiteit Rotterdam, Erasmus School of Health Policy & Management<br>Karimi, Milad; Erasmus University Rotterdam<br>Leijten, Fenna; Erasmus Universiteit Rotterdam, Health Policy & Management ; Ministry of defence, Staff Defence Healthcare Organisation<br>Goossens, Lucas; Erasmus University<br>Islam, Kamrul; University of Bergen, Department of Economics; NORCE Norwegian Research Centre AS, Social sciences<br>Tsiachristas, Apostolos; University of Oxford, Nuffield Department of Population Health<br>Rutten-van Molken, Maureen; Erasmus University Rotterdam; Erasmus University Rotterdam
**Primary Subject Heading**:	Health policy
Secondary Subject Heading:	Health services research, Health policy
Keywords:	Health economics < HEALTH SERVICES ADMINISTRATION & MANAGEMENT, HEALTH ECONOMICS, Health policy < HEALTH SERVICES ADMINISTRATION & MANAGEMENT, Organisation of health services < HEALTH SERVICES ADMINISTRATION & MANAGEMENT

SCHOLARONE™
Manuscripts

I, the Submitting Author has the right to grant and does grant on behalf of all authors of the Work (as defined in the below author licence), an exclusive licence and/or a non-exclusive licence for contributions from authors who are: i) UK Crown employees; ii) where BMJ has agreed a CC-BY licence shall apply, and/or iii) in accordance with the terms applicable for US Federal Government officers or employees acting as part of their official duties; on a worldwide, perpetual, irrevocable, royalty-free basis to BMJ Publishing Group Ltd ("BMJ") its licensees and where the relevant Journal is co-owned by BMJ to the co-owners of the Journal, to publish the Work in this journal and any other BMJ products and to exploit all rights, as set out in our licence.

The Submitting Author accepts and understands that any supply made under these terms is made by BMJ to the Submitting Author unless you are acting as an employee on behalf of your employer or a postgraduate student of an affiliated institution which is paying any applicable article publishing charge ("APC") for Open Access articles. Where the Submitting Author wishes to make the Work available on an Open Access basis (and intends to pay the relevant APC), the terms of reuse of such Open Access shall be governed by a Creative Commons licence – details of these licences and which Creative Commons licence will apply to this Work are set out in our licence referred to above.

Other than as permitted in any relevant BMJ Author's Self Archiving Policies, I confirm this Work has not been accepted for publication elsewhere, is not being considered for publication elsewhere and does not duplicate material already published. I confirm all authors consent to publication of this Work and authorise the granting of this licence.

For peer review only

Title

Value-based person-centred integrated care for frail elderly living at home: a quasi-experimental evaluation using Multi-Criteria Decision Analysis

Authors

Maaike Hoedemakers, MSc[1]; Milad Karimi, PhD[1,2]; Fenna Leijten, PhD[1,3]; Lucas Goossens, PhD [1]; Kamrul Islam, PhD [4,5]; Apostolos Tsiachristas, PhD [6]; Maureen Rutten-van Mölken, PhD [1,7]

Affiliations

1: Erasmus School of Health Policy & Management, Erasmus University Rotterdam, the Netherlands

2: Pharmerit, Rotterdam, the Netherlands

3: Staff Defence Healthcare Organisation, Ministry of Defence, Utrecht, the Netherlands

4: Department of Economics, University of Bergen, Bergen, Norway

5: Health Services and Health Economics, NORCE Norwegian Research Centre, Bergen, Norway

6: Health Economics Research Centre, Nuffield Department of Population Health, University of Oxford, Oxford, United Kingdom

7: Institute for Medical Technology Assessment, Erasmus University Rotterdam, Rotterdam, the Netherlands

Contact information corresponding author

Maureen Rutten-van Mölken

Burgemeester Oudlaan 50, 3062 PA Rotterdam

T: +31 10 408 9056

E-mail: m.rutten@eshpm.eur.nl

For peer review only - http://bmjopen.bmj.com/site/about/guidelines.xhtml

Word count: 3680

Abstract

Objective: To evaluate the value of the person-centred, integrated care programme Care Chain Frail Elderly (CCFE) compared with usual care, using Multi-Criteria Decision Analysis (MCDA).

Design: In a 12-month quasi-experimental study, Triple-Aim outcomes were measured at 0, 6 and 12 months by trained interviewers during home-visits.

Setting: Primary care, community-based elderly care.

Participants: 384 community-dwelling frail elderly were enrolled. The 12-month completion rate was 70% in both groups. Propensity score matching was used to balance age, gender, marital status, living situation, education, smoking status, and three-month costs prior to baseline between the two groups.

Intervention: The CCFE is an integrated care programme with unique features like the presence of the elderly and informal caregiver at the multi-disciplinary team meetings, and a bundled payment.

Primary and secondary outcomes measures: The MCDA results in weighted overall value scores that combines the performance on physical functioning, psychological well-being, social relationships and participation, enjoyment of life, resilience, person-centeredness, continuity of care, and costs, with importance weights of patients, informal caregivers, professionals, payers, and policymakers.

Results: At 6 months, the overall value scores of CCFE were higher in all stakeholder groups, driven by enjoyment of life (standardised performance scores 0.729 versus 0.685) and person-centeredness (0.749 versus 0.663). At 12 months, the overall value scores in both groups were similar from a patient's perspective, slightly higher for CCFE from an informal caregiver's and professional's perspective, and

For peer review only - http://bmjopen.bmj.com/site/about/guidelines.xhtml

lower for CCFE from a payer's and policymaker's perspective. The latter was driven by a worse performance on physical functioning (0.682 versus 0.731) and higher costs (€22,816 versus €20,680).

Conclusions: The MCDA indicated that the CCFE is the preferred way of delivering care to frail elderly at 6 months. However, at 12 months, MCDA-results showed little difference from the perspective of patients, informal caregivers, and professionals, while payers and policy makers seemed to prefer usual care.

For peer review only

Key words: Multi-Criteria Decision Analysis, integrated care, frail elderly, triple aim, patient-reported outcome measures

For peer review only - http://bmjopen.bmj.com/site/about/guidelines.xhtml

53 **Strengths and limitations of this study**

54 - We implemented a novel multi-criteria decision analysis (MCDA) framework to evaluate a
55 person-centred integrated care programme for frail elderly.
56 - We measured a broad range of Triple-Aim outcomes at three time points, among a large and
57 difficult to reach group of the frailest elderly living at home.
58 - The choice of outcome measures was largely driven by focus groups involving frail elderly.
59 - The different outcome measures were weighted by their importance, from multiple
60 perspectives, to calculate overall value scores.
61 - Due to the choice of measuring patient-reported outcomes, participation was too burdensome
62 or impossible for frail elderly with cognitive impairments.

For peer review only

For peer review only - http://bmjopen.bmj.com/site/about/guidelines.xhtml

INTRODUCTION

The Care Chain Frail Elderly (CCFE) is an integrated care programme for community-dwelling frail elderly in the Netherlands that offers person-centred care, coordination, and case management to support elderly in living at home for as long as possible.[1] It was developed as part of a movement towards decentralization of long-term care, increased self-sufficiency, and societal participation that was stimulated by the Dutch government to maintain affordability of elderly care. This movement led to a reform of the long-term care sector in 2015.[2] The number of nursing- and residential homes was reduced considerably and access to nursing homes was restricted to those in need of 24-hour care. Municipalities became responsible for the provision of homecare and social support, and health insurers for financing nursing care at home. While the importance of homecare was growing, this sector was confronted with significant budget cuts.[3] The reform accelerated the development of integrated-care initiatives, which were spearheaded by the National Care for the Elderly-Programme commissioned by the Ministry of Health.[4,5]

Integrated care for older people often comprises similar components, namely, comprehensive assessment, individualised care planning, case management, and multidisciplinary team meetings.[6,7] All of these elements are also present in the CCFE. In addition, the intervention is unique in several respects. First, the older person and their informal caregiver are present at the multidisciplinary team meetings where the results of the comprehensive assessment and the individual care plan are discussed. Second, the CCFE is financed by a bundled payment aiming to stimulate collaboration between professionals. The bundled payment is a fixed amount of money per patient that covers all services provided by the GP, nurse-practitioner and physician assistant, regardless of diagnoses, medication review by the pharmacist, telephone consultation by the geriatrician, non-individual-patient-related activities such as building a community network, and overhead. Third, it targets the top-1% frailest elderly registered in a GP-practice who are living at home with complex care needs, using a case-finding approach. The CCFE aims

For peer review only - http://bmjopen.bmj.com/site/about/guidelines.xhtml

to better integrate care across sectors and build a network of support around the patient, and thereby to improve their physical, mental, social health and well-being and experience with care. Ideally, this also reduces secondary care and residential long-term care utilization and thereby costs. The CCFE has previously been described in detail elsewhere.[1]

Although such integrated-care programmes are designed to meet the older person's needs, previous studies show mixed (cost-)effectiveness results.[8–10] A potential explanation is that common methods to assess effectiveness tend to focus on measuring traditional outcomes, such as physical functioning, whereas that is not the primary focus of these programmes.[8,11] Cost-effectiveness analyses measure Quality-Adjusted Life-Year (QALY) gains, which is also less appropriate because integrated care for frail elderly focuses more on well-being than on survival and health-related quality of life.[12] For example, elderly are stimulated to visit outpatient day-care activities to enhance their social participation, or their experience with care is improved by individual care planning and helping them navigate through the health- and social-care system. Accordingly, empirical evaluations should include these domains to accurately value the potential benefits of an intervention.[13,14]

Therefore, we adopted a broader evaluation method, namely Multi-Criteria Decision Analysis (MCDA) to evaluate the CCFE. MCDA is innovative in elderly care because it synthesises a wide variety of outcome measures, in this case patient-reported health and well-being measures (PROMS), experience with care measures (PREMS), and costs. Together, they cover the Triple Aim.[15] In the MCDA outcomes are weighted by their importance according to five stakeholder groups: patients, informal caregivers, professionals, policymakers, and payers. The weighted performance scores are aggregated into overall value scores.[16]

For peer review only - http://bmjopen.bmj.com/site/about/guidelines.xhtml

METHODS

Study population

The study population consisted of community-dwelling frail elderly, where frailty is defined as being in need of complex care due to loss of functional abilities and control over one's life. To participate in the intervention, they had to be registered at a GP-practice from one of three care groups (i.e., an association of primary care providers that cooperate in the provision of chronic care) that offer the CCFE. They had to be able to comprehend study information and answer questions, either independently or with the help of an informal caregiver or trained interviewer.

Intervention

A primary care team consisting of the GP, nurse-practitioner, and district nurse, identifies potential candidates for the CCFE using a case-finding approach. To be included in the CCFE, the health insurer does not require a specific diagnosis or the use of a screening tool. They trust that the GP and nurse practitioner know their patients and are most suited to select the elderly that may benefit from the care programme. Furthermore, there is an agreement with the health insurer that only the top 1% frail elderly of a GP-practice will be included in the programme. Subsequently, the nurse-practitioner visits the older person at home and performs a comprehensive geriatric assessment of the needs, capabilities, and preferences in the physical, psychological, cognitive, and social domains. Depending on this assessment, the nurse-practitioner consults professionals in the community (e.g., physical therapists, occupational therapists, social workers, elderly care physicians, geriatricians, dementia case workers) and arranges informal care support. Together with the frail elderly and informal caregiver, the nurse-practitioner drafts an individual care plan, largely driven by the elderly's personal goals.

The nurse-practitioner organises multidisciplinary team meetings to discuss the individual care plan with all professionals involved. The frail elderly and their informal caregiver participate in these meetings.

For peer review only - http://bmjopen.bmj.com/site/about/guidelines.xhtml

131 During the first meeting a case manager is assigned, a role mostly taken up by the nurse-practitioner. 132 The case manager is the main contact point, monitors the execution of the individual care plan, and 133 further adapts the care to the patient's wishes and additional needs. Once enrolled, an elderly person 134 usually stays in the CCFE until nursing home admission or if they pass away.

135 To support collaboration between professionals, the CCFE uses 'Care2U', a secured ICT-platform to share 136 information. Professionals have different degrees of access and the elderly must approve access. Care2U 137 includes the individual care plan and is used by the nurse-practitioner to monitor appointments and 138 services of providers in the care chain.

139 The CCFE is financed by a bundled payment contract between each care group and the dominant health 140 insurer in the region and is re-negotiated every year. These care groups are legal entities of primary care 141 providers who develop chronic care programs, support the provision of these programmes and are 142 contracted by the health insurer to coordinate chronic care in a region.

143 *Study design*

144 The MCDA was conducted as part of a 12-month prospective quasi-experimental study comparing two 145 parallel groups:

146 *Intervention group* included frail elderly enrolled into the CCFE between April 2017-August 2018 and 147 were recruited to participate in the study by either their GP or nurse-practitioner.

148 *Control group* consisted of frail elderly receiving usual care, recruited at GP-practices from one of three 149 participating care groups. These practices had not (yet) implemented the CCFE. To ensure a similar level 150 of frailty in both groups, the GP-practices in the control group applied the same case-finding approach. 151 They were assisted by a GP specialised in elderly care that had experience with the CCFE.

For peer review only - http://bmjopen.bmj.com/site/about/guidelines.xhtml

Self-reported outcome and experience measures and care utilization data were gathered at baseline and after 6 months and 12 months, during face-to-face interviews at the elderly's home, performed by trained interviewers. The interviewers were not involved in delivering any aspects of the intervention but were aware of which group the respondent belonged to. Data collection took place between April 2017-August 2019.

Outcome measures

Table 1 gives an overview of all outcome measures. These outcomes were selected based on a literature review, workshops with representatives from the five stakeholder groups, and focus groups with individuals with multi-morbidity, and measured with validated questionnaires.[17]

Table 1. Outcome measures and instruments

Core set^	Instrument to measure outcome	Scale
**Health & Well-being**		
Physical functioning	Activities of Daily Living (ADL) (Katz-15)[18]	0-15 (worst)
Psychological well-being	Mental Health Inventory (MHI-5)[19]	0-100 (best)
Enjoyment of life	Investigating Choice Experiments for the Preferences of Older People (ICECAP-O)[20]	1-4 (best)
Social relationships & participation	Impact on Participation & Autonomy (IPA), social life and relationships domain[21]	0-28 (worst)
Resilience	Brief Resilience Scale (BRS)[22]	6-30 (best)
**Experience of care**		
Person-centeredness	Person-centered Coordinated Care Experience Questionnaire (P3CEQ), experience of person-centered care domain[23]	0-18 (best)
Continuity of care	Nijmegen Continuity Questionnaire (NCQ), team and cross boundary continuity domain[24] + Client Perceptions of Coordination Questionnaire (CPCQ)[25]	1-5 (best)
**Costs**		
Health, social, and informal care costs	iMTA Medical Consumption Questionnaire[Error! Reference source not found.]	

For peer review only - http://bmjopen.bmj.com/site/about/guidelines.xhtml

Medication costs	Prescriptions in patient records extracted from GP information systems	
Bundled payments and chronic care programmes	Care chain information system 'Care2U'	
**Additional outcomes**		
Autonomy	Pearlin Mastery Scale[26]	7-35 (best)
Burden of medication	Living with Medicines Questionnaire - LMQ[28]	0-10 (worst)

^The core set of outcomes was measured across all studies included in the SELFIE project. For these outcomes, weights were elicited, and these outcomes were included in the MCDA. The additional outcomes were not included in the MCDA.

Costs

Health- and social-care utilization data were obtained with the iMTA-Medical Consumption Questionnaire, which includes questions about contacts with GP, nurse-practitioner, GP-assistants, physiotherapists and other paramedical therapists, dieticians, psychologists, social workers, welfare workers, and medical specialists, hospital admissions, rehabilitation, homecare, residential care and nursing homes, and informal care during the past three months.[Error! Reference source not found.] Unit costs were largely based on reference prices from the Dutch Costing Manual.[29] Medication costs were based on prescription data from GP-information systems, which were combined with unit costs from Dutch drug database '*G-Standaard*', using ATC-codes.[30] Programme costs of the CCFE were based on the bundled payment contracts between each care group and the dominant health insurer.[1] Elderly from the control group could participate in single-disease care programmes for diabetes, cardiovascular risk management or Chronic Obstructive Pulmonary Disease. The proportion of elderly participating in these programmes was obtained from Care2U and average prices of the bundled payments were obtained from the Dutch health claims database (Vektis).[31]

Statistical analysis

Comparability of groups

For peer review only - http://bmjopen.bmj.com/site/about/guidelines.xhtml

For peer review only

We applied inverse probability weighting to increase the comparability of the intervention and control groups at baseline.[32] The logistic regression model to estimate the propensity score included age, gender, marital status, living situation, educational level, smoking status, and costs three months prior to baseline as a proxy for complexity. The propensity score *p* reflects the estimated probability of an individual to be in the intervention group. By setting the weight for individuals in the intervention group to 1, and for individuals in the control group to *p/(1-p)*, we estimated the average treatment effect in the treated (ATT) in the weighted mixed effect models described below.[33] To assess the comparability of the two groups, we calculated the mean percentage standardised bias, the Rubin's B (absolute standardised difference of the means of the linear index of the propensity score in the intervention group and matched controls), and the Rubin's R (ratio of intervention group and matched control group variances of the propensity score index). For sufficient balance, B should be less than 25% and R be between 0.5 and 2.[34]

Treatment effects

Treatment effects were estimated using weighted mixed effect models with a random intercept at individual level (as we had longitudinal data) and the following covariates: time, intervention, an interaction term for time and intervention, age, gender, marital status, living situation, educational level, and smoking status. This combination of matching and regression adjustment has been shown to best reduce covariate imbalance between groups.[35] The mixed effects models were used to predict the mean scores of the outcome measures in both groups at each time point, assuming the control group had the same baseline score as the intervention group to directly compare both groups. All statistical analyses were performed using STATA16.1.

MCDA

In the MCDA, the predicted mean scores of the outcomes at 6- and 12-months follow-up were standardised on a 0-1 scale to remove differences in measurement scales, using relative standardization,

For peer review only - http://bmjopen.bmj.com/site/about/guidelines.xhtml

205 see **Appendix 1**. For all outcomes in the MCDA, a higher score indicates better performance. The
206 standardised outcomes were weighted by their importance and subsequently summed to obtain an
207 overall value score for the intervention and control group separately. The relative importance-weights
208 were elicited in an online weight elicitation study among patients, informal caregivers, professionals,
209 payers, and policymakers, using a discrete choice experiment (DCE).[36] The relative weights of the
210 outcome measures included in the MCDA by stakeholder group can be found in **Appendix 2**.

211 Sensitivity analysis

212 To assess the joint uncertainty in outcome scores and importance-weights, we performed a probabilistic
213 sensitivity analysis using Monte Carlo simulation. We used Cholesky decomposition to obtain 10,000
214 replications of both the standardised outcomes and the importance-weights. From this, we obtained the
215 95%-confidence intervals around the overall value scores for each stakeholder group. Additionally, we
216 calculated the proportion of MCDA-iterations in which the CCFE has a higher overall value score than
217 usual care.

218 *Patient and Public Involvement*

219 Patients were involved in the selection of outcome measures, which was largely based on focus groups
220 as described above.[17] In multiple National Stakeholder Workshops held during the entire process of the
221 study, stakeholders from the five stakeholder groups were asked to reflect upon the outcome measures,
222 the study design and data collection, and the results of the study. The study design was set-up in close
223 collaboration with care providers to ensure feasibility of the data collection. Before the start of data
224 collection, the questionnaire was piloted in a frail older person. The authors disseminated results via
225 conference presentations. Results of this study were also disseminated to participating care providers,
226 patients and informal caregivers using communication methods other than scientific papers, i.e., by e-
227 mail and newsletters disseminated at the GP-practices.

For peer review only - http://bmjopen.bmj.com/site/about/guidelines.xhtml

228 *Ethics approval and consent to participate*

229 The study was conducted as one of the case studies in Horizon2020-project SELFIE (Sustainable
230 intEgrated care modeLs for multi-morbidity: delivery, FInancing and performancE).[37] The study protocol
231 was reviewed by the medical ethics committee of the Erasmus Medical Centre, the Netherlands. The
232 committee concluded that the rules laid down in the Dutch Medical Research Involving Human Subjects
233 Act do not apply to this research (*MEC-2017-121*). All study participants have provided informed written
234 consent.

For peer review only

For peer review only - http://bmjopen.bmj.com/site/about/guidelines.xhtml

RESULTS

Respondents

Figure 1 presents the flow chart of patients included in the study. The retention rate at 12-month follow-up was 70% in both groups. Main reasons of loss-to-follow-up were also similar in both groups and included death, burden of study participation, and cognitive incapacity.

*****Figure 1 here*****

Table 2 presents the baseline characteristics of respondents before and after inverse probability weighting. After IPW, the matching statistics were within the desired range (Rubin's B < 25%, Rubin's R 0.5 – 2). No substantial differences between the groups remained. The common support graph shows a good overlap in propensity scores, see **Appendix 3**.

For peer review only

For peer review only - http://bmjopen.bmj.com/site/about/guidelines.xhtml

Table 2. Baseline characteristics before and after inversed probability weighting

	CCFE N=222	UC N=162	St. diff.	UC N=162	St. diff.
**Baseline characteristics**		**Before IPW**		**After IPW**	
Age, mean	83.4	84.8	-0.224	83.6	-0.028
Female (%)	63.5	64.2	-0.014	64.8	-0.027
Marital status (%)					
Single, never married	3.6	3.7	-0.005	2.6	0.054
Married or living together	44.6	40.7	0.078	45.0	-0.008
Widow(er)	44.6	50.6	-0.120	47.1	-0.049
Divorced	7.2	4.9	0.095	5.4	0.078
Living situation (%)					
Independent, alone	54.1	61.1	-0.143	55.2	-0.024
With others	46.0	38.9	0.143	44.8	0.024
Educational level (%)					
Low	70.3	70.4	-0.002	73.3	-0.067
Medium	20.3	14.8	0.143	14.3	0.158
High	9.5	14.8	-0.164	12.4	-0.090
Current smoker (%)	14.4	8.6	0.181	13.8	0.019
**Outcome measures at baseline, mean**		**Before IPW**		**After IPW**	
Physical functioning (0-15) ^	4.4	4.5	-0.032	4.5	-0.052
Psychological well-being (0-100)	71.4	71.7	-0.018	70.6	0.040
Enjoyment of life (1-4)	2.8	2.9	-0.137	2.9	-0.143
Social relationships and participation (0-28) ^	9.2	8.0	0.345	8.2	0.280
Resilience (6-30)	19.3	19.0	0.062	19.0	0.082
Autonomy (7-35)	22.3	22.1	0.054	21.9	0.098
Burden of medication (0-10) ^	2.1	2.4	-0.111	2.5	-0.166
Person-centeredness (0-18)	11.7	12.5	-0.209	12.7	-0.246
Continuity of care (1-5)	3.7	3.8	-0.140	3.8	-0.182
Total costs 3 months prior to the study (€) ^	5,453	5,267	0.028	5,631	-0.026
**Statistics to assess matching#**		**Before IPW**		**After IPW**	
Mean bias			11.9		5.1
Rubin's B			39.7		21.4
Rubin's R			1.06		1.10

^ Higher score indicating worse performance; St. diff = Absolute Standardised Mean Difference, also called Absolute Standardised Bias; # On variables used in PSM, i.e., age, gender, marital status, living situation, educational level, smoking, total costs 3 months prior to the study. CCFE=Care Chain Frail Elderly. UC=Usual care. IPW=Inverse Probability Weighting

For peer review only

For peer review only - http://bmjopen.bmj.com/site/about/guidelines.xhtml

Treatment effects

Table 3 reports the estimated treatment effects of the CCFE at 6- and 12-months of follow-up. Results show that the CCFE improved person-centeredness at both time points. At 6 months follow-up, physical functioning declined in both groups but even further in the intervention group. At 12 months follow-up, the CCFE performed worse on autonomy and burden of medication. The predicted mean performance scores of all outcomes on their natural scale can be found in **Appendix 4**.

For peer review only

For peer review only - http://bmjopen.bmj.com/site/about/guidelines.xhtml

Table 3. Treatment effects at 6- and 12-months follow-up

	6 months			12 months		
**Core set of outcomes included in the MCDA**	**Estimated change CCFE**	**Estimated Change UC**	**Diff in change Mean (95% CI)#**	**Estimated change CCFE**	**Estimated change UC**	**Diff in change Mean (95% CI)#**
**Health & Well-being**						
Physical functioning (0-15) ^	0.74	0.27	0.47 (0.06 ; 0.88)*	1.33	0.95	0.39 (-0.10 ; 0.87)
Psychological well-being (0-100)	-0.64	-0.34	-0.30 (-3.86 ; 3.25)	-1.35	-0.10	-1.26 (-5.20 ; 2.68)
Enjoyment of life (1-4)	0.10	-0.08	0.18 (-0.02 ; 0.38)	0.05	0.02	0.04 (-0.18 ; 0.25)
Social relationships & participation (0-28) ^	0.11	0.41	-0.31 (-1.04 ; 0.43)	0.26	0.61	-0.36 (-1.25 ; 0.54)
Resilience (6-30)	-0.02	-0.21	0.19 (-0.63 ; 1.01)	0.03	0.11	-0.08 (-1.01 ; 0.85)
**Experience of care**						
Person-centeredness (0-18)	1.10	-0.38	1.48 (0.57 ; 2.38)*	1.33	0.00	1.33 (0.18 ; 2.49)*
Continuity of care (1-5)	0.10	-0.02	0.12 (-0.05 ; 0.28)	0.16	0.03	0.13 (-0.05 ; 0.31)
**Costs**						
Total health and social care costs^	5,405	4,745	660 (-1,650 ; 2,970)	17,223	15,206	2,017 (-2,361 ; 6,395)
**Additional frail elderly-specific outcomes**	**Estimated change CCFE**	**Estimated change UC**	**Diff in change Mean (95% CI)#**	**Estimated change CCFE**	**Estimated change UC**	**Diff in change Mean (95% CI)#**
Autonomy (7-35)	-0.50	0.02	-0.52 (-1.38 ; 0.34)	-0.49	0.55	-1.04 (-1.95 ; -0.14)*
Burden of medication (0-10) ^	0.13	-0.21	0.34 (-0.36 ; 1.04)	0.18	-0.61	0.78 (0.04 ; 1.53)*

*^ Higher score indicating worse performance; # based on robust SE; * p<0.05. MCDA=Multi-Criteria Decision Analysis. CCFE=Care Chain Frail Elderly. UC=Usual care. CI=Confidence interval.*

For peer review only - http://bmjopen.bmj.com/site/about/guidelines.xhtml

261 *Costs*

262 **Table 4** presents details on the mean costs after inversed probability weighting at 6- and 12-months,
263 from a healthcare and a societal perspective. From a healthcare perspective, after 6 months, costs were
264 €751 higher in the intervention group than in the control group. After 12 months, the costs were €1,796
265 higher. These differences were largely due to programme costs and costs of homecare.

266 When adopting a societal perspective, 6-month costs were €662 higher in the intervention group than in
267 the control group, whereas 12-month costs were €2,136 higher. Costs of informal care were slightly
268 lower in the intervention group than in the control group after 6 months, but higher after 12 months.

For peer review only

For peer review only - http://bmjopen.bmj.com/site/about/guidelines.xhtml

269 **Table 4.** Mean costs (€) after 6- and 12-months of follow-up (after IPW)

	**6 months**			**12 months**		
**Cost category**	**Mean costs (SD) CCFE (n=172)**	**Mean costs (SD) UC (n=129)**	**Difference between means (SE)**	**Mean costs (SD) CCFE (n=156)**	**Mean costs (SD) UC (n=113)**	**Difference between means (SE)**
Chronic care programme(s) #	534 (-)	72 (-)	462 (-)	1,068 (-)	143 (-)	925 (-)
Homecare	3,289 (4,371)	3,158 (4,016)	131 (481)	7,597 (8474)	6,330 (7680)	1,267 (980)
Long-term care admissions	969 (5,411)	766 (4,980)	203 (589)	1,624 (7,959)	2,051 (10,025)	-427 (11,33)
Hospital admissions	670 (2,670)	485 (1,833)	185 (259)	1,199 (3,603)	1,119 (2,992)	80 (403)
Emergency room visits	112 (308)	116 (341)	-4 (38)	179 (372)	196 (418)	-17 (49)
Outpatient day-care activities	235 (1,180)	310 (1,481)	-74 (170)	627 (2,642)	408 (1,846)	218 (285)
Medical specialist care	338 (619)	317 (408)	21 (60)	631 (789)	609 (615)	23 (86)
Paramedical care (e.g., physiotherapist)	529 (636)	481 (548)	48 (72)	1,059 (1,113)	1,005 (1,133)	55 (145)
General practitioner ~	21 (27)	288 (272)	-267 (28)	25 (34)	492 (394)	-467 (44)
Medication	363 (662)	318 (551)	45 (65)	744 (1,232)	605 (927)	139 (124)
**Subtotal costs from healthcare perspective**	**7 060 (8,441)**	**6,310 (7,671)**	**751 (922)**	**14,753 (14,498)**	**12,957 (14,185)**	**1,796 (1,777)**
Informal care	3,690 (5,780)	3,779 (7,318)	-89 (891)	8,063 (12,436)	7,723 (13,643)	340 (1,783)
**Total costs from societal perspective**	**10,750 (10,381)**	**10,089 (11,003)**	**662 (1,309)**	**22,816 (19,050)**	**20,680 (20,251)**	**2,136 (2,551)**

270 *# Costs for chronic care program(s) is an average estimation which is the same for each respondent in their respective group; ~ Costs for GP-care in the intervention*
271 *group are largely included in the costs of the chronic care program. SD=Standard deviation. SE=Standard Error. CCFE=Care Chain Frail Elderly. UC=Usual care.*

For peer review only - http://bmjopen.bmj.com/site/about/guidelines.xhtml

Multi-Criteria Decision Analysis

272 273 **Table 5a and b** present the standardised outcome scores for the CCFE and usual care as well as these scores weighted according to each stakeholder's importance-weights, at 6- and 12-months, respectively. The overall value scores show that all stakeholder groups preferred the CCFE over usual care at 6 months follow-up. This was driven by the performance scores of person-centeredness and enjoyment of life, and the high importance-weight of the latter. In more than 75% of iterations the CCFE had a higher overall value score than usual care. At 12 months follow-up the difference disappeared, and the probability that the CCFE had a higher overall value score dropped below 50% for payers and policymakers. This was driven by worse scores in physical functioning and costs.

For peer review only

For peer review only - http://bmjopen.bmj.com/site/about/guidelines.xhtml

Table 5a. Value scores in the Multi-Criteria Decision Analysis at 6 months, using DCE weights

			Patients		Partners		Professionals		Payers		Policymakers	
**Outcome measures**	**Standardised performance score[#]**		**Weighted score**		**Weighted score**		**Weighted score**		**Weighted score**		**Weighted score**	
	**CCFE**	**UC**	**CCFE**	**UC**	**CCFE**	**UC**	**CCFE**	**UC**	**CCFE**	**UC**	**CCFE**	**UC**
Physical functioning	0.672	0.740	0.108	0.118	0.074	0.081	0.081	0.089	0.094	0.104	0.094	0.104
Psychological well-being	0.706	0.709	0.120	0.120	0.106	0.106	0.127	0.128	0.127	0.128	0.106	0.106
Enjoyment of life	0.729	0.685	0.168	0.157	0.182	0.171	0.160	0.151	0.175	0.164	0.160	0.151
Social relationships and participation	0.718	0.696	0.057	0.056	0.065	0.063	0.079	0.077	0.072	0.070	0.072	0.070
Resilience	0.711	0.704	0.107	0.106	0.099	0.099	0.092	0.091	0.078	0.077	0.099	0.099
Person-centeredness	0.749	0.663	0.060	0.053	0.060	0.053	0.060	0.053	0.045	0.040	0.060	0.053
Continuity of care	0.718	0.696	0.072	0.070	0.086	0.083	0.079	0.077	0.057	0.056	0.072	0.070
Costs (societal care perspective)	0.685	0.729	0.021	0.022	0.041	0.044	0.041	0.044	0.055	0.058	0.048	0.051
**Overall value scores**			**0.711**	**0.702**	**0.713**	**0.700**	**0.719**	**0.708**	**0.703**	**0.696**	**0.711**	**0.702**
**95% Confidence interval^**			0.702 - 0.721	0.692 - 0.712	0.703 - 0.723	0.690 - 0.710	0.702 - 0.722	0.691 - 0.711	0.699 - 0.721	0.692 - 0.714	0.700 - 0.721	0.692 - 0.712
**% CCFE > UC^**			82%		90%		87%		75%		81%	

For peer review only - http://bmjopen.bmj.com/site/about/guidelines.xhtml

Table 5b. Value scores in the Multi-Criteria Decision Analysis at 12 months, using DCE weights

			Patients		Partners		Professionals		Payers		Policymakers	
**Outcome measures**	**Standardised performance score#**		**Weighted score**		**Weighted score**		**Weighted score**		**Weighted score**		**Weighted score**	
	**CCFE**	**UC**	**CCFE**	**UC**	**CCFE**	**UC**	**CCFE**	**UC**	**CCFE**	**UC**	**CCFE**	**UC**
Physical functioning	0.682	0.731	0.109	0.117	0.075	0.080	0.082	0.088	0.095	0.102	0.095	0.102
Psychological well-being	0.701	0.713	0.119	0.121	0.105	0.107	0.126	0.128	0.126	0.128	0.105	0.107
Enjoyment of life	0.711	0.703	0.164	0.162	0.178	0.176	0.157	0.155	0.171	0.169	0.157	0.155
Social relationships and participation	0.720	0.694	0.058	0.056	0.065	0.062	0.079	0.076	0.072	0.069	0.072	0.069
Resilience	0.706	0.709	0.106	0.106	0.099	0.099	0.092	0.092	0.078	0.078	0.099	0.099
Person-centeredness	0.744	0.668	0.060	0.053	0.060	0.053	0.060	0.053	0.045	0.040	0.060	0.053
Continuity of care	0.719	0.695	0.072	0.069	0.086	0.083	0.079	0.076	0.058	0.056	0.072	0.069
Costs (societal care perspective)	0.673	0.739	0.020	0.022	0.040	0.044	0.040	0.044	0.054	0.059	0.047	0.052
**Overall value scores**			**0.707**	**0.707**	**0.708**	**0.706**	**0.714**	**0.713**	**0.698**	**0.702**	**0.706**	**0.707**
**95% Confidence interval^**			0.696 - 0.717	0.697 - 0.717	0.697 - 0.718	0.695 - 0.716	0.697 - 0.717	0.695 - 0.716	0.695 - 0.717	0.697 - 0.719	0.695 - 0.717	0.696 - 0.718
**% CCFE > UC^**			50%		57%		54%		39%		48%	

^ based on Monte Carlo simulation; # colour scheme ranges from red (lowest score) to green (highest score). CCFE=Care Chain Frail Elderly. UC=Usual care.

For peer review only - http://bmjopen.bmj.com/site/about/guidelines.xhtml

DISCUSSION

Main findings

The MCDA has shown that all stakeholders preferred the CCFE over usual care at 6 months with a likelihood of having a higher overall value score of over 75%. This was mainly driven by higher performance on enjoyment of life and person-centeredness, and the great importance of the former outcome. Results became more diffuse at 12 months. Patients were indifferent, informal caregivers, and professionals slightly favoured the CCFE, whereas payers and policymakers demonstrated a slight preference for usual care. This was mainly due to a worse performance of the CCFE on physical functioning and costs. When looking at the disaggregated scores, person-centeredness was consistently higher in the CCFE. Physical functioning deteriorated in the intervention group at 6 months, but this effect disappeared at 12 months. When some outcomes improve whereas others deteriorate, the current MCDA approach is a suitable method to aggregate them into overall value scores that vary depending on the importance that stakeholders assign to the different outcome measures. These results show that the CCFE is the preferred way of delivering care to frail elderly if improvements in enjoyment of life and person-centeredness are considered more important than physical functioning and costs.

Context and comparison with previous evaluations

As this is the first MCDA of a frail elderly program, it is impossible to directly compare the value scores to other studies. MCDA also provides insight in the disaggregated effects of the CCFE, but these are hard to compare to other studies as well, due to the very frail target group of the CCFE, the different contexts in which the interventions are implemented, the different intervention components, and outcome measures[38,39] Regarding the context of the CCFE, we should stress that the programme was implemented in a setting with a strong primary care sector. GPs already have a

For peer review only - http://bmjopen.bmj.com/site/about/guidelines.xhtml

history of collaborating with other primary care providers, for example by working jointly in health centres. Hence, setting up community networks and collaborating in wider multi-disciplinary teams was not such a big step. When implementing similar interventions in countries with a less strong primary care system, collaboration may require more efforts. On the other hand, the potential for savings due to the intervention might be higher in countries where the GPs does not act as a gatekeeper to secondary care, as a programme like the CCFE could substitute more secondary care services by primary care services.

Despite differences between studies, most previous studies did not find effects on physical functioning.[8,40] This may be expected as these programmes rarely aim to achieve improvements in this domain. As the CCFE aimed to improve experience with care, the sustained improvement in person-centeredness found in this study suggests that the programme has fulfilled that aim.[1] The worsening in autonomy and burden of medication seems counterintuitive. A possible explanation for the deterioration in autonomy is that as elderly in the CCFE were confronted with their frailty, e.g., by discussing their needs for support, they became more aware of their loss of control. This could have led to lower autonomy scores in the intervention group, especially due to the self-report method; measurement by a third party might have led to different scores.[41] The higher medication burden in the intervention group could be explained by alterations in medication after the medication review, which may have led to (temporary) side-effects.

One could question the relevance of finding a sustained effect on an outcome (i.e., person-centredness) that was less highly valued than other outcomes in the DCE. This may raise the question whether the aim of the CCFE was well-targeted. However, weights were derived from a DCE that asks respondents to choose between two hypothetical care programmes, which gives them the opportunity to trade-off person-centredness for, for example, improved physical functioning. Even though this is likely to be an appropriate reflection of their preferences if all options were open, in real life this trade-off may no longer exist, because improving physical functioning might not

For peer review only - http://bmjopen.bmj.com/site/about/guidelines.xhtml

be possible anymore. Hence, there is a discrepancy between what is important to a patient and what is feasible in practice.

Strengths & limitations

One of the strengths of our study was its controlled study design. Defining an appropriate control group to evaluate ongoing programmes for frail elderly is a challenge.[8,42] However, it is especially important in evaluating frail elderly care, as there is commonly little room for actual improvements in health, and prevention or delay of deterioration can only be shown in comparison with a control group.

Another strength was the data collection on a broad range of PROMs and PREMs by interviewers who made a total of 954 home-visits, which was a major endeavour. Collecting patient-reported data did limit the generalisability of the results, as some frail elderly could not participate in the evaluation because that was too burdensome or impossible. Hence, the frailest among the elderly were not represented in this study, especially not those with dementia. The attrition rate in our study was relatively low, i.e., 30% across both groups at 12 months. We did observe that respondents in the control group that were lost to follow-up were slightly older and had worse physical functioning at baseline compared to non-dropouts in the control group (and overall), see **Appendix 5**. This may have led to an underestimation of the treatment effect.

A further strength of our study was the detailed cost-analysis, even including costs of medication, social care and informal care, which are often excluded from other studies.[8] This analysis showed a cost increase reflecting the greater investment of resources to support frail elderly in ageing in place which is of great importance to many older persons.

In the design and reporting on the MCDA we followed the good practice guidelines as laid out by the ISPOR MCDA-taskforce.[43] Strengths of MCDA are that it enables explicit, transparent and accountable decision-making, i.e., for every decision what was valued most and by whom can be

For peer review only

tracked down as well as whether this was due to improvements in certain domains or a higher relative importance of a particular outcome. Furthermore, MCDA makes it possible to include additional elements of value that go beyond health or QALYs, which is especially important for complex interventions with multiple aims such as improving well-being and experience with care. However, the consequence is that we may favour interventions that achieve improvements in these outcomes above interventions that have greater health outcomes. This may be justifiable for elderly care. Such an argument would raise another point that is debated in MCDA, namely whether or not to include costs in the overall value score.[44,45] To elicit a weight for costs, stakeholders had to trade-off costs against other outcomes, which makes the relative contribution of costs to the overall value score explicit. However, it can be argued that this does not adequately address the opportunity costs of the CCFE.[46] We also performed the MCDA without costs as a sensitivity analysis, see **Appendix 6**, which led to higher overall value scores for the CCFE at both time points. However, now the overall value forms a composite benefit score for which a new cost-effectiveness threshold must be determined. Although we believe the current set of outcomes captures the full potential value of an integrated care programme for frail elderly, this set should be tailored to each intervention's aims and target group. Therefore, when the set of outcome measures changes, new thresholds need to be determined. Another option is to calculate the cost-per-value and prioritise interventions with the lowest cost-per-value ratio, but this only leads to a ranking of interventions.[45] A last point of discussion on MCDA is that it requires a deliberative component to avoid making decisions based solely on the model.[45] In our study we presented results from five stakeholder perspectives which inevitably calls for further deliberation to determine which perspective should prevail.

Implications

Although the CCFE does not improve the (physical) health of patients, it is still positively evaluated by all stakeholder groups at 6 months. At 12 months stakeholders were mainly indifferent. This warrants further research into interventions to maintain the effects of such programmes in the long-

For peer review only - http://bmjopen.bmj.com/site/about/guidelines.xhtml

term. Furthermore, we advocate a wider use of MCDA to evaluate multi-faceted, person-centred, integrated care programmes for frail elderly that aim to improve multiple outcomes, including those that go beyond health. MCDA enables a transparent and explicit decision-making process and serves as a tool to help decision-makers reach a decision. Therefore, MCDA-results are a good starting point for deliberation before deciding upon reimbursement or broader implementation of new interventions.

CONCLUSION

After 6 months, the overall value score for the CCFE was higher than for usual care across all stakeholders, but at 12 months, the preference for the CCFE had disappeared. The CCFE led to sustained improvements in enjoyment of life and person-centeredness, which is aligned with the program's aim, but also to a deterioration in physical functioning at 6 months and higher costs. Therefore, the CCFE is only the preferred way of delivering care to frail elderly in case improvements in enjoyment of life and person-centeredness are considered more important than costs and physical functioning.

For peer review only

For peer review only - http://bmjopen.bmj.com/site/about/guidelines.xhtml

ACKNOWLEDGEMENTS

We would like to thank all respondents and their informal caregivers for participating in this research. We would also like to thank the care groups DOH, SGE and PoZoB for their continuous support in facilitating this research.

AUTHOR CONTRIBUTIONS

MH, MK, FL, AT, MRvM developed the overall methodology for the value-based MCDA approach in the SELFIE project. MH, MK, FL, MRvM contributed to the study design and data collection. MH, MK, LG, KI, AT, MRvM had access to the data and contributed to the data analysis. MH and MRvM drafted this manuscript. All co-authors critically reviewed this manuscript. All authors read and approved the final manuscript. MH and MRvM had full access to all of the data in the study and the final responsibility to submit for publication.

DECLARATION OF INTERESTS

The authors have no competing interests.

FUNDING

The SELFIE project has received funding from the European Union's Horizon 2020 research and innovation programme under grant agreement No 634288. The content of this manuscript reflects only the SELFIE group's views and the European Commission is not liable for any use that may be made of the information contained herein.

DATA SHARING AGREEMENT

The data that support the findings of this study are available on request from the corresponding author, MH. The data are not publicly available due to privacy restrictions.

For peer review only

For peer review only - http://bmjopen.bmj.com/site/about/guidelines.xhtml

REFERENCES

1. Hoedemakers M, Leijten FRM, Looman W, Czypionka T, Kraus M, Donkers H, et al. Integrated Care for Frail Elderly: A Qualitative Study of a Promising Approach in The Netherlands. International Journal of Integrated Care. 2019 Sep 3;19(3):16.
2. Maarse JAM, Jeurissen PP. The policy and politics of the 2015 long-term care reform in the Netherlands. Health Policy. 2016 Mar 1;120(3):241–5.
3. Alders P, Schut FT. The 2015 long-term care reform in the Netherlands: Getting the financial incentives right? Health Policy. 2019 Mar 1;123(3):312–6.
4. Wehrens R, Oldenhof L, Verweij L, Francke A, Bal R. Experimenteel sturen in netwerken: een evaluatie van proces en structuur van het Nationaal Programma Ouderenzorg. :99.
5. de Jong B, Wynia K, Geluk-Bleumink A. Ageing Better in the Netherlands. In D'Onofrio G, Sancarlo D, Greco A, editors, Gerontology. London: IntechOpen. 2018. p. 101-111
6. Briggs AM, Valentijn PP, Thiyagarajan JA, Carvalho IA de. Elements of integrated care approaches for older people: a review of reviews. BMJ Open. 2018 Apr 1;8(4):e021194.
7. Hendry A, Vanhecke E, Carriazo A, López-Samaniego L, Espinosa J, Sezgin D, et al. Integrated Care Models for Managing and Preventing Frailty: A Systematic Review for the European Joint Action on Frailty Prevention (ADVANTAGE JA). Transl Med UniSa. 2019 Jan 6;19:5–10.
8. Looman WM, Huijsman R, Fabbricotti IN. The (cost-)effectiveness of preventive, integrated care for community-dwelling frail older people: A systematic review. Health & Social Care in the Community. 2019;27(1):1–30.
9. Van der Elst M, Schoenmakers B, Duppen D, Lambotte D, Fret B, Vaes B, et al. Interventions for frail community-dwelling older adults have no significant effect on adverse outcomes: a systematic review and meta-analysis. BMC Geriatrics. 2018 Oct 20;18(1):249.

For peer review only - http://bmjopen.bmj.com/site/about/guidelines.xhtml

10. Hopman P, de Bruin SR, Forjaz MJ, Rodriguez-Blazquez C, Tonnara G, Lemmens LC, et al. Effectiveness of comprehensive care programmes for patients with multiple chronic conditions or frailty: A systematic literature review. Health Policy. 2016 Jul 1;120(7):818–32.

11. Vorst A van der, Zijlstra GAR, Witte ND, Duppen D, Stuck AE, Kempen GIJM, et al. Limitations in Activities of Daily Living in Community-Dwelling People Aged 75 and Over: A Systematic Literature Review of Risk and Protective Factors. PLOS ONE. 2016 Oct 19;11(10):e0165127.

12. Makai P. Moving beyond the QALY: economic evaluation in health and social care for elderly populations. [Rotterdam]: Erasmus Universiteit Rotterdam; 2014.

13. Cramm J, Nieboer A. Is ”disease management” the answer to our problems? No! Population health management and (disease) prevention require ”management of overall well-being” BMC Health Services Research. 2016 Sep 21;16.

14. Makai P, Brouwer WBF, Koopmanschap MA, Stolk EA, Nieboer AP. Quality of life instruments for economic evaluations in health and social care for older people: A systematic review. Social Science & Medicine. 2014 Feb 1;102:83–93.

15. Berwick DM, Nolan TW, Whittington J. The Triple Aim: Care, Health, And Cost. Health Affairs. 2008 May 1;27(3):759–69.

16. Rutten-van Mölken M, Leijten F, Hoedemakers M, Tsiachristas A, Verbeek N, Karimi M, et al. Strengthening the evidence-base of integrated care for people with multi-morbidity in Europe using Multi-Criteria Decision Analysis (MCDA). BMC Health Services Research. 2018 Jul 24;18(1):576.

17. Leijten FRM, Hoedemakers M, Struckmann V, Kraus M, Cheraghi-Sohi S, Zemplényi A, et al. Defining good health and care from the perspective of persons with multimorbidity: results from a qualitative study of focus groups in eight European countries. BMJ Open. 2018 Aug 1;8(8):e021072.

For peer review only - http://bmjopen.bmj.com/site/about/guidelines.xhtml

18. Laan W, Zuithoff NPA, Drubbel I, Bleijenberg N, Numans ME, De Wit NJ, et al. Validity and reliability of the Katz-15 scale to measure unfavorable health outcomes in community-dwelling older people. J Nutr Health Aging. 2014 Nov 1;18(9):848–54.

19. Rumpf H-J, Meyer C, Hapke U, John U. Screening for mental health: validity of the MHI-5 using DSM-IV Axis I psychiatric disorders as gold standard. Psychiatry Research. 2001 Dec 31;105(3):243–53.

20. Coast J, Peters TJ, Natarajan L, Sproston K, Flynn T. An assessment of the construct validity of the descriptive system for the ICECAP capability measure for older people. Qual Life Res. 2008; 17(7): 967-76.

21. Cardol M, de Haan RJ, van den Bos GA, de Jong BA, de Groot IJ. The development of a handicap assessment questionnaire: the Impact on Participation and Autonomy (IPA). Clin Rehabil. 1999; 13(5): 411-9.

22. Smith BW, Dalen J, Wiggins K, Tooley E, Christopher P, Bernard J. The brief resilience scale: assessing the ability to bounce back. Int J Behav Med. 2008; 15(3): 194-200.

23. Lloyd H, Fosh B, Whalley B, Byng R, Close J. Validation of the person-centred coordinated care experience questionnaire (P3CEQ). International Journal for Quality in Health Care. 2019 Aug 1;31(7):506–12.

24. Uijen AA, Schellevis FG, van den Bosch WJ, Mokkink HG, van Weel C, Schers HJ. Nijmegen Continuity Questionnaire: development and testing of a questionnaire that measures continuity of care. J Clin Epidemiol. 2011; 64(12): 1391-9.

25. McGuiness C, Sibthorpe B. Development and initial validation of a measure of coordination of health care. Int J Qual Health Care. 2003; 15(4): 309-18.

26. Bouwmans C, Hakkaart-van Roijen L, Koopmanschap M, Krol M, Severens H, Brouwer W. Handleiding iMTA Medical Cost Questionnaire (iMCQ). Rotterdam: iMTA: Erasmus Universiteit; 2013.

For peer review only

27. Pearlin LI, Schooler C. The Structure of Coping. Journal of Health and Social Behavior. 1978;19(1):2–21.

28. Krska J, Morecroft CW, Rowe PH, Poole H. Measuring the impact of long-term medicines use from the patient perspective. Int J Clin Pharm. 2014 Aug 1;36(4):675–8.

29. Kanters TA, Bouwmans CAM, Linden N van der, Tan SS, Roijen LH. Update of the Dutch manual for costing studies in health care. PLoS ONE. 2017 Nov 9;12(11):e0187477.

30. G-Standaard Flex — Z-Index [Internet]. [cited 2021 Feb 2]. Available from: https://www.z-index.nl/producten-diensten/g-standaard-flex.

31. Vektis - business intelligence centrum voor de zorg | Vektis.nl [Internet]. Vektis. [cited 2021 Feb 2]. Available from: https://www.vektis.nl.

32. Rosenbaum PR, Rubin DB. The Central Role of the Propensity Score in Observational Studies for Causal Effects. Biometrika. 1983;70(1):41–55.

33. Austin PC, Stuart EA. Moving towards best practice when using inverse probability of treatment weighting (IPTW) using the propensity score to estimate causal treatment effects in observational studies. Stat Med. 2015 Dec 10;34(28):3661–79.

34. Rubin DB. Using Propensity Scores to Help Design Observational Studies: Application to the Tobacco Litigation. Health Services & Outcomes Research Methodology. 2001 Dec 1;2(3):169–88.

35. Stuart EA. Matching methods for causal inference: A review and a look forward. Stat Sci. 2010 Feb 1;25(1):1–21.

36. Mölken MR, Karimi M, Leijten F, Hoedemakers M, Looman W, Islam K, et al. Comparing patients' and other stakeholders' preferences for outcomes of integrated care for multimorbidity: a discrete choice experiment in eight European countries. BMJ Open. 2020 Oct 1;10(10):e037547.

37. Publications [Internet]. Selfie 2020 -. [cited 2021 Feb 1]. Available from: https://www.selfie2020.eu/publications/.

For peer review only

38. Baxter S, Johnson M, Chambers D, Sutton A, Goyder E, Booth A. The effects of integrated care: a systematic review of UK and international evidence. BMC Health Serv Res. 2018 May 10;18(1):350.

39. Wodchis WP, Dixon A, Anderson GM, Goodwin N. Integrating care for older people with complex needs: key insights and lessons from a seven-country cross-case analysis. International Journal of Integrated Care. 2015;15(6).

40. Blom JW, Van den Hout WB, Den Elzen WPJ, Drewes YM, Bleijenberg N, Fabbricotti IN, et al. Effectiveness and cost-effectiveness of proactive and multidisciplinary integrated care for older people with complex problems in general practice: an individual participant data meta-analysis. Age and Ageing. 2018 Sep 1;47(5):705–14.

41. Bauhoff S. Systematic self-report bias in health data: impact on estimating cross-sectional and treatment effects. Health Serv Outcomes Res Method. 2011 Jul 1;11(1):44–53.

42. Liljas AEM, Brattström F, Burström B, Schön P, Agerholm J. Impact of Integrated Care on Patient-Related Outcomes Among Older People – A Systematic Review. International Journal of Integrated Care. 2019 Jul 24;19(3):6.

43. Marsh K, IJzerman M, Thokala P, Baltussen R, Boysen M, Kaló Z, et al. Multiple Criteria Decision Analysis for Health Care Decision Making—Emerging Good Practices: Report 2 of the ISPOR MCDA Emerging Good Practices Task Force. Value in Health. 2016 Mar 1;19(2):125–37.

44. Marsh KD, Sculpher M, Caro JJ, Tervonen T. The Use of MCDA in HTA: Great Potential, but More Effort Needed. Value in Health. 2018 Apr 1;21(4):394–7.

45. Baltussen R, Marsh K, Thokala P, Diaby V, Castro H, Cleemput I, et al. Multicriteria Decision Analysis to Support Health Technology Assessment Agencies: Benefits, Limitations, and the Way Forward. Value in Health. 2019 Nov 1;22(11):1283–8.

46. Sculpher M, Claxton K, Pearson SD. Developing a Value Framework: The Need to Reflect the Opportunity Costs of Funding Decisions. Value in Health. 2017 Feb;20(2):234–9.

For peer review only - http://bmjopen.bmj.com/site/about/guidelines.xhtml

Figure caption

Figure 1. Flow chart of patient participation

For peer review only

For peer review only - http://bmjopen.bmj.com/site/about/guidelines.xhtml

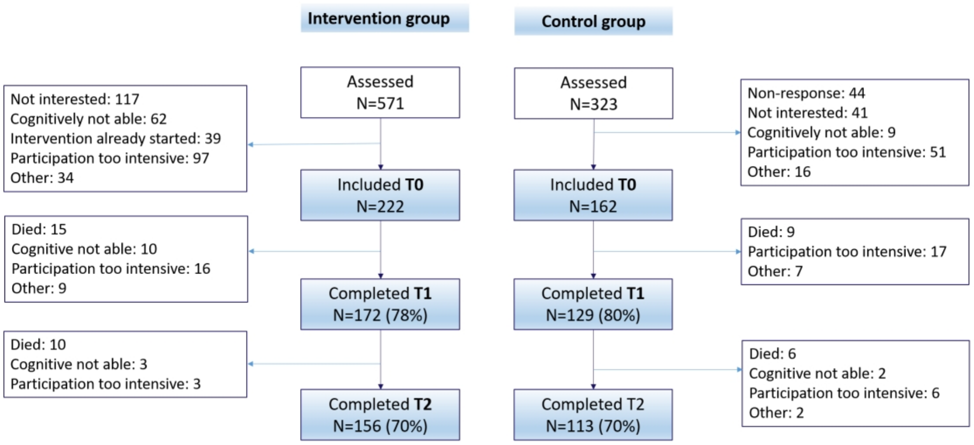

Figure 1. Flowchart of patient participation

985x471mm (59 x 59 DPI)

Supplementary files

Appendix 1: Equation relative standardization 2

Appendix 2. Relative DCE weights (0-1) (SE) of the outcomes used in the MCDA by type of stakeholder 3

Appendix 3: Graph common support 4

Appendix 4: Predicted mean performance scores of all outcome measures on their natural scale...... 5

Appendix 5: Comparison of baseline characteristics and outcome scores of dropouts versus non-dropouts.......... 6

Appendix 6a: Value scores in the Multi-Criteria Decision Analysis at 6 months without costs 8

Appendix 6b: Value scores in the Multi-Criteria Decision Analysis at 12 months without costs 9

For peer review only

For peer review only - http://bmjopen.bmj.com/site/about/guidelines.xhtml

Appendix 1: Equation relative standardization

$$S_{aj} = \frac{x_{aj}}{\left(x_{aj}^2 + x_{bj}^2\right)^{1/2}}$$

Where

x = predicted mean score on the natural scale

a = the intervention group

b = the control group

j = outcome measure *j*

Note: For all outcomes in the MCDA, a higher score indicates better performance. To achieve this, in the above-mentioned equation, x is replaced by $1/x$ for outcomes where a higher score on the natural scale indicates a worse performance (e.g., costs).

For peer review only - http://bmjopen.bmj.com/site/about/guidelines.xhtml

Appendix 2. Relative DCE weights (0-1) (SE) of the outcomes used in the MCDA by type of stakeholder

	**Patients (n=156)**	**Partners (n=158)**	**Professionals (n=155)**	**Payers (n=104)**	**Policymakers (n=151)**
Physical functioning	0.16 (0.15)	0.11 (0.12)	0.12 (0.12)	0.14 (0.13)	0.14 (0.14)
Psychological well-being	0.17 (0.15)	0.15 (0.14)	0.18 (0.16)	0.18 (0.17)	0.15 (0.15)
Enjoyment of life	0.23 (0.18)	0.25 (0.19)	0.22 (0.19)	0.24 (0.23)	0.22 (0.19)
Social participation & relationships	0.08 (0.10)	0.09 (0.11)	0.11 (0.11)	0.10 (0.11)	0.10 (0.10)
Resilience	0.15 (0.13)	0.14 (0.13)	0.13 (0.12)	0.11 (0.10)	0.14 (0.13)
Person-centeredness	0.08 (0.10)	0.08 (0.10)	0.08 (0.09)	0.06 (0.08)	0.08 (0.09)
Continuity of care	0.10 (0.11)	0.12 (0.12)	0.11 (0.11)	0.08 (0.09)	0.10 (0.10)
Total health and social care costs	0.03 (0.08)	0.06 (0.09)	0.06 (0.09)	0.08 (0.08)	0.07 (0.09)

Note: Numbers in parentheses (n) by stakeholders indicate the number of participants included in the weight elicitation study

For peer review only

For peer review only - http://bmjopen.bmj.com/site/about/guidelines.xhtml

Appendix 3: Graph common support

Graph 1: Common Support of the propensity scores when matching includes the baseline values of the following variables: age, gender, marital status, living situation, educational level, smoking status, and total health and social care costs (minus medication) of three months prior to baseline as a proxy for severity

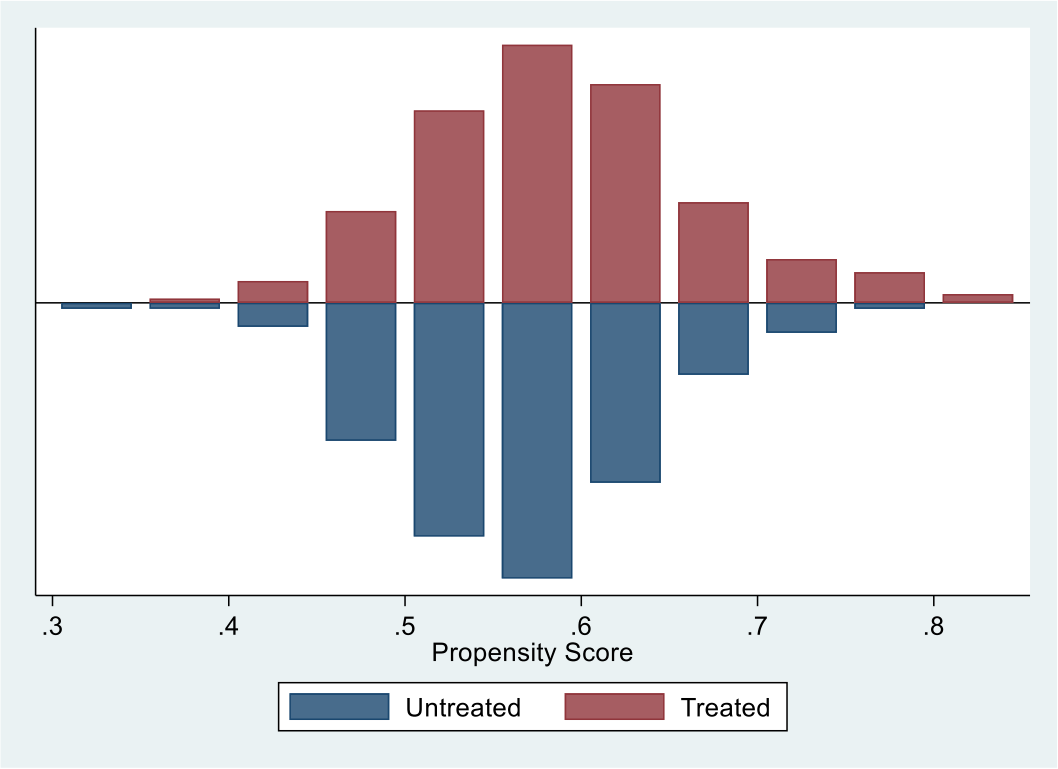

For peer review only - http://bmjopen.bmj.com/site/about/guidelines.xhtml

Appendix 4: Predicted mean performance scores of all outcome measures on their natural scale

	T0#		T1		T2	
**Outcomes measures**	**CCFE (n=222)**	**UC (n=162)**	**CCFE (n=172)**	**UC (n=129)**	**CCFE (n=156)**	**UC (n=113)**
**Health & Well-being**						
Physical functioning (0-15) ^	4.383	4.383	5.126	4.653	5.715	5.328
Psychological well-being (0-100)	71.351	71.351	70.707	71.010	69.998	71.256
Enjoyment of life (1-4)	2.820	2.820	2.917	2.741	2.874	2.839
Social relationships & participation (7-35) ^	9.167	9.167	9.273	9.579	9.424	9.779
Resilience (6-30)	19.317	19.317	19.299	19.108	19.347	19.426
**Experience of care**						
Person-centeredness (0-18)	11.729	11.729	12.828	11.353	13.060	11.727
Continuity of care (1-5)	3.672	3.672	3.771	3.653	3.833	3.704
**Costs**						
Total health and social care costs ^	5453	5453	10858	10199	22676	20659
**Additional frail elderly-specific outcomes**						
Autonomy (7-35)	22.284	22.284	21.785	22.306	21.790	22.834
Burden of medication (0-10) ^	2.088	2.088	2.221	1.878	2.263	1.480

# Note that the intervention coefficient was included in the calculation of the mean performance scores in the control group, assuming the control group had the same baseline scores as the intervention group to make it possible to directly compare the intervention and control group. CCFE=Care Chain Frail Elderly. UC=Usual care.

For peer review only - http://bmjopen.bmj.com/site/about/guidelines.xhtml

Appendix 5: Comparison of baseline characteristics and outcome scores of dropouts versus non-dropouts

	Across CCFE and UC			CCFE separately			UC separately		
**Baseline characteristics**	**Dropouts (n=115)**	**Non-dropouts (n=269)**	**P-value**	**Dropouts (n=66)**	**Non-dropouts (n=156)**	**P-value**	**Dropouts (n=49)**	**Non-dropouts (n=113)**	**P-value**
**Intervention group**	66 (57%)	156 (58%)	0.913	-	-	-	-	-	-
**Age**	85.00	83.56	0.036*	83.91	83.21	0.449	86.47	84.05	0.016*
**Gender** (female)	61%	65%	0.434	62%	64%	0.779	59%	66%	0.381
**Living situation**			0.926			0.842			0.711
Independent, alone	57%	57%		53%	54%		63%	60%	
With other(s)	43%	43%		47%	46%		37%	40%	
**Marital status**			0.162			0.149			0.734
Single, never married	1%	5%		0%	5%		2%	4%	
Married or living together	41%	44%		44%	45%		37%	42%	
Widow(er)	53%	45%		52%	42%		55%	49%	
Divorced	5%	7%		5%	8%		6%	4%	
**Educational level**			0.923			0.839			0.981
Low	70%	71%		68%	71%		71%	70%	
Middle	19%	17%		23%	19%		14%	15%	
High	11%	12%		9%	10%		14%	15%	
**Smoking** (yes)	15%	11%	0.269	18%	13%	0.299	10%	8%	0.641
**Baseline outcome scores**									
Physical functioning (0-15) ^	5.18	4.10	0.001*	4.89	4.17	0.081	5.56	4.02	0.003*
Psychological well-being (0-100)	69.37	72.39	0.145	68.06	72.74	0.092	71.17	71.89	0.815
Enjoyment of life (1-4)	2.81	2.89	0.351	2.74	2.85	0.349	2.90	2.95	0.725

For peer review only - http://bmjopen.bmj.com/site/about/guidelines.xhtml

	Across CCFE and UC			CCFE separately			UC separately		
**Baseline characteristics**	**Dropouts (n=115)**	**Non-dropouts (n=269)**	**P-value**	**Dropouts (n=66)**	**Non-dropouts (n=156)**	**P-value**	**Dropouts (n=49)**	**Non-dropouts (n=113)**	**P-value**
Social relationships and participation (7-35) ^	8.86	8.55	0.461	9.48	9.03	0.451	8.00	7.88	0.831
Resilience (6-30)	18.78	19.38	0.212	18.97	19.46	0.448	18.52	19.27	0.298
Autonomy (7-35)	21.81	22.35	0.245	21.89	22.45	0.386	21.69	22.21	0.452
Person-centeredness (0-18)	12.29	11.97	0.473	11.86	11.66	0.730	12.88	12.4	0.481
Continuity of care (1-5)	3.70	3.72	0.838	3.65	3.69	0.743	3.78	3.77	0.939
Burden of medication (0-10) ^	2.17	2.24	0.809	2.12	2.07	0.908	2.22	2.47	0.604
Total costs 3 months prior to the study (€) ^	5,989	5,112	0.247	5,797	5,308	0.635	6,248	4,842	0.208

*^ Higher score indicating worse performance. * p<0.05. CCFE=Care Chain Frail Elderly. UC=Usual care.*

For peer review only - http://bmjopen.bmj.com/site/about/guidelines.xhtml

Appendix 6a: Value scores in the Multi-Criteria Decision Analysis at 6 months without costs

			Patients		Partners		Professionals		Payers		Policymakers	
Outcome measures	Standardised performance score[#]		Weighted score		Weighted score		Weighted score		Weighted score		Weighted score	
	CCFE	UC	CCFE	UC	CCFE	UC	CCFE	UC	CCFE	UC	CCFE	UC
Physical functioning	0.672	0.740	0.111	0.122	0.079	0.087	0.085	0.094	0.103	0.114	0.101	0.111
Psychological well-being	0.706	0.709	0.124	0.124	0.113	0.113	0.134	0.134	0.140	0.140	0.114	0.114
Enjoyment of life	0.729	0.685	0.173	0.162	0.194	0.182	0.169	0.159	0.192	0.181	0.172	0.162
Social relationships and participation	0.718	0.696	0.059	0.057	0.069	0.067	0.083	0.081	0.079	0.076	0.077	0.075
Resilience	0.711	0.704	0.110	0.109	0.106	0.105	0.097	0.096	0.086	0.085	0.107	0.106
Person-centeredness	0.749	0.663	0.062	0.055	0.064	0.056	0.063	0.056	0.049	0.044	0.064	0.057
Continuity of care	0.718	0.696	0.074	0.072	0.092	0.089	0.083	0.081	0.063	0.061	0.077	0.075
**Overall value scores**			**0.712**	**0.701**	**0.715**	**0.698**	**0.714**	**0.700**	**0.713**	**0.701**	**0.713**	**0.700**

[#] *colour scheme ranges from red (lowest score) to green (highest score). CCFE=Care Chain Frail Elderly. UC=Usual care.*

For peer review only - http://bmjopen.bmj.com/site/about/guidelines.xhtml

Appendix 6b: Value scores in the Multi-Criteria Decision Analysis at 12 months without costs

			Patients		Partners		Professionals		Payers		Policymakers	
Outcome measures	Standardised performance score#		Weighted score		Weighted score		Weighted score		Weighted score		Weighted score	
	CCFE	UC	CCFE	UC	CCFE	UC	CCFE	UC	CCFE	UC	CCFE	UC
Physical functioning	0.682	0.731	0.112	0.121	0.080	0.086	0.086	0.092	0.105	0.113	0.103	0.110
Psychological well-being	0.701	0.713	0.123	0.125	0.112	0.114	0.133	0.135	0.139	0.141	0.113	0.115
Enjoyment of life	0.711	0.703	0.169	0.167	0.189	0.187	0.165	0.163	0.188	0.185	0.168	0.166
Social relationships and participation	0.720	0.694	0.059	0.057	0.069	0.066	0.083	0.080	0.079	0.076	0.077	0.075
Resilience	0.706	0.709	0.109	0.110	0.105	0.106	0.097	0.097	0.085	0.086	0.106	0.107
Person-centeredness	0.744	0.668	0.061	0.055	0.063	0.057	0.063	0.056	0.049	0.044	0.064	0.057
Continuity of care	0.719	0.695	0.074	0.072	0.092	0.089	0.083	0.080	0.063	0.061	0.077	0.075
**Overall value scores**			**0.708**	**0.706**	**0.710**	**0.704**	**0.710**	**0.704**	**0.708**	**0.706**	**0.709**	**0.705**

colour scheme ranges from red (lowest score) to green (highest score). CCFE=Care Chain Frail Elderly. UC=Usual care.

For peer review only - http://bmjopen.bmj.com/site/about/guidelines.xhtml

The RECORD statement – checklist of items, extended from the STROBE statement, that should be reported in observational studies using routinely collected health data.

	Item No.	STROBE items	Location in manuscript where items are reported	RECORD items	Location in manuscript where items are reported
**Title and abstract**					
	1	(a) Indicate the study's design with a commonly used term in the title or the abstract (b) Provide in the abstract an informative and balanced summary of what was done and what was found	1,3	RECORD 1.1: The type of data used should be specified in the title or abstract. When possible, the name of the databases used should be included.<br><br>RECORD 1.2: If applicable, the geographic region and timeframe within which the study took place should be reported in the title or abstract.<br><br>RECORD 1.3: If linkage between databases was conducted for the study, this should be clearly stated in the title or abstract.	1,3
**Introduction**					
Background rationale	2	Explain the scientific background and rationale for the investigation being reported	5,6		
Objectives	3	State specific objectives, including any prespecified hypotheses	6		
**Methods**					
Study Design	4	Present key elements of study design early in the paper	7,8		
Setting	5	Describe the setting, locations, and relevant dates, including periods of recruitment, exposure, follow-up, and data collection	7-9		

For peer review only

Participants	6	*(a) Cohort study* - Give the eligibility criteria, and the sources and methods of selection of participants. Describe methods of follow-up<br>*Case-control study* - Give the eligibility criteria, and the sources and methods of case ascertainment and control selection. Give the rationale for the choice of cases and controls<br>*Cross-sectional study* - Give the eligibility criteria, and the sources and methods of selection of participants<br><br>*(b) Cohort study* - For matched studies, give matching criteria and number of exposed and unexposed<br>*Case-control study* - For matched studies, give matching criteria and the number of controls per case	8-9<br><br>10	RECORD 6.1: The methods of study population selection (such as codes or algorithms used to identify subjects) should be listed in detail. If this is not possible, an explanation should be provided.<br><br>RECORD 6.2: Any validation studies of the codes or algorithms used to select the population should be referenced. If validation was conducted for this study and not published elsewhere, detailed methods and results should be provided.<br><br>RECORD 6.3: If the study involved linkage of databases, consider use of a flow diagram or other graphical display to demonstrate the data linkage process, including the number of individuals with linked data at each stage.	
Variables	7	Clearly define all outcomes, exposures, predictors, potential confounders, and effect modifiers. Give diagnostic criteria, if applicable.	9	RECORD 7.1: A complete list of codes and algorithms used to classify exposures, outcomes, confounders, and effect modifiers should be provided. If these cannot be reported, an explanation should be provided.	
Data sources/ measurement	8	For each variable of interest, give sources of data and details of methods of assessment (measurement).<br>Describe comparability of assessment methods if there is more than one group	9-10		

For peer review only

Bias	9	Describe any efforts to address potential sources of bias	10		
Study size	10	Explain how the study size was arrived at	see study protocol		
Quantitative variables	11	Explain how quantitative variables were handled in the analyses. If applicable, describe which groupings were chosen, and why	10		
Statistical methods	12	(a) Describe all statistical methods, including those used to control for confounding<br>(b) Describe any methods used to examine subgroups and interactions<br>(c) Explain how missing data were addressed<br>(d) *Cohort study* - If applicable, explain how loss to follow-up was addressed<br>*Case-control study* - If applicable, explain how matching of cases and controls was addressed<br>*Cross-sectional study* - If applicable, describe analytical methods taking account of sampling strategy<br>(e) Describe any sensitivity analyses	10-11<br>11		
Data access and cleaning methods		..		RECORD 12.1: Authors should describe the extent to which the investigators had access to the database population used to create the study population.	NA

For peer review only

				RECORD 12.2: Authors should provide information on the data cleaning methods used in the study.	NA
Linkage		..		RECORD 12.3: State whether the study included person-level, institutional-level, or other data linkage across two or more databases. The methods of linkage and methods of linkage quality evaluation should be provided.	NA
**Results**					
Participants	13	(a) Report the numbers of individuals at each stage of the study (*e.g.*, numbers potentially eligible, examined for eligibility, confirmed eligible, included in the study, completing follow-up, and analysed)<br>(b) Give reasons for non-participation at each stage.<br>(c) Consider use of a flow diagram	12	RECORD 13.1: Describe in detail the selection of the persons included in the study (*i.e.*, study population selection) including filtering based on data quality, data availability and linkage. The selection of included persons can be described in the text and/or by means of the study flow diagram.	13
Descriptive data	14	(a) Give characteristics of study participants (*e.g.*, demographic, clinical, social) and information on exposures and potential confounders<br>(b) Indicate the number of participants with missing data for each variable of interest<br>(c) *Cohort study* - summarise follow-up time (*e.g.*, average and total amount)	12<br>12<br>8		
Outcome data	15	*Cohort study* - Report numbers of outcome events or summary measures over time<br>*Case-control study* - Report numbers in each exposure	13		

For peer review only

		category, or summary measures of exposure<br>*Cross-sectional study* - Report numbers of outcome events or summary measures			
Main results	16	(a) Give unadjusted estimates and, if applicable, confounder-adjusted estimates and their precision (e.g., 95% confidence interval). Make clear which confounders were adjusted for and why they were included<br>(b) Report category boundaries when continuous variables were categorized<br>(c) If relevant, consider translating estimates of relative risk into absolute risk for a meaningful time period	13		
Other analyses	17	Report other analyses done—e.g., analyses of subgroups and interactions, and sensitivity analyses	13		
**Discussion**					
Key results	18	Summarise key results with reference to study objectives	14		
Limitations	19	Discuss limitations of the study, taking into account sources of potential bias or imprecision. Discuss both direction and magnitude of any potential bias	15	RECORD 19.1: Discuss the implications of using data that were not created or collected to answer the specific research question(s). Include discussion of misclassification bias, unmeasured confounding, missing data, and changing eligibility over time, as they pertain to the study being reported.	NA
Interpretation	20	Give a cautious overall interpretation of results considering objectives,	14,15		

For peer review only

		limitations, multiplicity of analyses, results from similar studies, and other relevant evidence			
Generalisability	21	Discuss the generalisability (external validity) of the study results	15		
**Other Information**					
Funding	22	Give the source of funding and the role of the funders for the present study and, if applicable, for the original study on which the present article is based	4		
Accessibility of protocol, raw data, and programming code		..		RECORD 22.1: Authors should provide information on how to access any supplemental information such as the study protocol, raw data, or programming code.	

*Reference: Benchimol EI, Smeeth L, Guttmann A, Harron K, Moher D, Petersen I, Sørensen HT, von Elm E, Langan SM, the RECORD Working Committee. The REporting of studies Conducted using Observational Routinely-collected health Data (RECORD) Statement. *PLoS Medicine* 2015; in press.

*Checklist is protected under Creative Commons Attribution (CC BY) license.

For peer review only

BMJ Open

Value-based person-centred integrated care for frail elderly living at home: a quasi-experimental evaluation using Multi-Criteria Decision Analysis

Journal:	*BMJ Open*
Manuscript ID	bmjopen-2021-054672.R2
Article Type:	Original research
Date Submitted by the Author:	02-Mar-2022
Complete List of Authors:	Hoedemakers, Maaike; Erasmus Universiteit Rotterdam, Erasmus School of Health Policy & Management<br>Karimi, Milad; Erasmus University Rotterdam; OPEN Health<br>Leijten, Fenna; Erasmus Universiteit Rotterdam, Health Policy & Management ; Ministry of defence, Staff Defence Healthcare Organisation<br>Goossens, Lucas; Erasmus University<br>Islam, Kamrul; University of Bergen, Department of Economics; NORCE Norwegian Research Centre AS, Social sciences<br>Tsiachristas, Apostolos; University of Oxford, Nuffield Department of Population Health<br>Rutten-van Molken, Maureen; Erasmus University Rotterdam; Erasmus University Rotterdam
**Primary Subject Heading**:	Health policy
Secondary Subject Heading:	Health services research, Health policy
Keywords:	Health economics < HEALTH SERVICES ADMINISTRATION & MANAGEMENT, HEALTH ECONOMICS, Health policy < HEALTH SERVICES ADMINISTRATION & MANAGEMENT, Organisation of health services < HEALTH SERVICES ADMINISTRATION & MANAGEMENT

SCHOLARONE™
Manuscripts

I, the Submitting Author has the right to grant and does grant on behalf of all authors of the Work (as defined in the below author licence), an exclusive licence and/or a non-exclusive licence for contributions from authors who are: i) UK Crown employees; ii) where BMJ has agreed a CC-BY licence shall apply, and/or iii) in accordance with the terms applicable for US Federal Government officers or employees acting as part of their official duties; on a worldwide, perpetual, irrevocable, royalty-free basis to BMJ Publishing Group Ltd ("BMJ") its licensees and where the relevant Journal is co-owned by BMJ to the co-owners of the Journal, to publish the Work in this journal and any other BMJ products and to exploit all rights, as set out in our licence.

The Submitting Author accepts and understands that any supply made under these terms is made by BMJ to the Submitting Author unless you are acting as an employee on behalf of your employer or a postgraduate student of an affiliated institution which is paying any applicable article publishing charge ("APC") for Open Access articles. Where the Submitting Author wishes to make the Work available on an Open Access basis (and intends to pay the relevant APC), the terms of reuse of such Open Access shall be governed by a Creative Commons licence – details of these licences and which Creative Commons licence will apply to this Work are set out in our licence referred to above.

Other than as permitted in any relevant BMJ Author's Self Archiving Policies, I confirm this Work has not been accepted for publication elsewhere, is not being considered for publication elsewhere and does not duplicate material already published. I confirm all authors consent to publication of this Work and authorise the granting of this licence.

For peer review only

Title

Value-based person-centred integrated care for frail elderly living at home: a quasi-experimental evaluation using Multi-Criteria Decision Analysis

Authors

Maaike Hoedemakers, MSc[1]; Milad Karimi, PhD[1,2]; Fenna Leijten, PhD[1,3]; Lucas Goossens, PhD [1]; Kamrul Islam, PhD [4,5]; Apostolos Tsiachristas, PhD [6]; Maureen Rutten-van Mölken, PhD [1,7]

Affiliations

1: Erasmus School of Health Policy & Management, Erasmus University Rotterdam, the Netherlands

2: OPEN Health, Rotterdam, the Netherlands

3: Staff Defence Healthcare Organisation, Ministry of Defence, Utrecht, the Netherlands

4: Department of Economics, University of Bergen, Bergen, Norway

5: Health Services and Health Economics, NORCE Norwegian Research Centre, Bergen, Norway

6: Health Economics Research Centre, Nuffield Department of Population Health, University of Oxford, Oxford, United Kingdom

7: Institute for Medical Technology Assessment, Erasmus University Rotterdam, Rotterdam, the Netherlands

Contact information corresponding author

Maureen Rutten-van Mölken

Burgemeester Oudlaan 50, 3062 PA Rotterdam

T: +31 10 408 9056

E-mail: m.rutten@eshpm.eur.nl

For peer review only - http://bmjopen.bmj.com/site/about/guidelines.xhtml

Word count: 3680

Abstract

Objective: To evaluate the value of the person-centred, integrated care programme Care Chain Frail Elderly (CCFE) compared with usual care, using Multi-Criteria Decision Analysis (MCDA).

Design: In a 12-month quasi-experimental study, Triple-Aim outcomes were measured at 0, 6 and 12 months by trained interviewers during home-visits.

Setting: Primary care, community-based elderly care.

Participants: 384 community-dwelling frail elderly were enrolled. The 12-month completion rate was 70% in both groups. Propensity score matching was used to balance age, gender, marital status, living situation, education, smoking status, and three-month costs prior to baseline between the two groups.

Intervention: The CCFE is an integrated care programme with unique features like the presence of the elderly and informal caregiver at the multi-disciplinary team meetings, and a bundled payment.

Primary and secondary outcomes measures: The MCDA results in weighted overall value scores that combines the performance on physical functioning, psychological well-being, social relationships and participation, enjoyment of life, resilience, person-centeredness, continuity of care, and costs, with importance weights of patients, informal caregivers, professionals, payers, and policymakers.

Results: At 6 months, the overall value scores of CCFE were higher in all stakeholder groups, driven by enjoyment of life (standardised performance scores 0.729 versus 0.685) and person-centeredness (0.749 versus 0.663). At 12 months, the overall value scores in both groups were similar from a patient's perspective, slightly higher for CCFE from an informal caregiver's and professional's perspective, and

For peer review only - http://bmjopen.bmj.com/site/about/guidelines.xhtml

lower for CCFE from a payer's and policymaker's perspective. The latter was driven by a worse performance on physical functioning (0.682 versus 0.731) and higher costs (€22,816 versus €20,680).

Conclusions: The MCDA indicated that the CCFE is the preferred way of delivering care to frail elderly at 6 months. However, at 12 months, MCDA-results showed little difference from the perspective of patients, informal caregivers, and professionals, while payers and policy makers seemed to prefer usual care.

Key words: Multi-Criteria Decision Analysis, integrated care, frail elderly, triple aim, patient-reported outcome measures

For peer review only

For peer review only - http://bmjopen.bmj.com/site/about/guidelines.xhtml

Strengths and limitations of this study

- We implemented a novel multi-criteria decision analysis (MCDA) framework to evaluate a person-centred integrated care programme for frail elderly.
- We measured a broad range of Triple-Aim outcomes at three time points, among a large and difficult to reach group of the frailest elderly living at home.
- The choice of outcome measures was largely driven by focus groups involving frail elderly.
- The different outcome measures were weighted by their importance, from multiple perspectives, to calculate overall value scores.
- Due to the choice of measuring patient-reported outcomes, participation was too burdensome or impossible for frail elderly with cognitive impairments.

For peer review only

INTRODUCTION

The Care Chain Frail Elderly (CCFE) is an integrated care programme for community-dwelling frail elderly in the Netherlands that offers person-centred care, coordination, and case management to support elderly in living at home for as long as possible.[1] It was developed as part of a movement towards decentralization of long-term care, increased self-sufficiency, and societal participation that was stimulated by the Dutch government to maintain affordability of elderly care. This movement led to a reform of the long-term care sector in 2015.[2] The number of nursing- and residential homes was reduced considerably and access to nursing homes was restricted to those in need of 24-hour care. Municipalities became responsible for the provision of homecare and social support, and health insurers for financing nursing care at home. While the importance of homecare was growing, this sector was confronted with significant budget cuts.[3] The reform accelerated the development of integrated-care initiatives, which were spearheaded by the National Care for the Elderly-Programme commissioned by the Ministry of Health.[4,5]

Integrated care for older people often comprises similar components, namely, comprehensive assessment, individualised care planning, case management, and multidisciplinary team meetings.[6,7] All of these elements are also present in the CCFE. In addition, the intervention is unique in several respects. First, the older person and their informal caregiver are present at the multidisciplinary team meetings where the results of the comprehensive assessment and the individual care plan are discussed. Second, the CCFE is financed by a bundled payment aiming to stimulate collaboration between professionals. The bundled payment is a fixed amount of money per patient that covers all services provided by the GP, nurse-practitioner and physician assistant, regardless of diagnoses, medication review by the pharmacist, telephone consultation by the geriatrician, non-individual-patient-related activities such as building a community network, and overhead. Third, it targets the top-1% frailest elderly registered in a GP-practice who are living at home with complex care needs, using a case-finding approach. The CCFE aims

For peer review only - http://bmjopen.bmj.com/site/about/guidelines.xhtml

to better integrate care across sectors and build a network of support around the patient, and thereby to improve their physical, mental, social health and well-being and experience with care. Ideally, this also reduces secondary care and residential long-term care utilization and thereby costs. The CCFE has previously been described in detail elsewhere.[1]

Although such integrated-care programmes are designed to meet the older person's needs, previous studies show mixed (cost-)effectiveness results.[8–10] A potential explanation is that common methods to assess effectiveness tend to focus on measuring traditional outcomes, such as physical functioning, whereas that is not the primary focus of these programmes.[8,11] Cost-effectiveness analyses measure Quality-Adjusted Life-Year (QALY) gains, which is also less appropriate because integrated care for frail elderly focuses more on well-being than on survival and health-related quality of life.[12] For example, elderly are stimulated to visit outpatient day-care activities to enhance their social participation, or their experience with care is improved by individual care planning and helping them navigate through the health- and social-care system. Accordingly, empirical evaluations should include these domains to accurately value the potential benefits of an intervention.[13,14]

Therefore, we adopted a broader evaluation method, namely Multi-Criteria Decision Analysis (MCDA) to evaluate the CCFE. MCDA is innovative in elderly care because it synthesises a wide variety of outcome measures, in this case patient-reported health and well-being measures (PROMS), experience with care measures (PREMS), and costs. Together, they cover the Triple Aim.[15] In the MCDA outcomes are weighted by their importance according to five stakeholder groups: patients, informal caregivers, professionals, policymakers, and payers. The weighted performance scores are aggregated into overall value scores.[16]

For peer review only - http://bmjopen.bmj.com/site/about/guidelines.xhtml

METHODS

Study population

The study population consisted of community-dwelling frail elderly, where frailty is defined as being in need of complex care due to loss of functional abilities and control over one's life. To participate in the intervention, they had to be registered at a GP-practice from one of three care groups (i.e., an association of primary care providers that cooperate in the provision of chronic care) that offer the CCFE. They had to be able to comprehend study information and answer questions, either independently or with the help of an informal caregiver or trained interviewer.

Intervention

A primary care team consisting of the GP, nurse-practitioner, and district nurse, identifies potential candidates for the CCFE using a case-finding approach. To be included in the CCFE, the health insurer does not require a specific diagnosis or the use of a screening tool. They trust that the GP and nurse practitioner know their patients and are most suited to select the elderly that may benefit from the care programme. Furthermore, there is an agreement with the health insurer that only the top 1% frail elderly of a GP-practice will be included in the programme. Subsequently, the nurse-practitioner visits the older person at home and performs a comprehensive geriatric assessment of the needs, capabilities, and preferences in the physical, psychological, cognitive, and social domains. Depending on this assessment, the nurse-practitioner consults professionals in the community (e.g., physical therapists, occupational therapists, social workers, elderly care physicians, geriatricians, dementia case workers) and arranges informal care support. Together with the frail elderly and informal caregiver, the nurse-practitioner drafts an individual care plan, largely driven by the elderly's personal goals.

The nurse-practitioner organises multidisciplinary team meetings to discuss the individual care plan with all professionals involved. The frail elderly and their informal caregiver participate in these meetings.

For peer review only - http://bmjopen.bmj.com/site/about/guidelines.xhtml

For peer review only

During the first meeting a case manager is assigned, a role mostly taken up by the nurse-practitioner. The case manager is the main contact point, monitors the execution of the individual care plan, and further adapts the care to the patient's wishes and additional needs. Once enrolled, an elderly person usually stays in the CCFE until nursing home admission or if they pass away.

To support collaboration between professionals, the CCFE uses 'Care2U', a secured ICT-platform to share information. Professionals have different degrees of access and the elderly must approve access. Care2U includes the individual care plan and is used by the nurse-practitioner to monitor appointments and services of providers in the care chain.

The CCFE is financed by a bundled payment contract between each care group and the dominant health insurer in the region and is re-negotiated every year. These care groups are legal entities of primary care providers who develop chronic care programs, support the provision of these programmes and are contracted by the health insurer to coordinate chronic care in a region.

Study design

The MCDA was conducted as part of a 12-month prospective quasi-experimental study comparing two parallel groups:

Intervention group included frail elderly enrolled into the CCFE between April 2017-August 2018 and were recruited to participate in the study by either their GP or nurse-practitioner.

Control group consisted of frail elderly receiving usual care, recruited at GP-practices from one of three participating care groups. These practices had not (yet) implemented the CCFE. To ensure a similar level of frailty in both groups, the GP-practices in the control group applied the same case-finding approach. They were assisted by a GP specialised in elderly care that had experience with the CCFE.

For peer review only - http://bmjopen.bmj.com/site/about/guidelines.xhtml

Self-reported outcome and experience measures and care utilization data were gathered at baseline and after 6 months and 12 months, during face-to-face interviews at the elderly's home, performed by trained interviewers. The interviewers were not involved in delivering any aspects of the intervention but were aware of which group the respondent belonged to. Data collection took place between April 2017-August 2019.

Outcome measures

Table 1 gives an overview of all outcome measures. These outcomes were selected based on a literature review, workshops with representatives from the five stakeholder groups, and focus groups with individuals with multi-morbidity, and measured with validated questionnaires.[17]

For peer review only

Table 1. Outcome measures and instruments

Core set^	Instrument to measure outcome	Scale
**Health & Well-being**		
Physical functioning	Activities of Daily Living (ADL) (Katz-15)[18]	0-15 (worst)
Psychological well-being	Mental Health Inventory (MHI-5)[19]	0-100 (best)
Enjoyment of life	Investigating Choice Experiments for the Preferences of Older People (ICECAP-O)[20]	1-4 (best)
Social relationships & participation	Impact on Participation & Autonomy (IPA), social life and relationships domain[21]	0-28 (worst)
Resilience	Brief Resilience Scale (BRS)[22]	6-30 (best)
**Experience of care**		
Person-centeredness	Person-centered Coordinated Care Experience Questionnaire (P3CEQ), experience of person-centered care domain[23]	0-18 (best)
Continuity of care	Nijmegen Continuity Questionnaire (NCQ), team and cross boundary continuity domain[24] + Client Perceptions of Coordination Questionnaire (CPCQ)[25]	1-5 (best)
**Costs**		
Health, social, and informal care costs	iMTA Medical Consumption Questionnaire[Error! Reference source not found.]	

For peer review only - http://bmjopen.bmj.com/site/about/guidelines.xhtml

Medication costs	Prescriptions in patient records extracted from GP information systems	
Bundled payments and chronic care programmes	Care chain information system 'Care2U'	
**Additional outcomes**		
Autonomy	Pearlin Mastery Scale[26]	7-35 (best)
Burden of medication	Living with Medicines Questionnaire - LMQ[28]	0-10 (worst)

^The core set of outcomes was measured across all studies included in the SELFIE project. For these outcomes, weights were elicited, and these outcomes were included in the MCDA. The additional outcomes were not included in the MCDA.

Costs

Health- and social-care utilization data were obtained with the iMTA-Medical Consumption Questionnaire, which includes questions about contacts with GP, nurse-practitioner, GP-assistants, physiotherapists and other paramedical therapists, dieticians, psychologists, social workers, welfare workers, and medical specialists, hospital admissions, rehabilitation, homecare, residential care and nursing homes, and informal care during the past three months.[Error! Reference source not found.] Unit costs were largely based on reference prices from the Dutch Costing Manual.[29] Medication costs were based on prescription data from GP-information systems, which were combined with unit costs from Dutch drug database '*G-Standaard*', using ATC-codes.[30] Programme costs of the CCFE were based on the bundled payment contracts between each care group and the dominant health insurer.[1] Elderly from the control group could participate in single-disease care programmes for diabetes, cardiovascular risk management or Chronic Obstructive Pulmonary Disease. The proportion of elderly participating in these programmes was obtained from Care2U and average prices of the bundled payments were obtained from the Dutch health claims database (Vektis).[31]

Statistical analysis

Comparability of groups

For peer review only

For peer review only - http://bmjopen.bmj.com/site/about/guidelines.xhtml

181 We applied inverse probability weighting to increase the comparability of the intervention and control groups at baseline.[32] The logistic regression model to estimate the propensity score included age, gender, marital status, living situation, educational level, smoking status, and costs three months prior to baseline as a proxy for complexity. The propensity score *p* reflects the estimated probability of an individual to be in the intervention group. By setting the weight for individuals in the intervention group to 1, and for individuals in the control group to *p/(1-p)*, we estimated the average treatment effect in the treated (ATT) in the weighted mixed effect models described below.[33] To assess the comparability of the two groups, we calculated the mean percentage standardised bias, the Rubin's B (absolute standardised difference of the means of the linear index of the propensity score in the intervention group and matched controls), and the Rubin's R (ratio of intervention group and matched control group variances of the propensity score index). For sufficient balance, B should be less than 25% and R be between 0.5 and 2.[34]

<u>Treatment effects</u>

Treatment effects were estimated using weighted mixed effect models with a random intercept at individual level (as we had longitudinal data) and the following covariates: time, intervention, an interaction term for time and intervention, age, gender, marital status, living situation, educational level, and smoking status. This combination of matching and regression adjustment has been shown to best reduce covariate imbalance between groups.[35] The mixed effects models were used to predict the mean scores of the outcome measures in both groups at each time point, assuming the control group had the same baseline score as the intervention group to directly compare both groups. All statistical analyses were performed using STATA16.1.

<u>MCDA</u>

In the MCDA, the predicted mean scores of the outcomes at 6- and 12-months follow-up were standardised on a 0-1 scale to remove differences in measurement scales, using relative standardization,

For peer review only - http://bmjopen.bmj.com/site/about/guidelines.xhtml

205 see **Appendix 1**. For all outcomes in the MCDA, a higher score indicates better performance. The standardised outcomes were weighted by their importance and subsequently summed to obtain an overall value score for the intervention and control group separately. The relative importance-weights were elicited in an online weight elicitation study among patients, informal caregivers, professionals, payers, and policymakers, using a discrete choice experiment (DCE).[36] The relative weights of the outcome measures included in the MCDA by stakeholder group can be found in **Appendix 2**.

211 Sensitivity analysis

212 To assess the joint uncertainty in outcome scores and importance-weights, we performed a probabilistic sensitivity analysis using Monte Carlo simulation. We used Cholesky decomposition to obtain 10,000 replications of both the standardised outcomes and the importance-weights. From this, we obtained the 95%-confidence intervals around the overall value scores for each stakeholder group. Additionally, we calculated the proportion of MCDA-iterations in which the CCFE has a higher overall value score than usual care.

218 *Patient and Public Involvement*

219 Patients were involved in the selection of outcome measures, which was largely based on focus groups as described above.[17] In multiple National Stakeholder Workshops held during the entire process of the study, stakeholders from the five stakeholder groups were asked to reflect upon the outcome measures, the study design and data collection, and the results of the study. The study design was set-up in close collaboration with care providers to ensure feasibility of the data collection. Before the start of data collection, the questionnaire was piloted in a frail older person. The authors disseminated results via conference presentations. Results of this study were also disseminated to participating care providers, patients and informal caregivers using communication methods other than scientific papers, i.e., by e-mail and newsletters disseminated at the GP-practices.

For peer review only - http://bmjopen.bmj.com/site/about/guidelines.xhtml

Ethics approval and consent to participate

The study was conducted as one of the case studies in Horizon2020-project SELFIE (Sustainable intEgrated care modeLs for multi-morbidity: delivery, FInancing and performancE).[37] The study protocol was reviewed by the medical ethics committee of the Erasmus Medical Centre, the Netherlands. The committee concluded that the rules laid down in the Dutch Medical Research Involving Human Subjects Act do not apply to this research (*MEC-2017-121*). All study participants have provided informed written consent.

For peer review only

For peer review only - http://bmjopen.bmj.com/site/about/guidelines.xhtml

RESULTS

Respondents

Figure 1 presents the flow chart of patients included in the study. The retention rate at 12-month follow-up was 70% in both groups. Main reasons of loss-to-follow-up were also similar in both groups and included death, burden of study participation, and cognitive incapacity.

*****Figure 1 here*****

Table 2 presents the baseline characteristics of respondents before and after inverse probability weighting. After IPW, the matching statistics were within the desired range (Rubin's B < 25%, Rubin's R 0.5 – 2). No substantial differences between the groups remained. The common support graph shows a good overlap in propensity scores, see **Appendix 3**.

For peer review only

For peer review only - http://bmjopen.bmj.com/site/about/guidelines.xhtml

Table 2. Baseline characteristics before and after inversed probability weighting

	CCFE N=222	UC N=162	St. diff.	UC N=162	St. diff.
**Baseline characteristics**	**Before IPW**			**After IPW**	
Age, mean	83.4	84.8	-0.224	83.6	-0.028
Female (%)	63.5	64.2	-0.014	64.8	-0.027
Marital status (%)					
Single, never married	3.6	3.7	-0.005	2.6	0.054
Married or living together	44.6	40.7	0.078	45.0	-0.008
Widow(er)	44.6	50.6	-0.120	47.1	-0.049
Divorced	7.2	4.9	0.095	5.4	0.078
Living situation (%)					
Independent, alone	54.1	61.1	-0.143	55.2	-0.024
With others	46.0	38.9	0.143	44.8	0.024
Educational level (%)					
Low	70.3	70.4	-0.002	73.3	-0.067
Medium	20.3	14.8	0.143	14.3	0.158
High	9.5	14.8	-0.164	12.4	-0.090
Current smoker (%)	14.4	8.6	0.181	13.8	0.019
**Outcome measures at baseline, mean**	**Before IPW**			**After IPW**	
Physical functioning (0-15) ^	4.4	4.5	-0.032	4.5	-0.052
Psychological well-being (0-100)	71.4	71.7	-0.018	70.6	0.040
Enjoyment of life (1-4)	2.8	2.9	-0.137	2.9	-0.143
Social relationships and participation (0-28) ^	9.2	8.0	0.345	8.2	0.280
Resilience (6-30)	19.3	19.0	0.062	19.0	0.082
Autonomy (7-35)	22.3	22.1	0.054	21.9	0.098
Burden of medication (0-10) ^	2.1	2.4	-0.111	2.5	-0.166
Person-centeredness (0-18)	11.7	12.5	-0.209	12.7	-0.246
Continuity of care (1-5)	3.7	3.8	-0.140	3.8	-0.182
Total costs 3 months prior to the study (€) ^	5,453	5,267	0.028	5,631	-0.026
**Statistics to assess matching[#]**	**Before IPW**			**After IPW**	
Mean bias			11.9		5.1
Rubin's B			39.7		21.4
Rubin's R			1.06		1.10

^ Higher score indicating worse performance; St. diff = Absolute Standardised Mean Difference, also called Absolute Standardised Bias; # On variables used in PSM, i.e., age, gender, marital status, living situation, educational level, smoking, total costs 3 months prior to the study. CCFE=Care Chain Frail Elderly. UC=Usual care. IPW=Inverse Probability Weighting

For peer review only

Treatment effects

Table 3 reports the estimated treatment effects of the CCFE at 6- and 12-months of follow-up. Results show that the CCFE improved person-centeredness at both time points. At 6 months follow-up, physical functioning declined in both groups but even further in the intervention group. At 12 months follow-up, the CCFE performed worse on autonomy and burden of medication. The predicted mean performance scores of all outcomes on their natural scale can be found in **Appendix 4**.

For peer review only

For peer review only - http://bmjopen.bmj.com/site/about/guidelines.xhtml

Table 3. Treatment effects at 6- and 12-months follow-up

	**6 months**			**12 months**		
**Core set of outcomes included in the MCDA**	**Estimated change CCFE**	**Estimated Change UC**	**Diff in change Mean (95% CI)[#]**	**Estimated change CCFE**	**Estimated change UC**	**Diff in change Mean (95% CI)[#]**
**Health & Well-being**						
Physical functioning (0-15) ^	0.74	0.27	0.47 (0.06 ; 0.88)*	1.33	0.95	0.39 (-0.10 ; 0.87)
Psychological well-being (0-100)	-0.64	-0.34	-0.30 (-3.86 ; 3.25)	-1.35	-0.10	-1.26 (-5.20 ; 2.68)
Enjoyment of life (1-4)	0.10	-0.08	0.18 (-0.02 ; 0.38)	0.05	0.02	0.04 (-0.18 ; 0.25)
Social relationships & participation (0-28) ^	0.11	0.41	-0.31 (-1.04 ; 0.43)	0.26	0.61	-0.36 (-1.25 ; 0.54)
Resilience (6-30)	-0.02	-0.21	0.19 (-0.63 ; 1.01)	0.03	0.11	-0.08 (-1.01 ; 0.85)
**Experience of care**						
Person-centeredness (0-18)	1.10	-0.38	1.48 (0.57 ; 2.38)*	1.33	0.00	1.33 (0.18 ; 2.49)*
Continuity of care (1-5)	0.10	-0.02	0.12 (-0.05 ; 0.28)	0.16	0.03	0.13 (-0.05 ; 0.31)
**Costs**						
Total health and social care costs^	5,405	4,745	660 (-1,650 ; 2,970)	17,223	15,206	2,017 (-2,361 ; 6,395)
**Additional frail elderly-specific outcomes**	**Estimated change CCFE**	**Estimated change UC**	**Diff in change Mean (95% CI)[#]**	**Estimated change CCFE**	**Estimated change UC**	**Diff in change Mean (95% CI)[#]**
Autonomy (7-35)	-0.50	0.02	-0.52 (-1.38 ; 0.34)	-0.49	0.55	-1.04 (-1.95 ; -0.14)*
Burden of medication (0-10) ^	0.13	-0.21	0.34 (-0.36 ; 1.04)	0.18	-0.61	0.78 (0.04 ; 1.53)*

*^ Higher score indicating worse performance; [#] based on robust SE; * p<0.05. MCDA=Multi-Criteria Decision Analysis. CCFE=Care Chain Frail Elderly. UC=Usual care. CI=Confidence interval.*

For peer review only - http://bmjopen.bmj.com/site/about/guidelines.xhtml

Costs

Table 4 presents details on the mean costs after inversed probability weighting at 6- and 12-months, from a healthcare and a societal perspective. From a healthcare perspective, after 6 months, costs were €751 higher in the intervention group than in the control group. After 12 months, the costs were €1,796 higher. These differences were largely due to programme costs and costs of homecare.

When adopting a societal perspective, 6-month costs were €662 higher in the intervention group than in the control group, whereas 12-month costs were €2,136 higher. Costs of informal care were slightly lower in the intervention group than in the control group after 6 months, but higher after 12 months.

For peer review only - http://bmjopen.bmj.com/site/about/guidelines.xhtml

Table 4. Mean costs (€) after 6- and 12-months of follow-up (after IPW)

	**6 months**			**12 months**		
**Cost category**	**Mean costs (SD) CCFE (n=172)**	**Mean costs (SD) UC (n=129)**	**Difference between means (SE)**	**Mean costs (SD) CCFE (n=156)**	**Mean costs (SD) UC (n=113)**	**Difference between means (SE)**
Chronic care programme(s) #	534 (-)	72 (-)	462 (-)	1,068 (-)	143 (-)	925 (-)
Homecare	3,289 (4,371)	3,158 (4,016)	131 (481)	7,597 (8474)	6,330 (7680)	1,267 (980)
Long-term care admissions	969 (5,411)	766 (4,980)	203 (589)	1,624 (7,959)	2,051 (10,025)	-427 (11,33)
Hospital admissions	670 (2,670)	485 (1,833)	185 (259)	1,199 (3,603)	1,119 (2,992)	80 (403)
Emergency room visits	112 (308)	116 (341)	-4 (38)	179 (372)	196 (418)	-17 (49)
Outpatient day-care activities	235 (1,180)	310 (1,481)	-74 (170)	627 (2,642)	408 (1,846)	218 (285)
Medical specialist care	338 (619)	317 (408)	21 (60)	631 (789)	609 (615)	23 (86)
Paramedical care (e.g., physiotherapist)	529 (636)	481 (548)	48 (72)	1,059 (1,113)	1,005 (1,133)	55 (145)
General practitioner ~	21 (27)	288 (272)	-267 (28)	25 (34)	492 (394)	-467 (44)
Medication	363 (662)	318 (551)	45 (65)	744 (1,232)	605 (927)	139 (124)
**Subtotal costs from healthcare perspective**	**7 060 (8,441)**	**6,310 (7,671)**	**751 (922)**	**14,753 (14,498)**	**12,957 (14,185)**	**1,796 (1,777)**
Informal care	3,690 (5,780)	3,779 (7,318)	-89 (891)	8,063 (12,436)	7,723 (13,643)	340 (1,783)
**Total costs from societal perspective**	**10,750 (10,381)**	**10,089 (11,003)**	**662 (1,309)**	**22,816 (19,050)**	**20,680 (20,251)**	**2,136 (2,551)**

# Costs for chronic care program(s) is an average estimation which is the same for each respondent in their respective group; ~ Costs for GP-care in the intervention group are largely included in the costs of the chronic care program. SD=Standard deviation. SE=Standard Error. CCFE=Care Chain Frail Elderly. UC=Usual care.

For peer review only - http://bmjopen.bmj.com/site/about/guidelines.xhtml

Multi-Criteria Decision Analysis

Table 5a and b present the standardised outcome scores for the CCFE and usual care as well as these scores weighted according to each stakeholder's importance-weights, at 6- and 12-months, respectively. The overall value scores show that all stakeholder groups preferred the CCFE over usual care at 6 months follow-up. This was driven by the performance scores of person-centeredness and enjoyment of life, and the high importance-weight of the latter. In more than 75% of iterations the CCFE had a higher overall value score than usual care. At 12 months follow-up the difference disappeared, and the probability that the CCFE had a higher overall value score dropped below 50% for payers and policymakers. This was driven by worse scores in physical functioning and costs.

For peer review only

For peer review only - http://bmjopen.bmj.com/site/about/guidelines.xhtml

Table 5a. Value scores in the Multi-Criteria Decision Analysis at 6 months, using DCE weights

			Patients		Partners		Professionals		Payers		Policymakers	
**Outcome measures**	**Standardised performance score#**		**Weighted score**		**Weighted score**		**Weighted score**		**Weighted score**		**Weighted score**	
	**CCFE**	**UC**	**CCFE**	**UC**	**CCFE**	**UC**	**CCFE**	**UC**	**CCFE**	**UC**	**CCFE**	**UC**
Physical functioning	0.672	0.740	0.108	0.118	0.074	0.081	0.081	0.089	0.094	0.104	0.094	0.104
Psychological well-being	0.706	0.709	0.120	0.120	0.106	0.106	0.127	0.128	0.127	0.128	0.106	0.106
Enjoyment of life	0.729	0.685	0.168	0.157	0.182	0.171	0.160	0.151	0.175	0.164	0.160	0.151
Social relationships and participation	0.718	0.696	0.057	0.056	0.065	0.063	0.079	0.077	0.072	0.070	0.072	0.070
Resilience	0.711	0.704	0.107	0.106	0.099	0.099	0.092	0.091	0.078	0.077	0.099	0.099
Person-centeredness	0.749	0.663	0.060	0.053	0.060	0.053	0.060	0.053	0.045	0.040	0.060	0.053
Continuity of care	0.718	0.696	0.072	0.070	0.086	0.083	0.079	0.077	0.057	0.056	0.072	0.070
Costs (societal care perspective)	0.685	0.729	0.021	0.022	0.041	0.044	0.041	0.044	0.055	0.058	0.048	0.051
**Overall value scores**			**0.711**	**0.702**	**0.713**	**0.700**	**0.719**	**0.708**	**0.703**	**0.696**	**0.711**	**0.702**
**95% Confidence interval^**			0.702 - 0.721	0.692 - 0.712	0.703 - 0.723	0.690 - 0.710	0.702 - 0.722	0.691 - 0.711	0.699 - 0.721	0.692 - 0.714	0.700 - 0.721	0.692 - 0.712
**% CCFE > UC^**			82%		90%		87%		75%		81%	

For peer review only - http://bmjopen.bmj.com/site/about/guidelines.xhtml

Table 5b. Value scores in the Multi-Criteria Decision Analysis at 12 months, using DCE weights

			Patients		Partners		Professionals		Payers		Policymakers	
Outcome measures	Standardised performance score#		Weighted score		Weighted score		Weighted score		Weighted score		Weighted score	
	CCFE	UC	CCFE	UC	CCFE	UC	CCFE	UC	CCFE	UC	CCFE	UC
Physical functioning	0.682	0.731	0.109	0.117	0.075	0.080	0.082	0.088	0.095	0.102	0.095	0.102
Psychological well-being	0.701	0.713	0.119	0.121	0.105	0.107	0.126	0.128	0.126	0.128	0.105	0.107
Enjoyment of life	0.711	0.703	0.164	0.162	0.178	0.176	0.157	0.155	0.171	0.169	0.157	0.155
Social relationships and participation	0.720	0.694	0.058	0.056	0.065	0.062	0.079	0.076	0.072	0.069	0.072	0.069
Resilience	0.706	0.709	0.106	0.106	0.099	0.099	0.092	0.092	0.078	0.078	0.099	0.099
Person-centeredness	0.744	0.668	0.060	0.053	0.060	0.053	0.060	0.053	0.045	0.040	0.060	0.053
Continuity of care	0.719	0.695	0.072	0.069	0.086	0.083	0.079	0.076	0.058	0.056	0.072	0.069
Costs (societal care perspective)	0.673	0.739	0.020	0.022	0.040	0.044	0.040	0.044	0.054	0.059	0.047	0.052
**Overall value scores**			**0.707**	**0.707**	**0.708**	**0.706**	**0.714**	**0.713**	**0.698**	**0.702**	**0.706**	**0.707**
**95% Confidence interval^**			0.696 - 0.717	0.697 - 0.717	0.697 - 0.718	0.695 - 0.716	0.697 - 0.717	0.695 - 0.716	0.695 - 0.717	0.697 - 0.719	0.695 - 0.717	0.696 - 0.718
**% CCFE > UC^**			50%		57%		54%		39%		48%	

^ based on Monte Carlo simulation; # colour scheme ranges from red (lowest score) to green (highest score). CCFE=Care Chain Frail Elderly. UC=Usual care.

For peer review only - http://bmjopen.bmj.com/site/about/guidelines.xhtml

DISCUSSION

Main findings

The MCDA has shown that all stakeholders preferred the CCFE over usual care at 6 months with a likelihood of having a higher overall value score of over 75%. This was mainly driven by higher performance on enjoyment of life and person-centeredness, and the great importance of the former outcome. Results became more diffuse at 12 months. Patients were indifferent, informal caregivers, and professionals slightly favoured the CCFE, whereas payers and policymakers demonstrated a slight preference for usual care. This was mainly due to a worse performance of the CCFE on physical functioning and costs. When looking at the disaggregated scores, person-centeredness was consistently higher in the CCFE. Physical functioning deteriorated in the intervention group at 6 months, but this effect disappeared at 12 months. When some outcomes improve whereas others deteriorate, the current MCDA approach is a suitable method to aggregate them into overall value scores that vary depending on the importance that stakeholders assign to the different outcome measures. These results show that the CCFE is the preferred way of delivering care to frail elderly if improvements in enjoyment of life and person-centeredness are considered more important than physical functioning and costs.

Context and comparison with previous evaluations

As this is the first MCDA of a frail elderly program, it is impossible to directly compare the value scores to other studies. MCDA also provides insight in the disaggregated effects of the CCFE, but these are hard to compare to other studies as well, due to the very frail target group of the CCFE, the different contexts in which the interventions are implemented, the different intervention components, and outcome measures[38,39] Regarding the context of the CCFE, we should stress that the programme was implemented in a setting with a strong primary care sector. GPs already have a

For peer review only - http://bmjopen.bmj.com/site/about/guidelines.xhtml

history of collaborating with other primary care providers, for example by working jointly in health centres. Hence, setting up community networks and collaborating in wider multi-disciplinary teams was not such a big step. When implementing similar interventions in countries with a less strong primary care system, collaboration may require more efforts. On the other hand, the potential for savings due to the intervention might be higher in countries where the GPs does not act as a gatekeeper to secondary care, as a programme like the CCFE could substitute more secondary care services by primary care services.

Despite differences between studies, most previous studies did not find effects on physical functioning.[8,40] This may be expected as these programmes rarely aim to achieve improvements in this domain. As the CCFE aimed to improve experience with care, the sustained improvement in person-centeredness found in this study suggests that the programme has fulfilled that aim.[1] The worsening in autonomy and burden of medication seems counterintuitive. A possible explanation for the deterioration in autonomy is that as elderly in the CCFE were confronted with their frailty, e.g., by discussing their needs for support, they became more aware of their loss of control. This could have led to lower autonomy scores in the intervention group, especially due to the self-report method; measurement by a third party might have led to different scores.[41] The higher medication burden in the intervention group could be explained by alterations in medication after the medication review, which may have led to (temporary) side-effects.

One could question the relevance of finding a sustained effect on an outcome (i.e., person-centredness) that was less highly valued than other outcomes in the DCE. This may raise the question whether the aim of the CCFE was well-targeted. However, weights were derived from a DCE that asks respondents to choose between two hypothetical care programmes, which gives them the opportunity to trade-off person-centredness for, for example, improved physical functioning. Even though this is likely to be an appropriate reflection of their preferences if all options were open, in real life this trade-off may no longer exist, because improving physical functioning might not

For peer review only - http://bmjopen.bmj.com/site/about/guidelines.xhtml

be possible anymore. Hence, there is a discrepancy between what is important to a patient and what is feasible in practice.

Strengths & limitations

One of the strengths of our study was its controlled study design. Defining an appropriate control group to evaluate ongoing programmes for frail elderly is a challenge.[8,42] In the current study, a potential limitation concerns the case finding approach to identify the target population of both groups, namely frail elderly in need of complex care due to loss of functional abilities and control over one's life. In the intervention group this was done by GPs offering the programme and in the control group by GPs not offering the programme. To ensure a similar level of frailty in both groups, the latter GPs were assisted by a GP specialised in elderly care. This has been successful as the baseline characteristics of both groups were quite similar. It is especially important in evaluating frail elderly care, as there is commonly little room for actual improvements in health, and prevention or delay of deterioration can only be shown in comparison with a control group.

Another strength was the data collection on a broad range of PROMs and PREMs by interviewers who made a total of 954 home-visits, which was a major endeavour. Collecting patient-reported data did limit the generalisability of the results, as some frail elderly could not participate in the evaluation because that was too burdensome or impossible. Hence, the frailest among the elderly were not represented in this study, especially not those with dementia. The attrition rate in our study was relatively low, i.e., 30% across both groups at 12 months. We did observe that respondents in the control group that were lost to follow-up were slightly older and had worse physical functioning at baseline compared to non-dropouts in the control group (and overall), see **Appendix 5**. This may have led to an underestimation of the treatment effect.

A further strength of our study was the detailed cost-analysis, even including costs of medication, social care and informal care, which are often excluded from other studies.[8] This analysis showed a

For peer review only - http://bmjopen.bmj.com/site/about/guidelines.xhtml

cost increase reflecting the greater investment of resources to support frail elderly in ageing in place which is of great importance to many older persons.

In the design and reporting on the MCDA we followed the good practice guidelines as laid out by the ISPOR MCDA-taskforce.[43] Strengths of MCDA are that it enables explicit, transparent and accountable decision-making, i.e., for every decision what was valued most and by whom can be tracked down as well as whether this was due to improvements in certain domains or a higher relative importance of a particular outcome. Furthermore, MCDA makes it possible to include additional elements of value that go beyond health or QALYs, which is especially important for complex interventions with multiple aims such as improving well-being and experience with care. However, the consequence is that we may favour interventions that achieve improvements in these outcomes above interventions that have greater health outcomes. This may be justifiable for elderly care. Such an argument would raise another point that is debated in MCDA, namely whether or not to include costs in the overall value score.[44,45] To elicit a weight for costs, stakeholders had to trade-off costs against other outcomes, which makes the relative contribution of costs to the overall value score explicit. However, it can be argued that this does not adequately address the opportunity costs of the CCFE.[46] We also performed the MCDA without costs as a sensitivity analysis, see **Appendix 6**, which led to higher overall value scores for the CCFE at both time points. However, now the overall value forms a composite benefit score for which a new cost-effectiveness threshold must be determined. Although we believe the current set of outcomes captures the full potential value of an integrated care programme for frail elderly, this set should be tailored to each intervention's aims and target group. Therefore, when the set of outcome measures changes, new thresholds need to be determined. Another option is to calculate the cost-per-value and prioritise interventions with the lowest cost-per-value ratio, but this only leads to a ranking of interventions.[45] A last point of discussion on MCDA is that it requires a deliberative component to avoid making decisions based solely on the model.[45] In our study we presented results from five stakeholder perspectives which inevitably calls for further deliberation to determine which perspective should prevail.

For peer review only - http://bmjopen.bmj.com/site/about/guidelines.xhtml

Implications

Although the CCFE does not improve the (physical) health of patients, it is still positively evaluated by all stakeholder groups at 6 months. At 12 months stakeholders were mainly indifferent. This warrants further research into interventions to maintain the effects of such programmes in the long-term. Furthermore, we advocate a wider use of MCDA to evaluate multi-faceted, person-centred, integrated care programmes for frail elderly that aim to improve multiple outcomes, including those that go beyond health. MCDA enables a transparent and explicit decision-making process and serves as a tool to help decision-makers reach a decision. Therefore, MCDA-results are a good starting point for deliberation before deciding upon reimbursement or broader implementation of new interventions.

CONCLUSION

After 6 months, the overall value score for the CCFE was higher than for usual care across all stakeholders, but at 12 months, the preference for the CCFE had disappeared. The CCFE led to sustained improvements in enjoyment of life and person-centeredness, which is aligned with the program's aim, but also to a deterioration in physical functioning at 6 months and higher costs. Therefore, the CCFE is only the preferred way of delivering care to frail elderly in case improvements in enjoyment of life and person-centeredness are considered more important than costs and physical functioning.

For peer review only

For peer review only - http://bmjopen.bmj.com/site/about/guidelines.xhtml

402 **ACKNOWLEDGEMENTS**

403 We would like to thank all respondents and their informal caregivers for participating in this
404 research. We would also like to thank the care groups DOH, SGE and PoZoB for their continuous
405 support in facilitating this research.

406 **AUTHOR CONTRIBUTIONS**

407 MH, MK, FL, AT, MRvM developed the overall methodology for the value-based MCDA approach in
408 the SELFIE project. MH, MK, FL, MRvM contributed to the study design and data collection. MH, MK,
409 LG, KI, AT, MRvM had access to the data and contributed to the data analysis. MH and MRvM
410 drafted this manuscript. All co-authors critically reviewed this manuscript. All authors read and
411 approved the final manuscript. MH and MRvM had full access to all of the data in the study and the
412 final responsibility to submit for publication.

413 **DECLARATION OF INTERESTS**

414 On submission, MK was affiliated to OPEN Health. No other disclosures were reported.

415 **FUNDING**

416 The SELFIE project has received funding from the European Union's Horizon 2020 research and
417 innovation programme under grant agreement No 634288. The content of this manuscript reflects
418 only the SELFIE group's views and the European Commission is not liable for any use that may be
419 made of the information contained herein.

420 **DATA SHARING AGREEMENT**

421 The data that support the findings of this study are available on request from the corresponding
422 author, MH. The data are not publicly available due to privacy restrictions.

For peer review only - http://bmjopen.bmj.com/site/about/guidelines.xhtml

REFERENCES

1. Hoedemakers M, Leijten FRM, Looman W, Czypionka T, Kraus M, Donkers H, et al. Integrated Care for Frail Elderly: A Qualitative Study of a Promising Approach in The Netherlands. International Journal of Integrated Care. 2019 Sep 3;19(3):16.
2. Maarse JAM, Jeurissen PP. The policy and politics of the 2015 long-term care reform in the Netherlands. Health Policy. 2016 Mar 1;120(3):241–5.
3. Alders P, Schut FT. The 2015 long-term care reform in the Netherlands: Getting the financial incentives right? Health Policy. 2019 Mar 1;123(3):312–6.
4. Wehrens R, Oldenhof L, Verweij L, Francke A, Bal R. Experimenteel sturen in netwerken: een evaluatie van proces en structuur van het Nationaal Programma Ouderenzorg. :99.
5. de Jong B, Wynia K, Geluk-Bleumink A. Ageing Better in the Netherlands. In D'Onofrio G, Sancarlo D, Greco A, editors, Gerontology. London: IntechOpen. 2018. p. 101-111
6. Briggs AM, Valentijn PP, Thiyagarajan JA, Carvalho IA de. Elements of integrated care approaches for older people: a review of reviews. BMJ Open. 2018 Apr 1;8(4):e021194.
7. Hendry A, Vanhecke E, Carriazo A, López-Samaniego L, Espinosa J, Sezgin D, et al. Integrated Care Models for Managing and Preventing Frailty: A Systematic Review for the European Joint Action on Frailty Prevention (ADVANTAGE JA). Transl Med UniSa. 2019 Jan 6;19:5–10.
8. Looman WM, Huijsman R, Fabbricotti IN. The (cost-)effectiveness of preventive, integrated care for community-dwelling frail older people: A systematic review. Health & Social Care in the Community. 2019;27(1):1–30.
9. Van der Elst M, Schoenmakers B, Duppen D, Lambotte D, Fret B, Vaes B, et al. Interventions for frail community-dwelling older adults have no significant effect on adverse outcomes: a systematic review and meta-analysis. BMC Geriatrics. 2018 Oct 20;18(1):249.

For peer review only - http://bmjopen.bmj.com/site/about/guidelines.xhtml

10. Hopman P, de Bruin SR, Forjaz MJ, Rodriguez-Blazquez C, Tonnara G, Lemmens LC, et al. Effectiveness of comprehensive care programmes for patients with multiple chronic conditions or frailty: A systematic literature review. Health Policy. 2016 Jul 1;120(7):818–32.

11. Vorst A van der, Zijlstra GAR, Witte ND, Duppen D, Stuck AE, Kempen GIJM, et al. Limitations in Activities of Daily Living in Community-Dwelling People Aged 75 and Over: A Systematic Literature Review of Risk and Protective Factors. PLOS ONE. 2016 Oct 19;11(10):e0165127.

12. Makai P. Moving beyond the QALY: economic evaluation in health and social care for elderly populations. [Rotterdam]: Erasmus Universiteit Rotterdam; 2014.

13. Cramm J, Nieboer A. Is ”disease management” the answer to our problems? No! Population health management and (disease) prevention require ”management of overall well-being” BMC Health Services Research. 2016 Sep 21;16.

14. Makai P, Brouwer WBF, Koopmanschap MA, Stolk EA, Nieboer AP. Quality of life instruments for economic evaluations in health and social care for older people: A systematic review. Social Science & Medicine. 2014 Feb 1;102:83–93.

15. Berwick DM, Nolan TW, Whittington J. The Triple Aim: Care, Health, And Cost. Health Affairs. 2008 May 1;27(3):759–69.

16. Rutten-van Mölken M, Leijten F, Hoedemakers M, Tsiachristas A, Verbeek N, Karimi M, et al. Strengthening the evidence-base of integrated care for people with multi-morbidity in Europe using Multi-Criteria Decision Analysis (MCDA). BMC Health Services Research. 2018 Jul 24;18(1):576.

17. Leijten FRM, Hoedemakers M, Struckmann V, Kraus M, Cheraghi-Sohi S, Zemplényi A, et al. Defining good health and care from the perspective of persons with multimorbidity: results from a qualitative study of focus groups in eight European countries. BMJ Open. 2018 Aug 1;8(8):e021072.

For peer review only

18. Laan W, Zuithoff NPA, Drubbel I, Bleijenberg N, Numans ME, De Wit NJ, et al. Validity and reliability of the Katz-15 scale to measure unfavorable health outcomes in community-dwelling older people. J Nutr Health Aging. 2014 Nov 1;18(9):848–54.

19. Rumpf H-J, Meyer C, Hapke U, John U. Screening for mental health: validity of the MHI-5 using DSM-IV Axis I psychiatric disorders as gold standard. Psychiatry Research. 2001 Dec 31;105(3):243–53.

20. Coast J, Peters TJ, Natarajan L, Sproston K, Flynn T. An assessment of the construct validity of the descriptive system for the ICECAP capability measure for older people. Qual Life Res. 2008; 17(7): 967-76.

21. Cardol M, de Haan RJ, van den Bos GA, de Jong BA, de Groot IJ. The development of a handicap assessment questionnaire: the Impact on Participation and Autonomy (IPA). Clin Rehabil. 1999; 13(5): 411-9.

22. Smith BW, Dalen J, Wiggins K, Tooley E, Christopher P, Bernard J. The brief resilience scale: assessing the ability to bounce back. Int J Behav Med. 2008; 15(3): 194-200.

23. Lloyd H, Fosh B, Whalley B, Byng R, Close J. Validation of the person-centred coordinated care experience questionnaire (P3CEQ). International Journal for Quality in Health Care. 2019 Aug 1;31(7):506–12.

24. Uijen AA, Schellevis FG, van den Bosch WJ, Mokkink HG, van Weel C, Schers HJ. Nijmegen Continuity Questionnaire: development and testing of a questionnaire that measures continuity of care. J Clin Epidemiol. 2011; 64(12): 1391-9.

25. McGuiness C, Sibthorpe B. Development and initial validation of a measure of coordination of health care. Int J Qual Health Care. 2003; 15(4): 309-18.

26. Bouwmans C, Hakkaart-van Roijen L, Koopmanschap M, Krol M, Severens H, Brouwer W. Handleiding iMTA Medical Cost Questionnaire (iMCQ). Rotterdam: iMTA: Erasmus Universiteit; 2013.

For peer review only - http://bmjopen.bmj.com/site/about/guidelines.xhtml

27. Pearlin LI, Schooler C. The Structure of Coping. Journal of Health and Social Behavior. 1978;19(1):2–21.

28. Krska J, Morecroft CW, Rowe PH, Poole H. Measuring the impact of long-term medicines use from the patient perspective. Int J Clin Pharm. 2014 Aug 1;36(4):675–8.

29. Kanters TA, Bouwmans CAM, Linden N van der, Tan SS, Roijen LH. Update of the Dutch manual for costing studies in health care. PLoS ONE. 2017 Nov 9;12(11):e0187477.

30. G-Standaard Flex — Z-Index [Internet]. [cited 2021 Feb 2]. Available from: https://www.z-index.nl/producten-diensten/g-standaard-flex.

31. Vektis - business intelligence centrum voor de zorg | Vektis.nl [Internet]. Vektis. [cited 2021 Feb 2]. Available from: https://www.vektis.nl.

32. Rosenbaum PR, Rubin DB. The Central Role of the Propensity Score in Observational Studies for Causal Effects. Biometrika. 1983;70(1):41–55.

33. Austin PC, Stuart EA. Moving towards best practice when using inverse probability of treatment weighting (IPTW) using the propensity score to estimate causal treatment effects in observational studies. Stat Med. 2015 Dec 10;34(28):3661–79.

34. Rubin DB. Using Propensity Scores to Help Design Observational Studies: Application to the Tobacco Litigation. Health Services & Outcomes Research Methodology. 2001 Dec 1;2(3):169–88.

35. Stuart EA. Matching methods for causal inference: A review and a look forward. Stat Sci. 2010 Feb 1;25(1):1–21.

36. Mölken MR, Karimi M, Leijten F, Hoedemakers M, Looman W, Islam K, et al. Comparing patients' and other stakeholders' preferences for outcomes of integrated care for multimorbidity: a discrete choice experiment in eight European countries. BMJ Open. 2020 Oct 1;10(10):e037547.

37. Publications [Internet]. Selfie 2020 -. [cited 2021 Feb 1]. Available from: https://www.selfie2020.eu/publications/.

For peer review only

For peer review only - http://bmjopen.bmj.com/site/about/guidelines.xhtml

38. Baxter S, Johnson M, Chambers D, Sutton A, Goyder E, Booth A. The effects of integrated care: a systematic review of UK and international evidence. BMC Health Serv Res. 2018 May 10;18(1):350.

39. Wodchis WP, Dixon A, Anderson GM, Goodwin N. Integrating care for older people with complex needs: key insights and lessons from a seven-country cross-case analysis. International Journal of Integrated Care. 2015;15(6).

40. Blom JW, Van den Hout WB, Den Elzen WPJ, Drewes YM, Bleijenberg N, Fabbricotti IN, et al. Effectiveness and cost-effectiveness of proactive and multidisciplinary integrated care for older people with complex problems in general practice: an individual participant data meta-analysis. Age and Ageing. 2018 Sep 1;47(5):705–14.

41. Bauhoff S. Systematic self-report bias in health data: impact on estimating cross-sectional and treatment effects. Health Serv Outcomes Res Method. 2011 Jul 1;11(1):44–53.

42. Liljas AEM, Brattström F, Burström B, Schön P, Agerholm J. Impact of Integrated Care on Patient-Related Outcomes Among Older People – A Systematic Review. International Journal of Integrated Care. 2019 Jul 24;19(3):6.

43. Marsh K, IJzerman M, Thokala P, Baltussen R, Boysen M, Kaló Z, et al. Multiple Criteria Decision Analysis for Health Care Decision Making—Emerging Good Practices: Report 2 of the ISPOR MCDA Emerging Good Practices Task Force. Value in Health. 2016 Mar 1;19(2):125–37.

44. Marsh KD, Sculpher M, Caro JJ, Tervonen T. The Use of MCDA in HTA: Great Potential, but More Effort Needed. Value in Health. 2018 Apr 1;21(4):394–7.

45. Baltussen R, Marsh K, Thokala P, Diaby V, Castro H, Cleemput I, et al. Multicriteria Decision Analysis to Support Health Technology Assessment Agencies: Benefits, Limitations, and the Way Forward. Value in Health. 2019 Nov 1;22(11):1283–8.

46. Sculpher M, Claxton K, Pearson SD. Developing a Value Framework: The Need to Reflect the Opportunity Costs of Funding Decisions. Value in Health. 2017 Feb;20(2):234–9.

For peer review only - http://bmjopen.bmj.com/site/about/guidelines.xhtml

For peer review only

Figure caption

Figure 1. Flow chart of patient participation

For peer review only

For peer review only - http://bmjopen.bmj.com/site/about/guidelines.xhtml

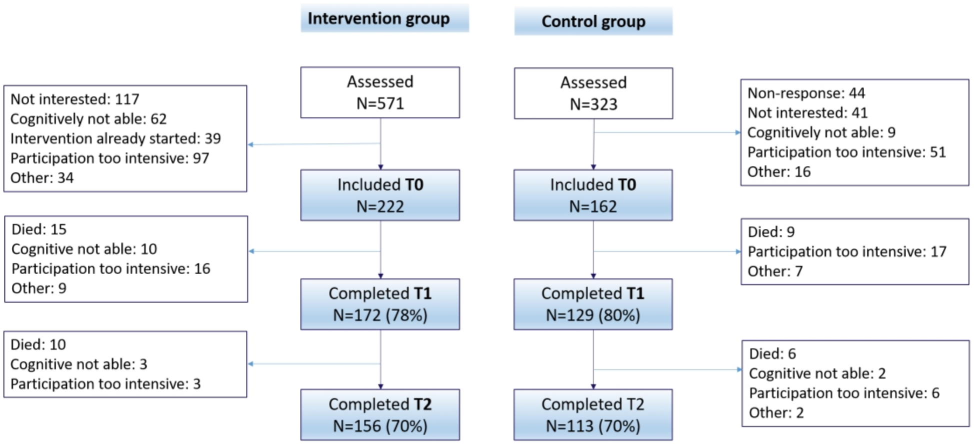

Figure 1. Flowchart of patient participation

985x471mm (59 x 59 DPI)

Supplementary files

Appendix 1: Equation relative standardization 2
Appendix 2. Relative DCE weights (0-1) (SE) of the outcomes used in the MCDA by type of stakeholder 3
Appendix 3: Graph common support 4
Appendix 4: Predicted mean performance scores of all outcome measures on their natural scale...... 5
Appendix 5: Comparison of baseline characteristics and outcome scores of dropouts versus non-dropouts........ 6
Appendix 6a: Value scores in the Multi-Criteria Decision Analysis at 6 months without costs 8
Appendix 6b: Value scores in the Multi-Criteria Decision Analysis at 12 months without costs 9

For peer review only

For peer review only - http://bmjopen.bmj.com/site/about/guidelines.xhtml

Appendix 1: Equation relative standardization

$$S_{aj} = \frac{x_{aj}}{\left(x_{aj}^2 + x_{bj}^2\right)^{1/2}}$$

Where

x = predicted mean score on the natural scale

a = the intervention group

b = the control group

j = outcome measure *j*

Note: For all outcomes in the MCDA, a higher score indicates better performance. To achieve this, in the above-mentioned equation, x is replaced by $1/x$ for outcomes where a higher score on the natural scale indicates a worse performance (e.g., costs).

For peer review only

For peer review only - http://bmjopen.bmj.com/site/about/guidelines.xhtml

Appendix 2. Relative DCE weights (0-1) (SE) of the outcomes used in the MCDA by type of stakeholder

	**Patients (n=156)**	**Partners (n=158)**	**Professionals (n=155)**	**Payers (n=104)**	**Policymakers (n=151)**
Physical functioning	0.16 (0.15)	0.11 (0.12)	0.12 (0.12)	0.14 (0.13)	0.14 (0.14)
Psychological well-being	0.17 (0.15)	0.15 (0.14)	0.18 (0.16)	0.18 (0.17)	0.15 (0.15)
Enjoyment of life	0.23 (0.18)	0.25 (0.19)	0.22 (0.19)	0.24 (0.23)	0.22 (0.19)
Social participation & relationships	0.08 (0.10)	0.09 (0.11)	0.11 (0.11)	0.10 (0.11)	0.10 (0.10)
Resilience	0.15 (0.13)	0.14 (0.13)	0.13 (0.12)	0.11 (0.10)	0.14 (0.13)
Person-centeredness	0.08 (0.10)	0.08 (0.10)	0.08 (0.09)	0.06 (0.08)	0.08 (0.09)
Continuity of care	0.10 (0.11)	0.12 (0.12)	0.11 (0.11)	0.08 (0.09)	0.10 (0.10)
Total health and social care costs	0.03 (0.08)	0.06 (0.09)	0.06 (0.09)	0.08 (0.08)	0.07 (0.09)

Note: Numbers in parentheses (n) by stakeholders indicate the number of participants included in the weight elicitation study

For peer review only

Appendix 3: Graph common support

Graph 1: Common Support of the propensity scores when matching includes the baseline values of the following variables: age, gender, marital status, living situation, educational level, smoking status, and total health and social care costs (minus medication) of three months prior to baseline as a proxy for severity

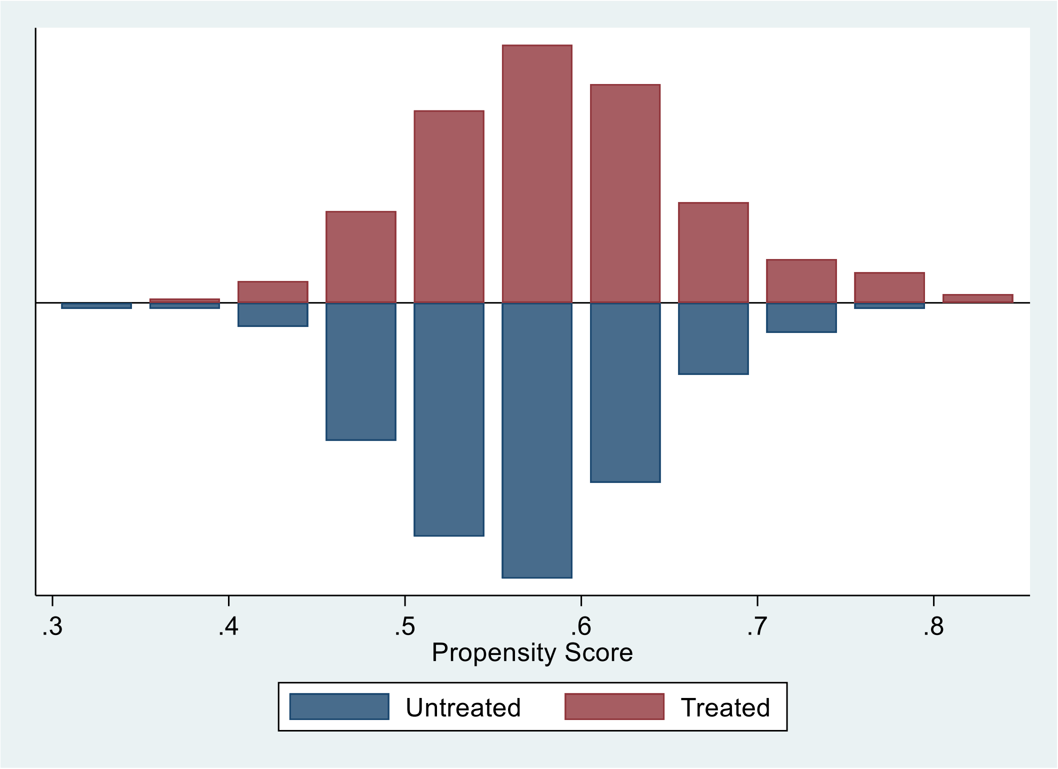

only

For peer review only - http://bmjopen.bmj.com/site/about/guidelines.xhtml

Appendix 4: Predicted mean performance scores of all outcome measures on their natural scale

	T0#		T1		T2	
**Outcomes measures**	**CCFE (n=222)**	**UC (n=162)**	**CCFE (n=172)**	**UC (n=129)**	**CCFE (n=156)**	**UC (n=113)**
**Health & Well-being**						
Physical functioning (0-15) ^	4.383	4.383	5.126	4.653	5.715	5.328
Psychological well-being (0-100)	71.351	71.351	70.707	71.010	69.998	71.256
Enjoyment of life (1-4)	2.820	2.820	2.917	2.741	2.874	2.839
Social relationships & participation (7-35) ^	9.167	9.167	9.273	9.579	9.424	9.779
Resilience (6-30)	19.317	19.317	19.299	19.108	19.347	19.426
**Experience of care**						
Person-centeredness (0-18)	11.729	11.729	12.828	11.353	13.060	11.727
Continuity of care (1-5)	3.672	3.672	3.771	3.653	3.833	3.704
**Costs**						
Total health and social care costs ^	5453	5453	10858	10199	22676	20659
**Additional frail elderly-specific outcomes**						
Autonomy (7-35)	22.284	22.284	21.785	22.306	21.790	22.834
Burden of medication (0-10) ^	2.088	2.088	2.221	1.878	2.263	1.480

# Note that the intervention coefficient was included in the calculation of the mean performance scores in the control group, assuming the control group had the same baseline scores as the intervention group to make it possible to directly compare the intervention and control group. CCFE=Care Chain Frail Elderly. UC=Usual care.

For peer review only - http://bmjopen.bmj.com/site/about/guidelines.xhtml

Appendix 5: Comparison of baseline characteristics and outcome scores of dropouts versus non-dropouts

	Across CCFE and UC			CCFE separately			UC separately		
**Baseline characteristics**	**Dropouts (n=115)**	**Non-dropouts (n=269)**	**P-value**	**Dropouts (n=66)**	**Non-dropouts (n=156)**	**P-value**	**Dropouts (n=49)**	**Non-dropouts (n=113)**	**P-value**
**Intervention group**	66 (57%)	156 (58%)	0.913	-	-	-	-	-	-
**Age**	85.00	83.56	0.036*	83.91	83.21	0.449	86.47	84.05	0.016*
**Gender** (female)	61%	65%	0.434	62%	64%	0.779	59%	66%	0.381
**Living situation**			0.926			0.842			0.711
Independent, alone	57%	57%		53%	54%		63%	60%	
With other(s)	43%	43%		47%	46%		37%	40%	
**Marital status**			0.162			0.149			0.734
Single, never married	1%	5%		0%	5%		2%	4%	
Married or living together	41%	44%		44%	45%		37%	42%	
Widow(er)	53%	45%		52%	42%		55%	49%	
Divorced	5%	7%		5%	8%		6%	4%	
**Educational level**			0.923			0.839			0.981
Low	70%	71%		68%	71%		71%	70%	
Middle	19%	17%		23%	19%		14%	15%	
High	11%	12%		9%	10%		14%	15%	
**Smoking** (yes)	15%	11%	0.269	18%	13%	0.299	10%	8%	0.641
**Baseline outcome scores**									
Physical functioning (0-15) ^	5.18	4.10	0.001*	4.89	4.17	0.081	5.56	4.02	0.003*
Psychological well-being (0-100)	69.37	72.39	0.145	68.06	72.74	0.092	71.17	71.89	0.815
Enjoyment of life (1-4)	2.81	2.89	0.351	2.74	2.85	0.349	2.90	2.95	0.725

For peer review only - http://bmjopen.bmj.com/site/about/guidelines.xhtml

	Across CCFE and UC			CCFE separately			UC separately		
**Baseline characteristics**	**Dropouts (n=115)**	**Non-dropouts (n=269)**	**P-value**	**Dropouts (n=66)**	**Non-dropouts (n=156)**	**P-value**	**Dropouts (n=49)**	**Non-dropouts (n=113)**	**P-value**
Social relationships and participation (7-35) ^	8.86	8.55	0.461	9.48	9.03	0.451	8.00	7.88	0.831
Resilience (6-30)	18.78	19.38	0.212	18.97	19.46	0.448	18.52	19.27	0.298
Autonomy (7-35)	21.81	22.35	0.245	21.89	22.45	0.386	21.69	22.21	0.452
Person-centeredness (0-18)	12.29	11.97	0.473	11.86	11.66	0.730	12.88	12.4	0.481
Continuity of care (1-5)	3.70	3.72	0.838	3.65	3.69	0.743	3.78	3.77	0.939
Burden of medication (0-10) ^	2.17	2.24	0.809	2.12	2.07	0.908	2.22	2.47	0.604
Total costs 3 months prior to the study (€) ^	5,989	5,112	0.247	5,797	5,308	0.635	6,248	4,842	0.208

*^ Higher score indicating worse performance. * p<0.05. CCFE=Care Chain Frail Elderly. UC=Usual care.*

For peer review only - http://bmjopen.bmj.com/site/about/guidelines.xhtml

Appendix 6a: Value scores in the Multi-Criteria Decision Analysis at 6 months without costs

			Patients		Partners		Professionals		Payers		Policymakers	
**Outcome measures**	**Standardised performance score#**		**Weighted score**		**Weighted score**		**Weighted score**		**Weighted score**		**Weighted score**	
	**CCFE**	**UC**	**CCFE**	**UC**	**CCFE**	**UC**	**CCFE**	**UC**	**CCFE**	**UC**	**CCFE**	**UC**
Physical functioning	0.672	0.740	0.111	0.122	0.079	0.087	0.085	0.094	0.103	0.114	0.101	0.111
Psychological well-being	0.706	0.709	0.124	0.124	0.113	0.113	0.134	0.134	0.140	0.140	0.114	0.114
Enjoyment of life	0.729	0.685	0.173	0.162	0.194	0.182	0.169	0.159	0.192	0.181	0.172	0.162
Social relationships and participation	0.718	0.696	0.059	0.057	0.069	0.067	0.083	0.081	0.079	0.076	0.077	0.075
Resilience	0.711	0.704	0.110	0.109	0.106	0.105	0.097	0.096	0.086	0.085	0.107	0.106
Person-centeredness	0.749	0.663	0.062	0.055	0.064	0.056	0.063	0.056	0.049	0.044	0.064	0.057
Continuity of care	0.718	0.696	0.074	0.072	0.092	0.089	0.083	0.081	0.063	0.061	0.077	0.075
**Overall value scores**			**0.712**	**0.701**	**0.715**	**0.698**	**0.714**	**0.700**	**0.713**	**0.701**	**0.713**	**0.700**

# colour scheme ranges from red (lowest score) to green (highest score). CCFE=Care Chain Frail Elderly. UC=Usual care.

For peer review only - http://bmjopen.bmj.com/site/about/guidelines.xhtml

Appendix 6b: Value scores in the Multi-Criteria Decision Analysis at 12 months without costs

			Patients		Partners		Professionals		Payers		Policymakers	
**Outcome measures**	**Standardised performance score#**		**Weighted score**		**Weighted score**		**Weighted score**		**Weighted score**		**Weighted score**	
	**CCFE**	**UC**	**CCFE**	**UC**	**CCFE**	**UC**	**CCFE**	**UC**	**CCFE**	**UC**	**CCFE**	**UC**
Physical functioning	0.682	0.731	0.112	0.121	0.080	0.086	0.086	0.092	0.105	0.113	0.103	0.110
Psychological well-being	0.701	0.713	0.123	0.125	0.112	0.114	0.133	0.135	0.139	0.141	0.113	0.115
Enjoyment of life	0.711	0.703	0.169	0.167	0.189	0.187	0.165	0.163	0.188	0.185	0.168	0.166
Social relationships and participation	0.720	0.694	0.059	0.057	0.069	0.066	0.083	0.080	0.079	0.076	0.077	0.075
Resilience	0.706	0.709	0.109	0.110	0.105	0.106	0.097	0.097	0.085	0.086	0.106	0.107
Person-centeredness	0.744	0.668	0.061	0.055	0.063	0.057	0.063	0.056	0.049	0.044	0.064	0.057
Continuity of care	0.719	0.695	0.074	0.072	0.092	0.089	0.083	0.080	0.063	0.061	0.077	0.075
**Overall value scores**			**0.708**	**0.706**	**0.710**	**0.704**	**0.710**	**0.704**	**0.708**	**0.706**	**0.709**	**0.705**

colour scheme ranges from red (lowest score) to green (highest score). CCFE=Care Chain Frail Elderly. UC=Usual care.

For peer review only - http://bmjopen.bmj.com/site/about/guidelines.xhtml

The RECORD statement – checklist of items, extended from the STROBE statement, that should be reported in observational studies using routinely collected health data.

	Item No.	STROBE items	Location in manuscript where items are reported	RECORD items	Location in manuscript where items are reported
**Title and abstract**					
	1	(a) Indicate the study's design with a commonly used term in the title or the abstract (b) Provide in the abstract an informative and balanced summary of what was done and what was found	1,3	RECORD 1.1: The type of data used should be specified in the title or abstract. When possible, the name of the databases used should be included.<br><br>RECORD 1.2: If applicable, the geographic region and timeframe within which the study took place should be reported in the title or abstract.<br><br>RECORD 1.3: If linkage between databases was conducted for the study, this should be clearly stated in the title or abstract.	1,3
**Introduction**					
Background rationale	2	Explain the scientific background and rationale for the investigation being reported	5,6		
Objectives	3	State specific objectives, including any prespecified hypotheses	6		
**Methods**					
Study Design	4	Present key elements of study design early in the paper	7,8		
Setting	5	Describe the setting, locations, and relevant dates, including periods of recruitment, exposure, follow-up, and data collection	7-9		

For peer review only

Participants	6	*(a) Cohort study* - Give the eligibility criteria, and the sources and methods of selection of participants. Describe methods of follow-up<br>*Case-control study* - Give the eligibility criteria, and the sources and methods of case ascertainment and control selection. Give the rationale for the choice of cases and controls<br>*Cross-sectional study* - Give the eligibility criteria, and the sources and methods of selection of participants<br><br>*(b) Cohort study* - For matched studies, give matching criteria and number of exposed and unexposed<br>*Case-control study* - For matched studies, give matching criteria and the number of controls per case	8-9<br><br>10	RECORD 6.1: The methods of study population selection (such as codes or algorithms used to identify subjects) should be listed in detail. If this is not possible, an explanation should be provided.<br><br>RECORD 6.2: Any validation studies of the codes or algorithms used to select the population should be referenced. If validation was conducted for this study and not published elsewhere, detailed methods and results should be provided.<br><br>RECORD 6.3: If the study involved linkage of databases, consider use of a flow diagram or other graphical display to demonstrate the data linkage process, including the number of individuals with linked data at each stage.	
Variables	7	Clearly define all outcomes, exposures, predictors, potential confounders, and effect modifiers. Give diagnostic criteria, if applicable.	9	RECORD 7.1: A complete list of codes and algorithms used to classify exposures, outcomes, confounders, and effect modifiers should be provided. If these cannot be reported, an explanation should be provided.	
Data sources/ measurement	8	For each variable of interest, give sources of data and details of methods of assessment (measurement).<br>Describe comparability of assessment methods if there is more than one group	9-10		

For peer review only - http://bmjopen.bmj.com/site/about/guidelines.xhtml

Bias	9	Describe any efforts to address potential sources of bias	10		
Study size	10	Explain how the study size was arrived at	see study protocol		
Quantitative variables	11	Explain how quantitative variables were handled in the analyses. If applicable, describe which groupings were chosen, and why	10		
Statistical methods	12	(a) Describe all statistical methods, including those used to control for confounding<br>(b) Describe any methods used to examine subgroups and interactions<br>(c) Explain how missing data were addressed<br>(d) *Cohort study* - If applicable, explain how loss to follow-up was addressed<br>*Case-control study* - If applicable, explain how matching of cases and controls was addressed<br>*Cross-sectional study* - If applicable, describe analytical methods taking account of sampling strategy<br>(e) Describe any sensitivity analyses	10-11<br>11		
Data access and cleaning methods		..		RECORD 12.1: Authors should describe the extent to which the investigators had access to the database population used to create the study population.	NA

For peer review only

				RECORD 12.2: Authors should provide information on the data cleaning methods used in the study.	NA
Linkage		..		RECORD 12.3: State whether the study included person-level, institutional-level, or other data linkage across two or more databases. The methods of linkage and methods of linkage quality evaluation should be provided.	NA
**Results**					
Participants	13	(a) Report the numbers of individuals at each stage of the study (*e.g.*, numbers potentially eligible, examined for eligibility, confirmed eligible, included in the study, completing follow-up, and analysed)<br>(b) Give reasons for non-participation at each stage.<br>(c) Consider use of a flow diagram	12	RECORD 13.1: Describe in detail the selection of the persons included in the study (*i.e.*, study population selection) including filtering based on data quality, data availability and linkage. The selection of included persons can be described in the text and/or by means of the study flow diagram.	13
Descriptive data	14	(a) Give characteristics of study participants (*e.g.*, demographic, clinical, social) and information on exposures and potential confounders<br>(b) Indicate the number of participants with missing data for each variable of interest<br>(c) *Cohort study* - summarise follow-up time (*e.g.*, average and total amount)	12<br>12<br>8		
Outcome data	15	*Cohort study* - Report numbers of outcome events or summary measures over time<br>*Case-control study* - Report numbers in each exposure	13		

For peer review only

		category, or summary measures of exposure<br>*Cross-sectional study* - Report numbers of outcome events or summary measures			
Main results	16	(a) Give unadjusted estimates and, if applicable, confounder-adjusted estimates and their precision (e.g., 95% confidence interval). Make clear which confounders were adjusted for and why they were included<br>(b) Report category boundaries when continuous variables were categorized<br>(c) If relevant, consider translating estimates of relative risk into absolute risk for a meaningful time period	13		
Other analyses	17	Report other analyses done—e.g., analyses of subgroups and interactions, and sensitivity analyses	13		
**Discussion**					
Key results	18	Summarise key results with reference to study objectives	14		
Limitations	19	Discuss limitations of the study, taking into account sources of potential bias or imprecision. Discuss both direction and magnitude of any potential bias	15	RECORD 19.1: Discuss the implications of using data that were not created or collected to answer the specific research question(s). Include discussion of misclassification bias, unmeasured confounding, missing data, and changing eligibility over time, as they pertain to the study being reported.	NA
Interpretation	20	Give a cautious overall interpretation of results considering objectives,	14,15		

For peer review only

		limitations, multiplicity of analyses, results from similar studies, and other relevant evidence			
Generalisability	21	Discuss the generalisability (external validity) of the study results	15		
**Other Information**					
Funding	22	Give the source of funding and the role of the funders for the present study and, if applicable, for the original study on which the present article is based	4		
Accessibility of protocol, raw data, and programming code		..		RECORD 22.1: Authors should provide information on how to access any supplemental information such as the study protocol, raw data, or programming code.	

*Reference: Benchimol EI, Smeeth L, Guttmann A, Harron K, Moher D, Petersen I, Sørensen HT, von Elm E, Langan SM, the RECORD Working Committee. The REporting of studies Conducted using Observational Routinely-collected health Data (RECORD) Statement. *PLoS Medicine* 2015; in press.

*Checklist is protected under Creative Commons Attribution (CC BY) license.

For peer review only
